# Exploring the transcriptome of non-model oleaginous microalga *Dunaliella tertiolecta* through high-throughput sequencing and high performance computing

**DOI:** 10.1186/s12859-017-1551-x

**Published:** 2017-02-22

**Authors:** Lina Yao, Kenneth Wei Min Tan, Tin Wee Tan, Yuan Kun Lee

**Affiliations:** 1 Department of Microbiology and Immunology, Yong Loo Lin School of Medicine, National University of Singapore, Singapore, 117545 Singapore; 2 Department of Biochemistry, Yong Loo Lin School of Medicine, National University of Singapore, Singapore, 117596 Singapore; 3 National Supercomputing Centre (NSCC), Singapore, 138632 Singapore

**Keywords:** Microalgae, *Dunaliella tertiolecta*, HPC, Transcriptome, RNA-Seq

## Abstract

**Background:**

RNA-Seq technology has received a lot of attention in recent years for microalgal global transcriptomic profiling. It is widely used in transcriptome-wide analysis of gene expression., particularly for microalgal strains with potential as biofuel sources. However, insufficient genomic or transcriptomic information of non-model microalgae has limited the understanding of their regulatory mechanisms and hampered genetic manipulation to enhance biofuel production. As such, an optimal microalgal transcriptomic database construction is a subject of urgent investigation.

**Results:**

*Dunaliella tertiolecta*, a non-model oleaginous microalgal species, was sequenced via Illumina MISEQ and HISEQ 4000 in RNA-Seq studies. The high quality high-throughout sequencing data were explored using high performance computing (HPC) in a petascale data center and subjected to *de novo* assembly and parallelized mpiBLASTX search with multiple species. As a result, a transcriptome database of 17,845 was constructed (~95% completeness). This enlarged database constructed fueled the RNA-Seq data analysis, which was validated by a nitrogen deprivation (ND) study that induces triacylglycerol (TAG) production.

**Conclusions:**

The new paralleled assembly and annotation method under HPC presented here allows the solution of large-scale data processing problems in acceptable computation time. There is significant increase in the number of transcriptomic data achieved and observable heterogeneity in the performance to identify differentially expressed genes in the ND treatment paradigm. The results provide new insights as to how response to ND treatment in microalgae is regulated. ND analyses highlight the advantages of this database generated in this study that could also serve as a useful resource for future gene manipulation and transcriptome-wide analysis. We thus demonstrate the usefulness of exploring the transcriptome as an informative platform for functional studies and genetic manipulations in similar species.

**Electronic supplementary material:**

The online version of this article (doi:10.1186/s12859-017-1551-x) contains supplementary material, which is available to authorized users.

## Background

RNA-Seq is a recently developed approach for transcriptomic profiling, which uses deep-sequencing technologies to elucidate the complexity of eukaryotic transcriptomes [1]. It has been applied in quantifying transcriptional expression in microalgae mutants and microalgae cultured under different culture conditions to elucidate their metabolic regulatory mechanism. Various high-throughput sequencing technologies have been used for RNA-Seq, such as Roche 454 (Life Science), Ion Torrent ((PGM), Thermo Fisher), SOLiD (Applied Biosystems), and MISEQ/HISEQ (Illumina) systems. Following sequencing, the output reads are either aligned to a reference genome or transcriptome, or assembled a transcription map for each gene [1]. RNA-Seq based transcriptome assemblers have been developed in the past few years, which are largely reference-based [2–4] and highly dependent on a high-quality reference genome. As of now, only few comprehensively annotated model microalgae genomes are available, such as *Coccomyxa subellipsoidea* C-169 v2 [5], *Chlorella variabilis* NC64A v1 [6], *Chlamydomonas reinhardtii* v4 [7], *Micromonas pusilla* RCC299 v3 [8], *Ostreococcus lucimarinus* v2 [9], and *Volvox carteri* [10]. However, a large amount of microalgae with unique traits, which have advantages over model microalgae as feedstock for biofuel production and many other valuable biomolecules are not completely sequenced, thus has limited the potential of genetic engineering and comparative analysis of the transcriptome data [11]. Fortunately, in recent years, workflows for transcriptome analysis on non-model microalgae have been developed [11–16], which paves the way for the development of elite algal strains for biofuels production. *De novo* assembly approach is applied for species with no reliable reference genome. Currently, a number of *de novo* assemblers are available in the market, such as Velvet [17], ABySS [18], SOAPdenovo [19], Trinity [3, 20], and Bridger [2]. However, some of them rely too much on the genome-assembly methods, or are too memory/time consuming. Among them, Trinity assembler was reported to have the highest number of assembled transcripts matching the non-redundant (Nr) database [2, 15, 21].

With the advances in sequencing technologies that most widely using Illumina MISEQ/HISEQ today, and as sequencing depth becomes higher, the assembly of raw data now requires high capacity processing, which still could not be fulfilled by off-the-shelf PCs [22]. Herein, high performance computing (HPC) in a petascale data center was introduced in our study. The use of HPC has opened up great opportunities for applications in many areas, including next-generation sequencing (NGS) data analysis [23].

*Dunaliella tertiolecta*, a non-model unicellular halophilic green alga that has fast growth rate and high accumulation of lipids, was used as the experimental organism in this study [15]. Recently, the whole genome sequence of the *D. salina* v1.0 was released (genome. jgi-psf.org), which aids in our comparison study as the most related species. Equipped with more input sequencing data (from Illumina HISEQ 4000), advanced *de novo* assembler, a wider reference species annotation database (all plants and bacterial proteins), and HPC in high performance data center, a much more complete *D. tertiolecta* transcriptome database (~95% of the total gene numbers) was constructed herein and applied in a case study of RNA-Seq data analysis from nitrogen-deprived cells. From the nitrogen deprivation (ND) study, potential regulatory mechanisms of cell growth and triacylglycerol (TAG) accumulation were proposed. Further, alternative-splicing variants in *D. tertiolecta* was predicted and compared with related species for the first time. This approach could be applied to other non-model microalgae for further applications.

## Methods

### Cultivation of microalgal samples

The algal culture *D. tertiolecta* strain UTEX LB-999 was obtained from the UTEX Culture Collection of Algae (University of Texas at Austin, TX) and cultured in 250 mL flasks containing ATCC-1174 DA liquid medium (American Type Culture Collection at Manassas, Virginia) with 0.5 M NaCl, 5 mM KNO_3_ under 30 μE m^-2^ s^-1^ as low light condition (with 400 μE m^-2^ s^-1^ for one set of high light culture. The rest cultures intended for transcriptomic data assembly were cultured under low light condition with the other culture conditions unchanged, as detailed in Table 1). For the ND study, *D. tertiolecta* were cultured in reduced nitrogen (0.5 mM KNO_3_), with the other culture conditions unchanged. Biological duplicates of ND and its WT were cultured for subsequent sequencing.

**Table 1:** Input raw data and post-analyzed data from MISEQ and HISEQ

Data name	Data source	Number of protein-coding contigs
Dt_G (HISEQ 4000)	Yao et al. 2016 [63]	27,797
Dt_Shin (MISEQ)	Shin et al. 2015 [15]	13,861
Dt_KR (MISEQ)	Tan et al. 2016 [58]	25,475
Dt_v10 (MISEQ)	Yao et al. 2015 [16]	20,229
Merged contigs	-	87,197
Non-redundant contigs	-	17,845

### Measurement of dry cell weight, TAG and fatty acid content

Dry cell weight (DCW) measurement was performed by harvesting 10 mL of cells and collected by filtration on pre-weighed Advantec GB-140 filter paper (0.4 μm pore size; diameter 47 mm). The filter paper was then washed with isotonic 0.5 M ammonium formate (40 mL) to remove salts without causing the cells to burst. Cells captured on filter paper discs were dried in oven at 95 °C, and measured for DCW.

A modified Nile red staining method [16] was used to quantify intracellular TAGs. Briefly, cells were harvested by centrifugation (3000 *g* for 10 min at 4 °C), supernatant was removed and the pellet resuspended in fresh 0.5 M ATCC-1174 DA media to an OD_680_ of 0.3. Two hundred microliters of triolein standards (40, 20, 10, 5, 2.5, 0 μg/mL) and cell suspensions were loaded as technical triplicates onto a 96-well black, clear bottom plate (CLS3603; Sigma-Aldrich). Prior to staining, Nile red stock is diluted in acetone to obtain a working solution (25 μg/mL), and 2 μL of the Nile red working solution is added to each well of sample and standard, followed by a 5 min incubation in the dark. Fluorescence of each sample was detected using a microplate reader (Infinite M200 PRO, Tecan) at excitation and emission wavelengths of 524 nm and 586 nm. Fluorescence imaging of Nile Red-stained cells was performed with an automated fluorescence microscope (Olympus BX63). Acquisition and processing of data was done using the cellSens software.

To analyze the accumulation of total lipids, cells were harvested, snap-frozen in liquid nitrogen and stored at -80 °C until analysis. Frozen culture samples were lyophilized by freeze-drying and lipids were extracted by hexane using direct transesterification [24] as it was reported to be a convenient and accurate method for analyzing total fatty acids [25]. Biomass quantities of between 5 and 10 mg of biomass were weighed into glass 55-mL PYREX culture tubes with polytetrafluoroethylene (PTFE)-lined phenolic caps (25 mm diameter × 150 mm height, PYREX #9826-25, Corning). To each sample, 0.2 mL of chloroform-methanol (2:1, *v*/*v*) was added and mixed by vortexing, followed by simultaneous transesterification of lipids with 0.3 mL of 1.25 M methanolic HCl and vortexed to mix. An internal standard (100 μg Methyl tridecanoate, C13-Fatty Acid Methyl Ester, C13-FAME; Cat. no. 91558, Sigma-Aldrich) was included to correct for the loss of FAME during the reaction, and to correct for subsequent incomplete extraction of hexane [26]. The culture tube was then incubated in a 50 °C waterbath overnight. After 24 hours, 1 mL of hexane was added and mixed by vortex, and incubated at room temperature for 1 h. The upper organic phase containing FAMEs was removed using a glass pipette, filtered through a 0.22-μm PTFE syringe filter (Agilent Technologies), and collected in a 250-μL glass vial insert (Part no. 5181-1270, Agilent Technologies). FAME extracts were injected into a GC system (Model 7890B, Agilent Technologies) equipped with an Agilent Agilent HP-5 ms Ultra Inert column (30 m x 250 μm x 0.25 μm) (Cat. no. 19091S-433UI, Agilent Technologies) interfaced with a mass spectrometric detector (Model 5977A, Agilent Technologies). Injection volume was set at 1 μL with a 5:1 split ratio at a GC inlet temperature of 250 °C. Helium was used as the carrier gas in a fixed flow of 1 mL/min throughout. Temperature program is as follows: initial oven temperature of 70 °C held for 3 mins, ramp to 130 °C at 20 °C/min, 178 °C at 4 °C/min, 190 °C at 1 °C/min, and 290 °C at 10 °C/min. The total run time was 40 min. Shifting of retention times (RTs) were eliminated by comparing the RTs of each FA compound to the C13-FAME internal standard. Analysis was performed using the MassHunter WorkStation Qualitative Analysis B.07.00 software (Agilent Technologies) and compounds were identified with the NIST mass spectral library (National Institute of Standards and Technology, Data Version: NIST 14).

### Preparation for sequencing and *de novo* transcriptome assembly

Cells were harvested at different culture stages by Allegra® X-30 centrifuge (Beckman Coulter) at 4000 × g for 10 min at 4 °C. The cell pellets were immediately frozen in liquid nitrogen and total RNA was extracted using RNeasy plant RNA Mini kit (Qiagen). After cDNA libraries were constructed [16], validation and quality assessment of each library was performed from gel electrophoresis and bioanalyzer (Agilent Technologies; Santa Clara, CA, USA). The concentration of each library was quantified via KAPA Library Quantification Kit (Illumina® platforms). The resulting libraries were sequenced by Illumina MISEQ sequencer (KR represents data from *D. tertiolecta* ND and highlight cultures) and Illumina HISEQ sequencer (G represents data from *D. tertiolecta* mutant cultures).

After sequencing reads were trimmed by QA/QC, Trinity assembler v2.2.0 was used to obtain strand-specific paired-end short reads data, with the default setting. To have a more complete transcriptome database, draft datasets constructed by Shin et al. [15] (http://cholab.or.kr/data/) and Yao et al. [16] from Illumina MISEQ sequencer were also adopted and compared in the following pipeline.

### Annotation of the transcriptome

To have the functional annotation of the assembled transcripts, Basic Local Alignment Search Tool (BLAST suite), was used to compare against the ‘best’ proteins in the comprehensively annotated plant and bacterial Nr database from NCBI website (http://www.ncbi.nlm.nih.gov/refseq/). Protein IDs and their hypothetical function names were obtained for corresponding transcripts. To avoid multiple counting of contigs, only the best alignment (‘top hit’) from BLASTX was kept.

Generally, we filtered our BLASTX results using a three-step criterion: 1) The best alignment was chosen with E-value < = 1E-10; 2) Length percentage of the query sequence > = 80% of the subject protein sequence alignment; 3) Redundant contigs with the same ncbi_proteinID were deleted. The resulting transcripts were served as the protein coding sequences (Dtertiolecta_v11.transcript_primaryTranscriptOnly.fa, short as ‘Dt_v11’ below). Transcripts associated with a Kyoto Encyclopedia of Genes and Genomes (KEGG) metabolic pathway or a Gene Ontology (GO) biological process were predicted to represent a certain expression pattern [12]. To identify it, we applied the online KEGG database for conversion of BLASTX results (ncbi_proteinID) into KEGG gene, KEGG Orthology (KO), GO and GO definition through KEGG (http://www.kegg.jp) and GenomeNet (http://www.genome.jp) websites for pathway mapping and GO analysis based on the KEGG/GO enrichment scores.

The resulted transcriptome annotation information was compared to available transcriptome information from *C. reinhardtii* v5.5 (https://phytozome.jgi.doe.gov/pz/portal.html#!info?alias=Org_Creinhardtii), *V. carteri* v2.1 (https://phytozome.jgi.doe.gov/pz/portal.html#!info?alias=Org_Vcarteri), *D. salina* v1.0 (https://phytozome.jgi.doe.gov/pz/portal.html#!info?alias=Org_Dsalina_er).

### Differential gene expression analysis

We used Nr transcript dataset constructed as the reference for mapping the sequencing reads using RSEM version 1.2.29 with default settings [27], and subsequently imported and normalized in EBSeq for gene differential expression analysis [28]. Deferentially transcribed contigs upon ND is obtained using a cutoff of fold change (post) > = 2 or < = -2, and PPEE (FDR) value < = 0.05.

### Retrieval of redundant contigs

Normally, Nr transcripts after BLASTX best-hit search were selected for transcriptome database construction. There are four basic types of non-top-hit events: 1) false *de novo* assembly; 2) isoforms with alternative transcription starts or ends; 3) breakage of an integrated gene; 4) alternative splicing (exon skipping, intron retention, alternative 5′ or 3′ splices site, mutually exclusive exons) [29–31]. Python scripts using ClustalW algorithm [32] were proposed to check the similarities of all the redundant contigs, which hit the same ncbi_proteinID. Alternative-splicing variants from *A. thaliana*, *C. reinhardtii*, *V. carteri*, *D. salina* were extracted and their homogeneities were compared.

## Results and discussion

### System environment for experimental software

**Fig. 1:**
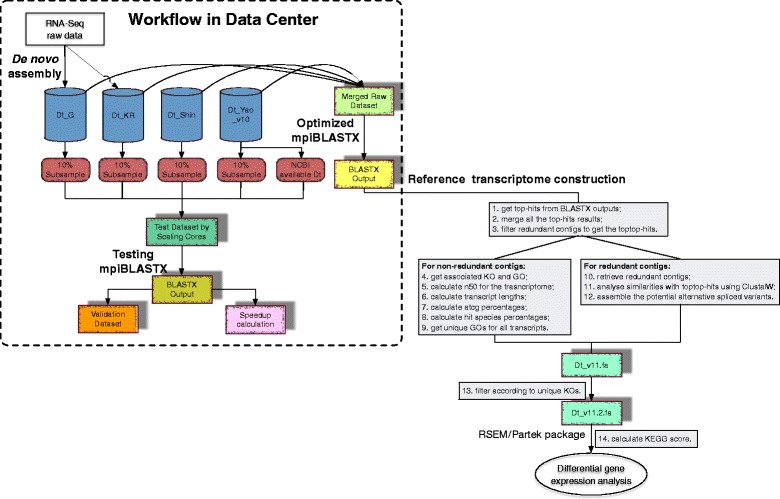
Pipeline of RNA-Seq data analysis workflow from short sequence raw data. The general pipeline includes workflow for transcriptome database construction, annotation, and differential gene expression analysis. Workflow in data center mainly consists of *de novo* assembly, mpiBLASTX. Dt_G and Dt_KR are in-house constructed samples. Dt_Shin and Dt_Yao_v10 are two published datasets. 10% of each datasets are randomly picked for mpiBLASTX test to get the best parameters for mpiBLASTX with NCBI database for the merged *D. tertiolecta* datasets. The mpiBLASTX output was further extracted and filtered for annotation using python scripts

In general, the complete workflow follows Fig. 1. Experiments using HPC were completed on the petascale National Supercomputing Centre (NSCC), which comprises of 1288 nodes (dual socket, 12 cores/CPU E5-2690v3), 128 GB DDR4/ node. Additionally, 9 nodes are equipped with more than 1 TB memory RAM for enabling large memory applications. All the software settings used for construction and analyses of the transcriptome in this study are described in Additional file 1.

**Fig. 2:**
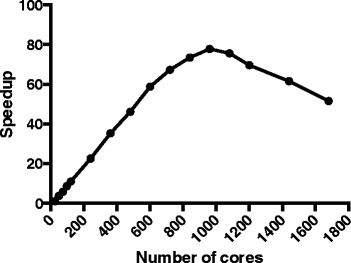
Speedup achieved by mpiBLASTX calculated over run of 24 cores. It shows the scalability test based on the subsampling from the four sources of datasets, where we increased the number of cores in the system for mpiBLASTX application from 24 (1 node) to 1680 (70 nodes) cores and measured the speedup achieved. It was concluded that using 960 (40 nodes) cores was optimal regarding the time cost in this study

It is reported that the conventional BLASTX is computationally intensive and embarrassingly parallel [33]. As the input high-throughput data size continuously increases, time cost becomes the major issue. An open-source parallelization of BLASTX (mpiBLASTX version 1.6.0), that segments and distributes a BLASTX database among cluster nodes such that each node searches a unique portion of the database was a great advantage for speedup than the conventional single-core BLASTX. Thus, instead of using normal single-core BLASTX, we used mpiBLASTX. In mpiBLASTX, database needs to be segmented into 24 fragments prior to do BLAST by using mpiformatdb [33]. The database segmentation can save time from producing heavy intercommunication between nodes to realize the elimination of high overhead of disk I/O [33]. Figure 2 shows the scalability test based on the subsampling from the four sources of datasets, where we increased the number of cores in the system for mpiBLASTX application from 24 (1 node) to 1680 (70 nodes) cores and measured the speedup achieved. It was concluded that using 960 (40 nodes) cores was optimal regarding the time cost in this study. The performance flourishes when increasing core count from 24 to 960, as a result of the abundant parallelism. For configurations with more than 960 cores, however, the performance begins to diminish because the communication cost becomes the predominant factor, rendering the computing cores underutilized. Therefore, prompted by the subsampling results, the optimal configuration was used in our real study.

### *De novo* assembly of *D. tertiolecta* transcriptome

Strand-specific RNA sequencing data from each condition were pooled together for *de novo* transcriptome assembly of *D. tertiolecta* transcriptome database and subsequent gene expression analysis. Due to the unavailability of complete genome or transcriptome information of *D. tertiolecta* LB 999, the *de-novo* assembled RNA-seq data can be used to identify genes. In particular, bigger input pooled data could enlarge the output transcriptome. To evaluate the transcripts created from different input datasets, assembly statistics between different raw data were compared and pooled together (Table 1). As a result, 87,197 contigs were merged for annotation.

### Annotation of de novo-assembled transcriptome

Using the *de novo*-assembled contigs, annotation based on BLASTX with plant and bacterial Nr protein database was performed, to obtain protein identification from the taxonomy of plant and bacteria. *De novo* assembly methods are known to produce false positive contigs proportional to the sequencing depth [34]. Among the total 87,197 contigs obtained from the pooled library, only 17,845 transcripts were matched to proteins falling into our criteria to remove any false positives. The 17,845 annotated *D. tertiolecta* transcripts were subjected to functional analysis. Transcript length was ranged from 114 bps to 16,518 bps.

Of the 17,845 transcripts, 2525 are associated with at least one GO function, and 10,790 were found to have KEGG gene name and 5227 associated KO through python based scripts from online KEGG database. However, some transcripts with the different KEGG gene name would end into the same KO. For the unique pathway analysis, we further filtered the transcripts that have the same KO by the following criteria: 1) filter out the same KO as a category; 2) accelerating E-value; 2) decelerating sequence length. As a result, 15,336 transcripts (regarded as Dt_v11.2) were generated. Subsequently, 2718 out of the 15,336 transcripts have unique KO and associated with at least one metabolic pathway.

### Analysis of the *D. tertiolecta* transcriptome information

**Fig. 3:**
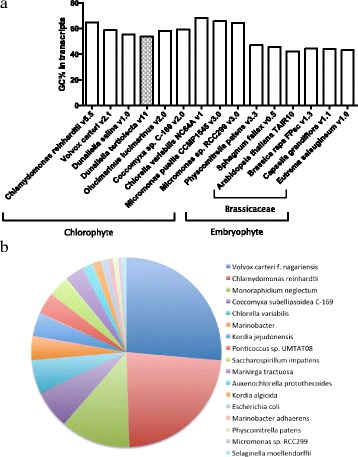
*D. tertiolecta* transcriptome information. **a** GC content in *D. tertiolecta* transcriptome compared with other species; **b** Identification and verification of the protein-coding transcripts in *D. tertiolecta*. This pie chart is the result from BLASTX output. The sum of top hit transcripts from each individual species. The right side species names: descending numbers of hits

GC content is an indicator for many features of an organism, and it is correlated with various genomic features, including repeat element distribution, methylation pattern, and gene density [35–38]. The transcriptome of *D. tertiolecta* (54%) and *D. salina* (55%) showed higher GC content than higher plants studied here, but lowest GC content among the microalgal species and even lower than moss species (Fig. 3a). This phenomenon may reveal new insights into the gene regulatory mechanisms required for evolution among *Viridiplantae*, or green plants [39] according to the ancestor nodes from Phytozome database (https://phytozome.jgi.doe.gov/pz/portal.html#!search).

We also looked into the best-hit annotation models in the BLASTX search. Majority (73.7%) of the best-hit annotations in *D. tertiolecta* were found to derive from green algal species (Fig. 3b). *D. tertiolecta* transcriptome appears to mostly resemble that of *V. carteri* (26.4%), followed by *C. reinhardtii* (23.1%), *Monoraphidium neglectum* (11.9%), *C. subellipsoidea* C-169 (6.6%), and *C. variabilis* (5.7%) (Fig. 3b).

Furthermore, approximately 97.2% of the core eukaryotic genes (CEGs) were found in the 17,845 transcripts, suggesting a rather high coverage of transcripts that has been obtained to represent the *D. tertiolecta* LB 999 transcriptome [40]. A detailed comparison of transcriptome information of *D. tertiolecta* with other species is presented in Table 2. This has shown that the newly constructed database (Dt_v11.2) has a relatively good coverage (~95% that of *D. salina*) of transcripts and high assembly and annotation quality. N50, maximum contig length, total size of contigs, number of protein-coding transcripts, and average contig length all increased based on the current available *D. tertiolecta* databases. To this end, this enhanced transcriptome database comprising core genes in *D. tertiolecta* was used as a reference for following studies.

**Table 2:** Transcriptome assembly and annotation descriptions of different species

	*C. reinhardtii* v5.5	*V. carteri* v2.1	*D. salina* v1.0	*D. tertiolecta* Shin et al.	Dt_v11*/Dt_v11.2*
Genome description	111.1 Mb arranged on 17 chromosomes and 37 minor scaffolds	131.2 Mb arranged in 434 scaffolds	343.7 Mb arranged in 5512 scaffolds	-	-
N50 (bp)	3938	4188	2291	1540	1797
Maximum contig length (bp)	72,700	24,197	17,353	15,234	16,518
Total size of contigs (bp)	63,797,006	51,775,597	33,246,103	16,600,538	24,538,468
Protein-coding transcripts	19,526	16,075	18,801	13,861	-
transcript_primaryTranscriptOnly	17,741	14,247	16,697	9839	17,845 */ 15,336*
Average length (bp)	3267	3220	1768	1197	1375
Alternatively spliced transcripts	1785	1828	2104	-	-

The ﻿entry in italic represents data from Dt_v11.2

Microalgae are a highly diverse group with largely unexplored genetic information, and there was the enormous amount (67.7% distinct) of diversity among microalgae at genetic level, which indicated that the functional genetic information is very diverse and case-dependent in microalgae, though they could be morphologically similar [41]. Therefore, the enrichment of *D. tertiolecta* transcriptome database is a necessity for accurate genetic engineering and RNA-Seq analysis, using larger input data, and multiple annotation species.

Construction of transcriptome coverage can vary due to expression differences and input data depth [42]. Theoretically, when addition input reads does not provide new output information, a sequencing saturation depth was hit. Several studies suggested that saturation depths at 95% gene coverage [43–46]. However, in this study, the use of the increasing number of high-throughput sequencing data enlarged the *de novo* transcriptome assembly to ~95% of *Dunaliella* genes. The enhancement and exploration of the database gave us essential and additional information for comparative analysis of the transcriptome data.

Alternative splicing, an essential mechanism for increasing transcriptome and proteome diversity in eukaryotes, are quite common [39]. It is however less clear, and has few reports in microalgae. Venn diagram in Additional file 2 shows that the alternative-splicing among different species has little homogeneity. The retrieved *D. tertiolecta* potential splicing variants appear to be diverse and do not resemble much of those in the close related species. Further, different GC content (Fig. 3a) might also cause differences between the species in alternative splicing as reported elsewhere [47, 48]. To further verify the predicted alternative-splicing variants, genome sequencing or third generation sequencing (single-molecule long-read sequencing) is necessary.

### Case study of RNA-Seq data from nitrogen-deprived cells

*D. tertiolecta* ND cells were chosen as a case study for comparing results using RSEM-EbSeq pipeline and Partek software, as the transcriptomic and physiological responses are well documented in microalgae to promote TAG accumulation [49]. We found that nitrogen-deprived *D. tertiolecta* cells on culture day 5 had comparable DCW but remarkably increase in TAG. It was reported carbohydrate accumulation during the early stages of ND conditions existed, which could account for its little increase in DCW ([15, 50]).

**Fig. 4:**
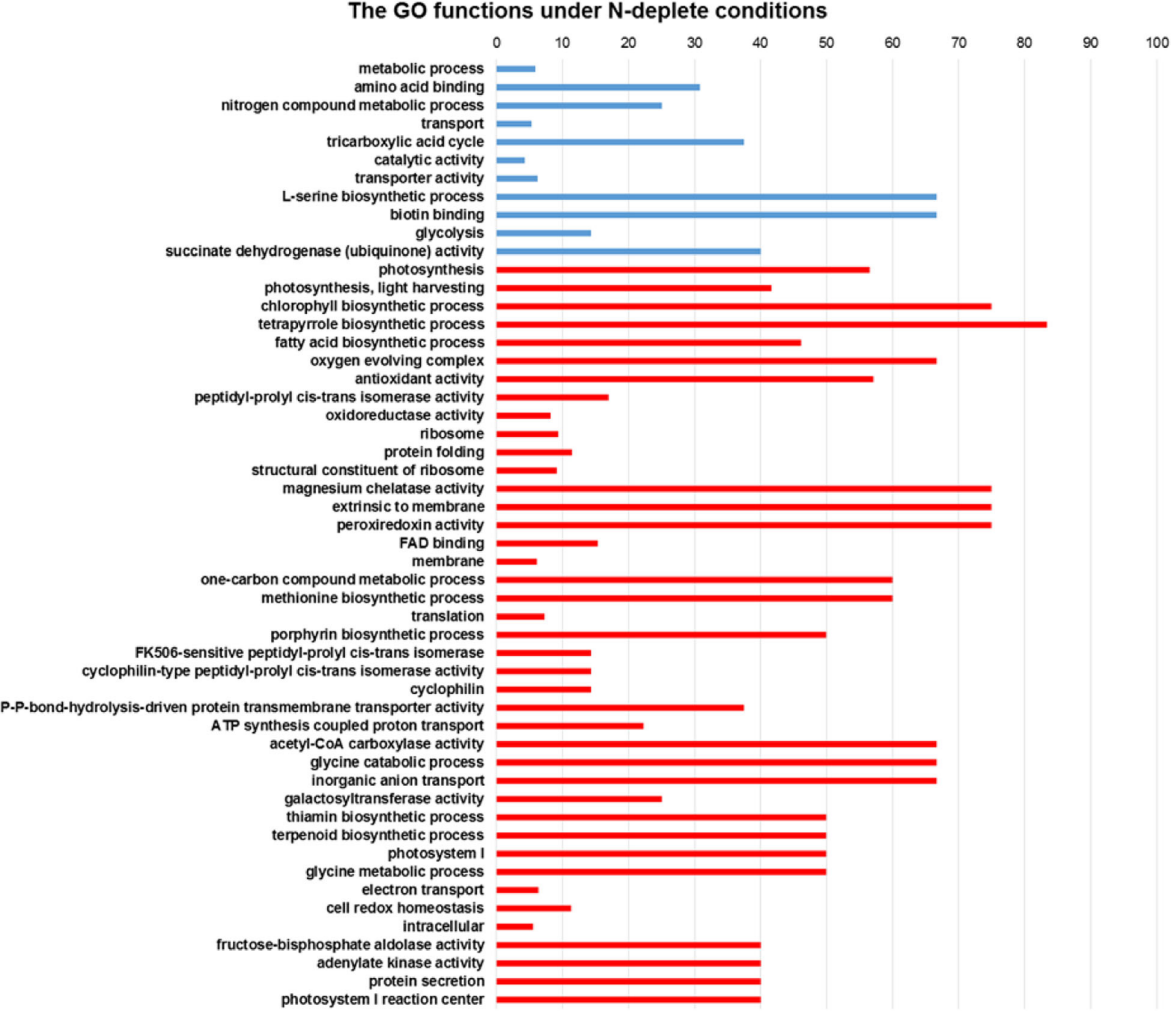
GO functional enrichment of up-regulated (blue) and down-regulated (red) genes under ND conditions. Categories were filtered by Fisher’s exact test with an FDR-corrected *p*-value ≤ 0

Through Illumina MISEQ sequencing, over 27 million qualified raw reads with 150 bps in length were used. Besides contributing to our large database construction, these data were analyzed for differential gene expression. Raw data were deposited in SRA database (SRR4011625, SRR4011626, SRR4011627, SRR4011628). Using Partek pipelines based on *C. reinhardtii* annotation [16], differential gene expression, significant GO output were presented in Additional file 3a and Fig. 4, respectively. From the perspective of significant differential expressed genes, the number increased from 482 to 582 after updated to Dt_v11 analysis (Additional file 3a-b). The detailed KEGG and GO lists are presented in Additional file 4a and Additional file 5, respectively. Most GO families showed repressed under ND conditions.

Equipped with more genes annotated from different plant and bacterial species of the newly constructed database, we reported results of differential gene expression with top fold changes from RSEM and Partek pipelines (Additional file 3b). Based on the Dt_v11 GO annotation, a.gmt file requested by biological analysis with unique GO reflecting to different transcripts.

Regarding KO, given one culture condition a, and one KEGG term KEGG_b_, the KEGG enrichment score in the network x can be computed by the below equation (Eq.1) [51–54]:

$$ \begin{array}{c}\hfill {S}_{G O/ KEGG}\left( a, KEG{G}_b\right)=- lo{g}_{10}\left( p- value\right)\hfill \\ {}\hfill =- lo{g}_{10}\left({\displaystyle \sum_{k= m}^n}\frac{\left(\begin{array}{c}\hfill M\hfill \\ {}\hfill m\hfill \end{array}\right)\left(\begin{array}{c}\hfill N- M\hfill \\ {}\hfill n- m\hfill \end{array}\right)}{\left(\begin{array}{c}\hfill N\hfill \\ {}\hfill n\hfill \end{array}\right)}\right)\hfill \end{array} $$

where N, is the total number of genes with KEGG in a certain number of species (x), M, is the number of proteins that are annotated to the KEGG term KEGG_b_, n, is the number of proteins in K(a), and m is the number of proteins both in K(a) and annotated to the KEGG term KEGG_b_, respectively. Larger the enrichment score of one KEGG term is, more overrepresented such a term is under the culture condition a [55, 56].

In this study, the annotation was performed based on all plants and bacterial protein database; this x is not easily adopted, therefore we could still compare the enrichment score according to the relative values. Through in-house constructed scripts to calculate KEGG enrichment score, the output of significant KEGG pathways was presented in Additional file 4b. Combined with Dt_v10 (MISEQ) [16] as the baseline, we found additional KEGG pathways that were significantly representative in the ND treatment with change of gene expression levels on the basis of a larger annotation. These included nitrogen metabolism, fatty acid biosynthesis, fatty acid metabolism, biotin metabolism, terpenoid backbone biosynthesis, propanoate metabolism, pentose phosphate pathway, oxidative phosphorylation, 2-oxocarboxylic acid metabolism, DNA replication, insulin resistance, starch and sucrose metabolism. Genes participating in fatty acid biosynthesis/metabolism and nitrogen metabolism pathway, which were detected in Dt_v11 analysis but not in Dt_v10 analysis, were summarized in Table 3. Integration of significant genes hit in KEGG biological pathway analysis from Dt_v10 and Dt_v11, KEGG maps were constructed as shown in Additional file 6a-e. Using the ND study, pathway maps showing the well annotated and enriched transcriptome database help shed light on metabolic pathways and regulation of *D. tertiolecta* and guide rational synthetic biology approaches.

**Table 3:** Comparison of dry cell weight and TAG content in *D. tertiolecta* ND culture

	Dry cell weight (g/L)	TAG content (pg/cell)	Fatty acid content (% DCW)
N-replete	0.31 ± 0.08	0.15 ± 0.02	6.2 ± 0.27
N-deplete	0.34 ± 0.04	1.29 ± 0.12	5.14 ± 0.45

The values are presented as the mean ± the standard deviation

### Global gene expression level change under ND conditions

In this ND study, we observed all the photosynthetic genes were repressed (Additional file 6a-b). Consistent with this, Yang et al. observed the similar trend in *Phaeodactylum tricurnutum*, and extensive degradation of chloroplast membranes under an electron microscope, with photosynthetic efficiency declined [57]. It suggested that nitrogen is an essential factor for photosynthetic activity. As nitrogen is an important component for the synthesis of chlorophyll and photosystem proteins (e.g. light-harvesting complex II, LHCII apoprotein), the reduction in nitrogen availability could hinder the expression of genes related to photosynthesis. Indeed, chlorophyll content and PSII quantum yield were decreased in *D. tertiolecta* upon ND conditions [58] which corresponds with the decreased gene expression. Multiple studies of nutrient deprivation in microalgae suggest that degradation of thylakoid membranes is responsible for the downregulation of most LHC genes [57, 59–61], indicating that ND triggered a cellular response for reorganization of photosynthetic apparatus.

Genes in fatty acid biosynthesis and metabolism were also found to be repressed, while TAG levels significantly increased, indicating that intracellular membrane remodeling might have substantially contributed to the neutral lipid accumulation, instead of *de novo* lipid synthesis. It is consistent with Table 4, where FA percentage of the nitrogen-deprived cells was comparable and even a little decrease than that of the control. This is similar to observations by other studies which saw declines in total FA content under ND conditions [15]. The parallel increase in TAG coupled with a decline in total FA could be explained by the degradation of thylakoid membranes and rechanneling of carbon towards storage compounds such as TAGs. A recent report showed that when *D. tertiolecta* is cultured under ND condition, it had significant decreases in the lipid classes diacylglyceryltrimethylhomoserine (DGTS) and digalactosyldiacylglycerol (DGDG), a main component of chloroplast membranes [62], suggesting that a major remodeling of lipid membranes has occurred after ND.

**Table 4:** Genes participating in important pathways that are exclusively found in Dt_v11

KO	Name	Definition	Fold Change (N-deplete/ N-replete)
ko00061 Fatty acid biosynthesis & ko01212 Fatty acid metabolism			
ko:K00059	fabG	3-oxoacyl-[acyl-carrier protein] reductase [EC:1.1.1.100]	-3.100679049
ko:K00208	fabI	enoyl-[acyl-carrier protein] reductase I [EC:1.3.1.9 1.3.1.10]	-3.38232061
ko:K00645	fabD	[acyl-carrier-protein] S-malonyltransferase [EC:2.3.1.39]	-3.52911698
ko:K01962	accA	acetyl-CoA carboxylase carboxyl transferase subunit alpha [EC:6.4.1.2]	-2.126940035
ko:K01963	accD	acetyl-CoA carboxylase carboxyl transferase subunit beta [EC:6.4.1.2]	-3.075257707
ko:K02160	accB	acetyl-CoA carboxylase biotin carboxyl carrier protein	-7.80359902
ko:K02372	fabZ	3-hydroxyacyl-[acyl-carrier-protein] dehydratase [EC:4.2.1.59]	-2.198560822
ko:K09458	fabF	3-oxoacyl-[acyl-carrier-protein] synthase II [EC:2.3.1.179]	-3.332311424
ko00910 Nitrogen metabolism			
ko:K00264	GLT1	glutamate synthase (NADPH/NADH) [EC:1.4.1.13 1.4.1.14]	23.8846
ko:K00366	nirA	ferredoxin-nitrite reductase [EC:1.7.7.1]	-13.89942929
ko:K01915	glnA	glutamine synthetase [EC:6.3.1.2]	2.84411
ko:K02575	NRT	MFS transporter, NNP family, nitrate/nitrite transporter	-4.415225463
ko:K10534	NR	nitrate reductase (NAD(P)H) [EC:1.7.1.1 1.7.1.2 1.7.1.3]	-9.464319515

Interestingly, from the nitrogen metabolism, we found the glutamate synthase (NADPH/NADH) was greatly activated under ND conditions. Similarly, Shin et al. also observed similar results, proving that intracellular glutamate levels were the main factor for the regulation of cell growth and carbon accumulation [15]. Further studies on metabolite profiling of glutamate and related amino acid levels could be potential targets to uncover the regulatory mechanism more specifically. As a signal of the growth delimitation under ND, genetic engineering to activate or block certain enzyme coding genes would promote the biofuel-relevant productions in microalgae.

## Conclusions

In this study, fueled by a high performance data center (NSCC), high quality high-throughput RNA-Seq data were *de novo* assembled and annotated, which resulted in 17,845 protein-coding transcripts in *D. tertiolecta*. Integration of paralleled assembly and annotation method under HPC presented here enables large-scale data processing in a reasonable computation time. Ultimately, a significant increase of transcriptomic data (~95% of the total genes in *Dunaliella*) was achieved. The enhanced transcriptomic database subjected to the analysis of RNA-Seq data from ND study gave us a new insight of regulation mechanisms of cell growth and lipid biosynthesis, suggesting that the increase of TAGs were mainly derived from internal bioconversion to improve lipid production concurrently with exponential cell growth. Overall, these results laid the foundation for demonstrating integration of supercomputing with large input datasets to improve microalgal transcriptomic database and elucidate the regulatory response of ND conditions for promoting biofuel production. Further, this pipeline written and packaged by python scripts facilitates its use by non-experts. We believe that the uncovered transcriptomic database can play a key role in the development of this microalga for biofuel use and some related models with high-throughput raw data.

## Additional files

Additional file 1: Detailed steps of transcriptome construction and analyses. (a) Trinity (v2.2.0) for assembly of transcriptome; (b) mpiBLASTX (v1.6.0) for comparison of homologous sequences; (c) Dt_v11.fa and Dt_v11.2.fa transcript files and their annotation information were generated via optimized Bag2D to filter the redundant contigs and get the top-hits within the criteria; (d) RSEM (v1.2.29) for mapping the sequencing reads. (DOCX 104 kb)
Additional file 2: Venn diagram of the numbers of *D. tertiolecta* transcripts with BLASTX hits of alternative-splicing variants from four organisms. A, *A. thaliana*; B, *C. reinhardtii*; C, *V. carteri*; E, *D. salina*. (DOCX 132 kb)
Additional file 3: List of differential expression genes in nitrogen-deprived *D. tertiolecta* cells in Dt_v10 and Dt_v11 analyses. (a) Dt_v10 analysis; (b) Dt_v11 analysis. (DOCX 401 kb)
Additional file 4: KEGG analyses from Dt_v10 and Dt_v11. (a) Dt_v10 analysis; (b) Dt_v11 analysis. (DOCX 65 kb)
Additional file 5: GO analysis list. (DOCX 92 kb)
Additional file 6: Integration of significant genes hit in KEGG biological pathway analysis from Dt_v10 and Dt_v11. With red color boxes mean upregulation, and green mean downregulation. The value in the bracket means the fold change of gene expression level in the study of ND/replete while using either v10 or v11 database. (a) Photosynthesis; (b) Photosynthesis - Antenna proteins; (c) Citrate cycle (TCA cycle); (d) Pyruvate metabolism; (e) Glycolysis/Gluconeogenesis. (DOCX 987 kb)

## Abbreviations

BLAST: Basic Local Alignment Search Tool
DCW: Dry cell weight
GO: Gene Ontology
HPC: High performance computing
KEGG: Kyoto Encyclopedia of Genes and Genomes
KO: KEGG Orthology
ND: Nitrogen deprivation
NGS: Next-generation sequencing
Nr: Non-redundant
NSCC: National Supercomputing Centre
TAG: Triacylglycerol

## References

[1] Wang Z, Gerstein M, Snyder M (2009). RNA-Seq: a revolutionary tool for transcriptomics. Nat Rev Genet.

[2] Chang Z, Li G, Liu J, Zhang Y, Ashby C, Liu D, Cramer CL, Huang X (2015). Bridger: a new framework for *de novo* transcriptome assembly using RNA-seq data. Genome Biol.

[3] Grabherr MG, Haas BJ, Yassour M, Levin JZ, Thompson DA, Amit I, Adiconis X, Fan L, Raychowdhury R, Zeng Q (2011). Full-length transcriptome assembly from RNA-Seq data without a reference genome. Nat Biotechnol.

[4] Martin JA, Wang Z (2011). Next-generation transcriptome assembly. Nat Rev Genet.

[5] Blanc G, Agarkova I, Grimwood J, Kuo A, Brueggeman A, Dunigan DD, Gurnon J, Ladunga I, Lindquist E, Lucas S (2012). The genome of the polar eukaryotic microalga *Coccomyxa subellipsoidea* reveals traits of cold adaptation. Genome Biol.

[6] Blanc G, Duncan G, Agarkova I, Borodovsky M, Gurnon J, Kuo A, Lindquist E, Lucas S, Pangilinan J, Polle J (2010). The *Chlorella variabilis* NC64A genome reveals adaptation to photosymbiosis, coevolution with viruses, and cryptic sex. Plant Cell Online.

[7] Merchant SS, Prochnik SE, Vallon O, Harris EH, Karpowicz SJ, Witman GB, Terry A, Salamov A, Fritz-Laylin LK, Maréchal-Drouard L (2007). The *Chlamydomonas* genome reveals the evolution of key animal and plant functions. Science.

[8] Worden AZ, Lee J-H, Mock T, Rouzé P, Simmons MP, Aerts AL, Allen AE, Cuvelier ML, Derelle E, Everett MV (2009). Green evolution and dynamic adaptations revealed by genomes of the marine picoeukaryotes *Micromonas*. Science.

[9] Palenik B, Grimwood J, Aerts A, Rouzé P, Salamov A, Putnam N, Dupont C, Jorgensen R, Derelle E, Rombauts S (2007). The tiny eukaryote *Ostreococcus* provides genomic insights into the paradox of plankton speciation. Proc Natl Acad Sci.

[10] Prochnik SE, Umen J, Nedelcu AM, Hallmann A, Miller SM, Nishii I, Ferris P, Kuo A, Mitros T, Fritz-Laylin LK (2010). Genomic analysis of organismal complexity in the multicellular green alga *Volvox carteri*. Science.

[11] Shang C, Bi G, Yuan Z, Wang Z, Alam MA, Xie J (2016). Discovery of genes for production of biofuels through transcriptome sequencing of *Dunaliella parva*. Algal Res.

[12] Fang L, Sun D, Xu Z, He J, Qi S, Chen X, Chew W, Liu J (2015). Transcriptomic analysis of a moderately growing subisolate *Botryococcus braunii* 779 (*Chlorophyta*) in response to nitrogen deprivation. Biotechnol Biofuels.

[13] Rismani-Yazdi H, Haznedaroglu BZ, Bibby K, Peccia J (2011). Transcriptome sequencing and annotation of the microalgae *Dunaliella tertiolecta*: pathway description and gene discovery for production of next-generation biofuels. BMC Genomics.

[14] Rismani-Yazdi H, Haznedaroglu BZ, Hsin C, Peccia J (2012). Transcriptomic analysis of the oleaginous microalga *Neochloris oleoabundans* reveals metabolic insights into triacylglyceride accumulation. Biotechnol Biofuels.

[15] Shin H, Hong S-J, Kim H, Yoo C, Lee H, Choi H-K, Lee C-G, Cho B-K (2015). Elucidation of the growth delimitation of *Dunaliella tertiolecta* under nitrogen stress by integrating transcriptome and peptidome analysis. Bioresour Technol.

[16] Yao L, Tan TW, Ng Y-K, Ban KHK, Shen H, Lin H, Lee YK (2015). RNA-Seq transcriptomic analysis with Bag2D software identifies key pathways enhancing lipid yield in a high lipid-producing mutant of the non-model green alga *Dunaliella tertiolecta*. Biotechnol Biofuels.

[17] Zerbino DR, Birney E (2008). Velvet: algorithms for *de novo* short read assembly using de Bruijn graphs. Genome Res.

[18] Birol I, Jackman SD, Nielsen CB, Qian JQ, Varhol R, Stazyk G, Morin RD, Zhao Y, Hirst M, Schein JE (2009). *De novo* transcriptome assembly with ABySS. Bioinformatics.

[19] Li R, Yu C, Li Y, Lam T-W, Yiu S-M, Kristiansen K, Wang J (2009). SOAP2: an improved ultrafast tool for short read alignment. Bioinformatics.

[20] Haas BJ, Papanicolaou A, Yassour M, Grabherr M, Blood PD, Bowden J, Couger MB, Eccles D, Li B, Lieber M (2013). *De novo* transcript sequence reconstruction from RNA-seq using the Trinity platform for reference generation and analysis. Nat Protoc.

[21] Henschel R, Lieber M, Wu L-S, Nista PM, Haas BJ, LeDuc RD. Trinity RNA-Seq assembler performance optimization. XSEDE '12 Proceedings of the 1st Conference of the Extreme Science and Engineering Discovery Environment. Chicago: bridging from the eXtreme to the campus and beyond. 2012. http://dx.doi.org/10.1145/2335755.2335842.

[22] Corwin J, Köhler S, Zerick J. A virtual machine program-suite for distributed *de novo* genome construction and motif finding. 2013. http://www.blueideas.de/ecs234_s10_cloudcomputing.pdf.

[23] Peréz-Sánchez H, Cecilia JM, Merelli I. The role of high performance computing in bioinformatics. 2014. http://iwbbio.ugr.es/2014/papers/IWBBIO_2014_paper_57.pdf.

[24] Lee AF, Bennett JA, Manayil JC, Wilson K (2014). Heterogeneous catalysis for sustainable biodiesel production via esterification and transesterification. Chem Soc Rev.

[25] Cavonius LR, Carlsson N-G, Undeland I (2014). Quantification of total fatty acids in microalgae: comparison of extraction and transesterification methods. Anal Bioanal Chem.

[26] Laurens LM, Quinn M, Van Wychen S, Templeton DW, Wolfrum EJ (2012). Accurate and reliable quantification of total microalgal fuel potential as fatty acid methyl esters by in situ transesterification. Anal Bioanal Chem.

[27] Li B, Dewey CN (2011). RSEM: accurate transcript quantification from RNA-Seq data with or without a reference genome. BMC Bioinformatics.

[28] Leng N, Dawson JA, Thomson JA, Ruotti V, Rissman AI, Smits BMG, Haag JD, Gould MN, Stewart RM, Kendziorski C. EBSeq: an empirical Bayes hierarchical model for inference in RNA-seq experiments. Bioinformatics. 2013;29:1035–043. 10.1093/bioinformatics/btt087 PMC3624807 23428641

[29] Black DL (2003). Mechanisms of alternative pre-messenger RNA splicing. Annu Rev Biochem.

[30] Reddy AS (2007). Alternative splicing of pre-messenger RNAs in plants in the genomic era. Annu Rev Plant Biol.

[31] Stamm S, Ben-Ari S, Rafalska I, Tang Y, Zhang Z, Toiber D, Thanaraj T, Soreq H (2005). Function of alternative splicing. Gene.

[32] Thompson JD, Gibson T, Higgins DG: Multiple sequence alignment using ClustalW and ClustalX. Curr Protoc Bioinformatics. 2002:2.3. 1-2.3. 22. 10.1002/0471250953.bi0203s00 18792934

[33] Darling A, Carey L, Feng W-C (2003). The design, implementation, and evaluation of mpiBLAST. Proceed ClusterWorld.

[34] Sims D, Sudbery I, Ilott NE, Heger A, Ponting CP (2014). Sequencing depth and coverage: key considerations in genomic analyses. Nat Rev Genet.

[35] Duret L, Mouchiroud D, Gautier C (1995). Statistical analysis of vertebrate sequences reveals that long genes are scarce in GC-rich isochores. J Mol Evol.

[36] Galtier N, Piganeau G, Mouchiroud D, Duret L (2001). GC-content evolution in mammalian genomes: the biased gene conversion hypothesis. Genetics.

[37] Jabbari K, Bernardi G (1998). CpG doublets, CpG islands and Alu repeats in long human DNA sequences from different isochore families. Gene.

[38] Mouchiroud D, D’Onofrio G, Aissani B, Macaya G, Gautier C, Bernardi G (1991). The distribution of genes in the human genome. Gene.

[39] Kianianmomeni A, Ong CS, Rätsch G, Hallmann A (2014). Genome-wide analysis of alternative splicing in *Volvox carteri*. BMC Genomics.

[40] Parra G, Bradnam K, Ning Z, Keane T, Korf I (2009). Assessing the gene space in draft genomes. Nucleic Acids Res.

[41] Gimpel JA, Specht EA, Georgianna DR, Mayfield SP (2013). Advances in microalgae engineering and synthetic biology applications for biofuel production. Curr Opin Chem Biol.

[42] Bullard JH, Purdom E, Hansen KD, Dudoit S (2010). Evaluation of statistical methods for normalization and differential expression in mRNA-Seq experiments. BMC Bioinformatics.

[43] Blencowe BJ, Ahmad S, Lee LJ (2009). Current-generation high-throughput sequencing: deepening insights into mammalian transcriptomes. Genes Dev.

[44] Cloonan N, Forrest AR, Kolle G, Gardiner BB, Faulkner GJ, Brown MK, Taylor DF, Steptoe AL, Wani S, Bethel G (2008). Stem cell transcriptome profiling via massive-scale mRNA sequencing. Nat Methods.

[45] Francis WR, Christianson LM, Kiko R, Powers ML, Shaner NC, Haddock SH (2013). A comparison across non-model animals suggests an optimal sequencing depth for *de novo* transcriptome assembly. BMC Genomics.

[46] Li H, Lovci MT, Kwon Y-S, Rosenfeld MG, Fu X-D, Yeo GW (2008). Determination of tag density required for digital transcriptome analysis: application to an androgen-sensitive prostate cancer model. Proc Natl Acad Sci.

[47] Goodall GJ, Filipowicz W (1991). Different effects of intron nucleotide composition and secondary structure on pre-mRNA splicing in monocot and dicot plants. EMBO J.

[48] White O, Soderlund C, Shanmugan P, Fields C (1992). Information contents and dinucleotide compositions of plant intron sequences vary with evolutionary origin. Plant Mol Biol.

[49] de Lomana ALG, Schäuble S, Valenzuela J, Imam S, Carter W, Bilgin DD, Yohn CB, Turkarslan S, Reiss DJ, Orellana MV (2015). Transcriptional program for nitrogen starvation-induced lipid accumulation in *Chlamydomonas reinhardtii*. Biotechnol Biofuels.

[50] Siaut M, Cuiné S, Cagnon C, Fessler B, Nguyen M, Carrier P, Beyly A, Beisson F, Triantaphylidès C, Li-Beisson Y (2011). Oil accumulation in the model green alga *Chlamydomonas reinhardtii*: characterization, variability between common laboratory strains and relationship with starch reserves. BMC Biotechnol.

[51] Carmona-Saez P, Chagoyen M, Tirado F, Carazo JM, Pascual-Montano A (2007). GENECODIS: a web-based tool for finding significant concurrent annotations in gene lists. Genome Biol.

[52] Huang T, Shi X-H, Wang P, He Z, Feng K-Y, Hu L, Kong X, Li Y-X, Cai Y-D, Chou K-C (2010). Analysis and prediction of the metabolic stability of proteins based on their sequential features, subcellular locations and interaction networks. PLoS One.

[53] Huang T, Wan S, Xu Z, Zheng Y, Feng K-Y, Li H-P, Kong X, Cai Y-D (2011). Analysis and prediction of translation rate based on sequence and functional features of the mRNA. PLoS One.

[54] Huang T, Wang P, Ye Z-Q, Xu H, He Z, Feng K-Y, Hu L, Cui W, Wang K, Dong X (2010). Prediction of deleterious non-synonymous SNPs based on protein interaction network and hybrid properties. PLoS One.

[55] Chen L, Chu C, Lu J, Kong X, Huang T, Cai Y-D (2015). Gene ontology and KEGG pathway enrichment analysis of a drug target-based classification system. PLoS One.

[56] Huang T, Zhang J, Xu Z-P, Hu L-L, Chen L, Shao J-L, Zhang L, Kong X-Y, Cai Y-D, Chou K-C (2012). Deciphering the effects of gene deletion on yeast longevity using network and machine learning approaches. Biochimie.

[57] Yang Z-K, Niu Y-F, Ma Y-H, Xue J, Zhang M-H, Yang W-D, Liu J-S, Lu S-H, Guan Y, Li H-Y (2013). Molecular and cellular mechanisms of neutral lipid accumulation in diatom following nitrogen deprivation. Biotechnol Biofuels.

[58] Tan KWM, Lin H, Shen H, Lee YK. Nitrogen-induced metabolic changes and molecular determinants of carbon allocation in *Dunaliella tertiolecta*. Scientific Reports. 2016;6:37235 10.1038/srep37235 PMC5110973 27849022

[59] Martin GJ, Hill DR, Olmstead IL, Bergamin A, Shears MJ, Dias DA, Kentish SE, Scales PJ, Botté CY, Callahan DL (2014). Lipid profile remodeling in response to nitrogen deprivation in the microalgae *Chlorella* sp.(Trebouxiophyceae) and *Nannochloropsis* sp.(Eustigmatophyceae). PLoS One.

[60] Simionato D, Block MA, La Rocca N, Jouhet J, Maréchal E, Finazzi G, Morosinotto T (2013). The response of *Nannochloropsis gaditana* to nitrogen starvation includes *de novo* biosynthesis of triacylglycerols, a decrease of chloroplast galactolipids, and reorganization of the photosynthetic apparatus. Eukaryot Cell.

[61] Urzica EI, Vieler A, Hong-Hermesdorf A, Page MD, Casero D, Gallaher SD, Kropat J, Pellegrini M, Benning C, Merchant SS (2013). Remodeling of membrane lipids in iron-starved *Chlamydomonas*. J Biol Chem.

[62] Kim S-H, Liu K-H, Lee S-Y, Hong S-J, Cho B-K, Lee H, Lee C-G, Choi H-K (2013). Effects of light intensity and nitrogen starvation on glycerolipid, glycerophospholipid, and carotenoid composition in *Dunaliella tertiolecta* culture. PLoS One.

[63] Yao L, Shen H, Wang N, Tatlay J, Li L, Tan TW, Lee YK: Elevated acetyl-CoA by amino acid recycling fuels microalgal neutral lipid accumulation in exponential growth phase for biofuel production. Plant Biotechnol J. 2016. doi: 10.1111/pbi.12648. 10.1111/pbi.12648 PMC5362678 27734577

