# Supplementary material for: Exploring the transcriptome of non-model oleaginous microalga Dunaliella tertiolecta through high-throughput sequencing and high performance computing
Source: BMC Bioinformatics. 2017 Feb 22;18:122. doi: 10.1186/s12859-017-1551-x (PMC5322580; doi:10.1186/s12859-017-1551-x)
Supplement: Additional file 1: — Detailed steps of transcriptome construction and analyses. (a) Trinity (v2.2.0) for assembly of transcriptome; (b) mpiBLASTX (v1.6.0) for comparison of homologous sequences; (c) Dt_v11.fa and Dt_v11.2.fa transcript files and their annotation information were generated via optimized Bag2D to filter the redundant contigs and get the top-hits within the criteria; (d) RSEM (v1.2.29) for mapping the sequencing reads. (DOCX 104 kb) [file 12859_2017_1551_MOESM1_ESM.docx]

**Additional file 1 - Detailed steps of transcriptome construction and analyses.**

**(a) Trinity (v2.2.0) for assembly of transcriptome.**

Usage:

$TRINITY_HOME/Trinity --seqType fq \

--CPU 96 --max_memory 2048G \

--SS_lib_type RF --no_bowtie --no_cleanup \

--left $INPUT/left_data1.fastq.gz $INPUT/left_data2.fastq.gz $INPUT/left_data3.fastq.gz

--right $INPUT/right_data1.fastq.gz $INPUT/right_data2.fastq.gz

$INPUT/right_data3.fastq.gz

--output $OUTPUT/Trinity.fasta

Note: we used 96 threads as each node of the data center is equipped with 48 CPU cores, and each core has 2 hardware threads. 2048 GB memory RAM was allocated to accommodate to the assembler’s intensive memory use. seqType fq for fastq format data, SS_lib_type RF for paired-end of sequencing library, left_data.fastq.gz and right_data.fastq.gz for a pair of left and right sequencing files in fastq format. The output file was generated in Trinity.fasta under OUTPUT directory.

**(b) mpiBLASTX (v1.6.0) for comparison of homologous sequences.**

Usage:

### Set the number of cores (cpus) and memory that will be used for this job

#PBS -l select=40:ncpus=24:mem=60gb

mpirun -np 960 mpiblast -p blastx -d combined.faa -i $INPUT/Dt_merged.fa

--use-virtual-frags --removedb \

-o $OUTPUT/blast_output.txt

Note: where -np 960 stands for using 960 (40 nodes) cores, -p blastx stands for using blastx software under blast suites, -d combined.faa stands for a combined protein reference file from all plants and bacterial after mpiformatdb, -i $INPUT/Dt_merged.fa stands for the inquiry file of the merged *D. tertiolecta* contigs from outputs of Trinity, --use-virtual-frags enable workers to cache database fragments in memory instead of local storage, --removedb removes the local copy of the database from each node before terminating execution. The blastx output was generated into blast_output.txt under OUTPUT directory.

**(c) Dt_v11.fa and Dt_v11.2.fa transcript files and their annotation information were generated via optimized Bag2D to filter the redundant contigs and get the top-hits within the criteria.**

**(d) RSEM (v1.2.29) for mapping the sequencing reads.**

Usage:

### Step 1: prepare reference

$RSEM_HOME/rsem-prepare-reference --bowtie2 $INPUT/Dt_v11.2.fasta $INPUT/prepare_reference

### Step 2: calculate expression

$RSEM_HOME/rsem-calculate-expression --paired-end \

$INPUT/left_data_x1.fastq.gz $INPUT/right_data_x1.fastq.gz \

--bowtie2 $INPUT/prepare_reference $OUTPUT/expression_genes_x1.results -p 48

$INPUT/left_data_x2.fastq.gz $INPUT/right_data_x2.fastq.gz \

--bowtie2 $INPUT/prepare_reference $OUTPUT/expression_genes_x2.results -p 48

$INPUT/left_data_x3.fastq.gz $INPUT/right_data_x3.fastq.gz \

--bowtie2 $INPUT/prepare_reference $OUTPUT/expression_genes_x3.results -p 48

$INPUT/left_data_wt1.fastq.gz $INPUT/right_data_wt1.fastq.gz \

--bowtie2 $INPUT/prepare_reference $OUTPUT/expression_genes_wt1.results -p 48

$INPUT/left_data_wt2.fastq.gz $INPUT/right_data_wt2.fastq.gz \

--bowtie2 $INPUT/prepare_reference $OUTPUT/expression_genes_wt2.results -p 48

$INPUT/left_data_wt3.fastq.gz $INPUT/right_data_wt3.fastq.gz \

--bowtie2 $INPUT/prepare_reference $OUTPUT/expression_genes_wt3.results -p 48

### Step 3: generate_data_matrix

$RSEM_HOME/rsem-generate-data-matrix \

$OUTPUT/expression_genes_x1.results.genes.results \

$OUTPUT/expression_genes_x2.results.genes.results \

$OUTPUT/expression_genes_x3.results.genes.results \

$OUTPUT/expression_genes_wt1.results.genes.results \

$OUTPUT/expression_genes_wt2.results.genes.results \

$OUTPUT/expression_genes_wt3.results.genes.results \

>$OUTPUT/GeneMat_x.txt

Note: where reads are aligned to the non-redundant transcript sequences using bowtie2 and read count per transcript is estimated by RSEM, using paired-end of sequencing data of treated samples - x1.fastq, x2.fastq, x3.fastq (biological triplicates), and control samples - wt1.fastq, wt2.fastq, wt3.fastq (biological triplicates). -p 48 stands for using 48 threads. A data matrix file of GeneMat_x.txt was generated under OUTPUT directory.

**(e) EbSeq (in RSEM package) for gene differential expression analysis.**

### Step 4: run_EbSeq

$RSEM_HOME/rsem-run-ebseq $OUTPUT/GeneMat_x.txt 3,3 $OUTPUT/GeneMat_x.results

### Step 5: FDR0.05

$RSEM_HOME/rsem-control-fdr $OUTPUT/GeneMat_x.results 0.05 $OUTPUT/GeneMat_x.de.txt

Note: based on the read count, differential expression changes with false discovery rate (FDR) < 0.05 was generated in GeneMat_x.de.txt under OUTPUT directory.
