# Supplementary material for: Exploring the transcriptome of non-model oleaginous microalga Dunaliella tertiolecta through high-throughput sequencing and high performance computing
Source: BMC Bioinformatics. 2017 Feb 22;18:122. doi: 10.1186/s12859-017-1551-x (PMC5322580; doi:10.1186/s12859-017-1551-x)

**Additional file 2 - Venn diagram of the numbers of *D. tertiolecta* transcripts with BLASTX hits of alternative-splicing variants from four organisms.**

A, *A. thaliana*; B, *C. reinhardtii*; C, *V. carteri*; E, *D. salina*.


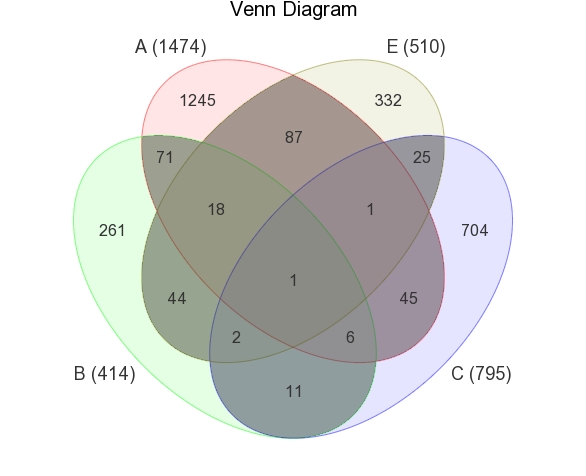

Supplement: Additional file 2: — Venn diagram of the numbers of D. tertiolecta transcripts with BLASTX hits of alternative-splicing variants from four organisms. A, A. thaliana; B, C. reinhardtii; C, V. carteri; E, D. salina. (DOCX 132 kb) [file 12859_2017_1551_MOESM2_ESM.docx]
