# Supplementary material for: Exploring the transcriptome of non-model oleaginous microalga Dunaliella tertiolecta through high-throughput sequencing and high performance computing
Source: BMC Bioinformatics. 2017 Feb 22;18:122. doi: 10.1186/s12859-017-1551-x (PMC5322580; doi:10.1186/s12859-017-1551-x)
Supplement: Additional file 3: — List of differential expression genes in nitrogen-deprived D. tertiolecta cells in Dt_v10 and Dt_v11 analyses. (a) Dt_v10 analysis; (b) Dt_v11 analysis. (DOCX 401 kb) [file 12859_2017_1551_MOESM3_ESM.docx]

**Additional file 3 - List of differential expression genes in nitrogen-deprived *D. tertiolecta* cells in Dt_v10 and Dt_v11 analyses.**

(a) Dt_v10 analysis

| **Dt_name** | **Cre_proteinID** | **Cre_proteinName** | **Cre_transcriptID** | **Cre_gene_name** | **Cre_geneID** | **FDR** | **Fold-Change** |
| --- | --- | --- | --- | --- | --- | --- | --- |
| Locus_6668_3Transcript_1/2_Confidence_0.750_Length_1172 | Cre43.g787650.t1.1 | Transducin/WD40 repeat-like superfamily protein | CHLREDRAFT_348286 | ? | estExt_fgenesh1_pg.C_430004 | 4.05E-02 | -3.12E+00 |
| Locus_11_3Transcript_5254/11887_Confidence_1.000_Length_1243 | Cre32.g781750.t1.1 | chloroplast signal recognition particle component (CAO) | CHLREDRAFT_22212 | SRP43 | fgenesh1_pg.C_scaffold_94000029 | 4.17E-06 | -1.22E+01 |
| Locus_1375_7Transcript_4/9_Confidence_0.579_Length_1179 | Cre32.g781300.t1.1 | photosystem II light harvesting complex gene 2.1 | CHLREDRAFT_195162 | LHCBM3 | estExt_fgenesh2_pg.C_940011 | 7.21E-04 | -4.32E+01 |
| Locus_945_3Transcript_3/3_Confidence_0.846_Length_2146 | Cre29.g778200.t1.1 | Dihydrolipoamide acetyltransferase, long form protein | CHLREDRAFT_149709 | DLA1 | Chlre2_kg.scaffold_25000188 | 2.56E-05 | 2.65E+00 |
| Locus_1809_2Transcript_3/3_Confidence_0.800_Length_4259 | Cre28.g776100.t1.1 | carbamoyl phosphate synthetase B | CHLREDRAFT_195255 | CMPL1 | estExt_fgenesh2_pg.C_1020002 | 3.80E-10 | 2.53E+00 |
| Locus_6242_9Transcript_1/1_Confidence_1.000_Length_872 | Cre27.g774650.t1.2 | Ribosome recycling factor | CHLREDRAFT_194988 | CHLREDRAFT_194988 | estExt_fgenesh2_pg.C_840011 | 2.11E-02 | -3.26E+00 |
| Locus_626_4Transcript_1/1_Confidence_1.000_Length_675 | Cre26.g773000.t1.2 | nucleoside diphosphate kinase 2 | CHLREDRAFT_292075 | ? | au.g9990_t1 | 1.42E-14 | -3.21E+00 |
| Locus_11049_9Transcript_1/1_Confidence_1.000_Length_444 | Cre24.g770450.t1.1 | basic helix-loop-helix (bHLH) DNA-binding superfamily protein | CHLREDRAFT_408144 | ? | pasa_Sanger_mRNA34961 | 3.60E-05 | -3.77E+00 |
| Locus_7053_2Transcript_1/1_Confidence_1.000_Length_983 | Cre23.g767050.t1.2 | ACT domain-containing protein | CHLREDRAFT_391513 | ? | pasa_Sanger_mRNA34882 | 2.27E-07 | 4.63E+00 |
| Locus_5306_7Transcript_3/5_Confidence_0.704_Length_718 | Cre23.g766250.t1.1 | photosystem II light harvesting complex gene 2.2 | CHLREDRAFT_185533 | LHCBM1 | estExt_fgenesh2_kg.C_510040 | 2.82E-04 | -2.13E+01 |
| Locus_3330_2Transcript_1/1_Confidence_1.000_Length_2528 | Cre23.g765700.t1.1 | acetyl-CoA synthetase | CHLREDRAFT_194063 | ACS1 | estExt_fgenesh2_pg.C_510100 | 3.05E-03 | -4.34E+00 |
| Locus_3770_8Transcript_1/1_Confidence_1.000_Length_2236 | Cre22.g765250.t1.1 | 3-ketoacyl-acyl carrier protein synthase I | CHLREDRAFT_205887 | KAS1 | SKA_estExt_fgenesh2_kg.C_620002 | 9.62E-10 | -3.61E+00 |
| Locus_12490_1Transcript_1/1_Confidence_1.000_Length_1014 | Cre22.g765200.t1.1 | NA | CHLREDRAFT_397915 | ? | pasa_Sanger_mRNA34705 | 2.97E-02 | -4.68E+00 |
| Locus_1093_4Transcript_1/1_Confidence_1.000_Length_1820 | Cre22.g763250.t1.1 | Phosphoglycerate kinase family protein | CHLREDRAFT_132210 | PGK1 | estExt_gwp_1H.C_480026 | 1.52E-06 | -5.56E+00 |
| Locus_43614_10Transcript_4/4_Confidence_0.769_Length_1176 | Cre22.g763000.t1.2 | uracil phosphoribosyltransferase | CHLREDRAFT_381966 | ? | estExt_fgenesh1_pm.C_220003 | 8.08E-08 | -5.78E+00 |
| Locus_9607_9Transcript_1/1_Confidence_1.000_Length_1658 | Cre22.g762850.t1.1 | ABC transporter family protein | CHLREDRAFT_132213 | FAP151 | estExt_gwp_1H.C_480029 | 1.27E-02 | 2.02E+00 |
| Locus_4714_2Transcript_1/1_Confidence_1.000_Length_2709 | Cre19.g755050.t1.1 | isoamylase 3 | CHLREDRAFT_132067 | ISA3 | estExt_gwp_1H.C_470047 | 3.11E-02 | 2.51E+00 |
| Locus_15047_7Transcript_1/2_Confidence_0.400_Length_1725 | Cre19.g751700.t1.1 | Chlorophyll A-B binding family protein | CHLREDRAFT_185309 | LHL3 | estExt_fgenesh2_kg.C_470002 | 5.13E-29 | -3.61E+01 |
| Locus_3256_5Transcript_1/1_Confidence_1.000_Length_1857 | Cre19.g751650.t1.1 | pyrimidine d | CHLREDRAFT_152239 | PYR4 | Chlre2_kg.scaffold_47000002 | 3.08E-05 | 4.62E+00 |
| Locus_241_4Transcript_1/1_Confidence_1.000_Length_1001 | Cre18.g749900.t1.1 | ATP synthase delta-subunit gene | CHLREDRAFT_132678 | ATPD | estExt_gwp_1H.C_660032 | 3.04E-11 | -2.74E+00 |
| Locus_522_6Transcript_1/7_Confidence_0.209_Length_1394 | Cre18.g749750.t1.1 | photosystem I light harvesting complex gene 3 | CHLREDRAFT_153678 | LHCA3 | Chlre2_kg.scaffold_66000007 | 1.79E-02 | -5.29E+00 |
| Locus_889_6Transcript_1/2_Confidence_0.667_Length_3064 | Cre18.g748050.t1.2 | Guanylate-binding family protein | CHLREDRAFT_348025 | ? | estExt_fgenesh1_pg.C_180059 | 4.18E-02 | -4.21E+00 |
| Locus_3575_8Transcript_1/2_Confidence_0.857_Length_974 | Cre17.g729150.t1.1 | RNA-binding (RRM/RBD/RNP motifs) family protein with retrovirus zinc finger-like domain | CHLREDRAFT_119900 | CHLREDRAFT_119900 | e_gwW.32.150.1 | 2.27E-03 | -3.36E+00 |
| Locus_1408_4Transcript_1/1_Confidence_1.000_Length_1500 | Cre17.g727700.t1.2 | DEAD box RNA helicase (RH3) | CHLREDRAFT_119793 | CHLREDRAFT_119793 | e_gwW.32.49.1 | 3.65E-07 | 3.86E+00 |
| Locus_1363_2Transcript_1/2_Confidence_0.889_Length_4144 | Cre17.g723250.t1.1 | Insulinase (Peptidase family M16) family protein | CHLREDRAFT_206035 | CPE1 | OVA_e_gwW.32.1.1 | 1.02E-02 | 2.75E+00 |
| Locus_125_6Transcript_1/1_Confidence_1.000_Length_2052 | Cre17.g723150.t1.2 | endoplasmic reticulum oxidoreductins 2 | CHLREDRAFT_195663 | ERO1 | SAN_Chlre2_kg.scaffold_32000063 | 1.42E-02 | -6.09E+00 |
| Locus_730_1Transcript_2/2_Confidence_0.667_Length_1282 | Cre17.g722750.t1.1 | NA | CHLREDRAFT_381721 | ? | estExt_fgenesh1_pm.C_chromosome_170291 | 3.96E-02 | -2.39E+00 |
| Locus_13536_10Transcript_1/1_Confidence_1.000_Length_1269 | Cre17.g720250.t1.1 | light harvesting complex photosystem II | CHLREDRAFT_184810 | LHCB4 | estExt_fgenesh2_kg.C_320006 | 5.19E-04 | -2.29E+01 |
| Locus_7091_8Transcript_1/9_Confidence_0.104_Length_3735 | Cre17.g718300.t1.2 | CHY-type/CTCHY-type/RING-type Zinc finger protein | CHLREDRAFT_336653 | ? | pm.chromosome_5_#_133 | 2.35E-02 | -2.55E+00 |
| Locus_18018_1Transcript_1/1_Confidence_1.000_Length_805 | Cre17.g718250.t1.2 | NAD(P)-binding Rossmann-fold superfamily protein | CHLREDRAFT_123563 | CHLREDRAFT_123563 | e_gwW.97.14.1 | 2.60E-03 | -1.09E+01 |
| Locus_6040_6Transcript_1/1_Confidence_1.000_Length_981 | Cre17.g715750.t1.1 | Transducin/WD40 repeat-like superfamily protein | CHLREDRAFT_139713 | SEC13 | estExt_gwp_1W.C_540024 | 4.91E-02 | -2.63E+00 |
| Locus_12546_7Transcript_1/1_Confidence_1.000_Length_726 | Cre17.g715250.t1.1 | biotin carboxyl carrier protein 2 | CHLREDRAFT_185478 | BCC1 | estExt_fgenesh2_kg.C_540006 | 9.44E-03 | -7.12E+00 |
| Locus_327_6Transcript_3/10_Confidence_0.333_Length_548 | Cre17.g710450.t1.1 | Histone superfamily protein | CHLREDRAFT_112705 | HTB13 | e_gwW.3.291.1 | 2.63E-03 | -2.24E+00 |
| Locus_143_2Transcript_1/1_Confidence_1.000_Length_889 | Cre17.g708000.t1.2 | NA | CHLREDRAFT_140976 | CHLREDRAFT_140976 | Chlre2_kg.scaffold_8000147 | 3.04E-02 | 8.80E+00 |
| Locus_1123_5Transcript_2/4_Confidence_0.727_Length_3018 | Cre17.g703800.t1.2 | solute:sodium symporters;urea transmembrane transporters | CHLREDRAFT_170417 | DUR3C | fgenesh2_pg.C_scaffold_13000168 | 2.80E-60 | 7.26E+01 |
| Locus_1487_6Transcript_4/4_Confidence_0.769_Length_2691 | Cre17.g703800.t1.2 | solute:sodium symporters;urea transmembrane transporters | CHLREDRAFT_196479 | DUR3A | SKA_e_gwH.77.26.1 | 2.19E-32 | 6.50E+01 |
| Locus_12493_9Transcript_1/1_Confidence_1.000_Length_1719 | Cre17.g703700.t1.1 | ATP citrate lyase (ACL) family protein | CHLREDRAFT_24101 | SCLB1a | estExt_fgenesh1_pm.C_130018 | 2.01E-06 | 3.58E+00 |
| Locus_4659_7Transcript_1/1_Confidence_1.000_Length_1270 | Cre17.g703200.t1.1 | SEC14-like 12 | CHLREDRAFT_347673 | ? | estExt_fgenesh1_pg.C_chromosome_170094 | 2.47E-03 | -4.66E+00 |
| Locus_2443_1Transcript_2/2_Confidence_0.929_Length_1166 | Cre17.g702200.t1.1 | ankyrin repeat-containing protein 2 | CHLREDRAFT_189100 | ANK29 | estExt_fgenesh2_pg.C_130191 | 1.35E-04 | -5.15E+00 |
| Locus_6115_3Transcript_1/1_Confidence_1.000_Length_1274 | Cre17.g699900.t1.1 | NAD(P)-linked oxidoreductase superfamily protein | CHLREDRAFT_115347 | CHLREDRAFT_115347 | e_gwW.13.210.1 | 1.98E-02 | -2.11E+00 |
| Locus_32128_9Transcript_1/1_Confidence_1.000_Length_605 | Cre17.g699600.t1.1 | sedoheptulose-bisphosphatase | CHLREDRAFT_206057 | SEBP2 | MHS_OVA_BTM_Chlre2_kg.scaffold_13000210 | 6.06E-03 | 3.65E+00 |
| Locus_995_2Transcript_1/1_Confidence_1.000_Length_1232 | Cre16.g693819.t1.1 | Protein of unknown function (DUF506) | CHLREDRAFT_396739 | ? | pasa_Sanger_mRNA14908 | 4.68E-04 | -8.51E+00 |
| Locus_4133_3Transcript_3/6_Confidence_0.100_Length_1053 | Cre16.g692850.t1.2 | Cobalamin biosynthesis CobW-like protein | CHLREDRAFT_288765 | ? | au.g6909_t1 | 1.03E-06 | -4.06E+00 |
| Locus_1062_8Transcript_1/1_Confidence_1.000_Length_4619 | Cre16.g691800.t1.2 | NA | CHLREDRAFT_193711 | CHLREDRAFT_193711 | estExt_fgenesh2_pg.C_460027 | 1.45E-04 | 3.21E+00 |
| Locus_411_1Transcript_5/7_Confidence_0.136_Length_2557 | Cre16.g689150.t1.1 | sulfoquinovosyldiacylglycerol 2 | CHLREDRAFT_116277 | SQD2 | e_gwW.1.590.1 | 9.22E-03 | -5.88E+00 |
| Locus_829_6Transcript_2/9_Confidence_0.609_Length_1544 | Cre16.g689150.t1.1 | sulfoquinovosyldiacylglycerol 2 | CHLREDRAFT_121326 | SQD3 | e_gwW.45.101.1 | 1.42E-14 | -6.41E+00 |
| Locus_1863_9Transcript_1/1_Confidence_1.000_Length_1202 | Cre16.g687900.t1.1 | photosystem I light harvesting complex gene 5 | CHLREDRAFT_183363 | LHCA7 | estExt_fgenesh2_kg.C_140100 | 1.25E-03 | -7.41E+00 |
| Locus_15070_10Transcript_1/1_Confidence_1.000_Length_368 | Cre16.g687300.t1.1 | Tetratricopeptide repeat (TPR)-like superfamily protein | CHLREDRAFT_115715 | CHLREDRAFT_115715 | e_gwW.14.185.1 | 5.85E-04 | -3.20E+00 |
| Locus_13605_5Transcript_1/1_Confidence_1.000_Length_745 | Cre16.g681500.t1.1 | NA | CHLREDRAFT_397482 | ? | pasa_Sanger_mRNA14612.1 | 1.50E-02 | -9.02E+00 |
| Locus_5432_8Transcript_1/1_Confidence_1.000_Length_990 | Cre16.g680700.t1.1 | Aluminium induced protein with YGL and LRDR motifs | CHLREDRAFT_184434 | CHLREDRAFT_184434 | estExt_fgenesh2_kg.C_280076 | 3.09E-04 | 1.23E+01 |
| Locus_2863_1Transcript_2/2_Confidence_0.857_Length_1204 | Cre16.g680000.t1.1 | delta subunit of Mt ATP synthase | CHLREDRAFT_191596 | ATP5 | estExt_fgenesh2_pg.C_280199 | 6.22E-09 | 2.53E+00 |
| Locus_2975_2Transcript_1/1_Confidence_1.000_Length_727 | Cre16.g679300.t1.1 | NA | CHLREDRAFT_288473 | ? | au.g6639_t1 | 2.16E-03 | -1.22E+01 |
| Locus_4753_10Transcript_1/1_Confidence_1.000_Length_1116 | Cre16.g678000.t1.1 | Protein of unknown function (DUF3411) | CHLREDRAFT_381391 | ? | estExt_fgenesh1_pm.C_chromosome_160263 | 4.32E-04 | 2.24E+00 |
| Locus_9076_9Transcript_1/2_Confidence_0.750_Length_1074 | Cre16.g677450.t1.1 | Galactose mutarotase-like superfamily protein | CHLREDRAFT_397276 | ? | pasa_Sanger_mRNA15653 | 1.42E-04 | -2.52E+00 |
| Locus_2884_9Transcript_2/3_Confidence_0.636_Length_1541 | Cre16.g676250.t1.1 | NA | CHLREDRAFT_149177 | CHLREDRAFT_149177 | Chlre2_kg.scaffold_28000125 | 1.36E-03 | 3.07E+00 |
| Locus_10864_8Transcript_1/1_Confidence_1.000_Length_834 | Cre16.g675550.t1.1 | FK506-binding protein 13 | CHLREDRAFT_195500 | FKB16-2a | OVA_Chlre2_kg.scaffold_28000114 | 1.35E-06 | -3.89E+00 |
| Locus_5048_10Transcript_1/1_Confidence_1.000_Length_887 | Cre16.g674300.t1.1 | ankyrin repeat-containing protein 2 | CHLREDRAFT_149145 | FAP269 | Chlre2_kg.scaffold_28000093 | 1.82E-11 | -7.68E+00 |
| Locus_1504_7Transcript_1/3_Confidence_0.727_Length_1210 | Cre16.g673650.t1.1 | light harvesting complex of photosystem II 5 | CHLREDRAFT_184397 | LHCB5 | estExt_fgenesh2_kg.C_280035 | 1.06E-03 | -1.12E+01 |
| Locus_4770_7Transcript_1/1_Confidence_1.000_Length_466 | Cre16.g672950.t1.1 | NA | CHLREDRAFT_80348 | LCI2 | estExt_GenewiseW_1.C_280074 | 1.03E-04 | -5.66E+00 |
| Locus_13703_7Transcript_2/2_Confidence_0.800_Length_1663 | Cre16.g665250.t1.1 | acclimation of photosynthesis to environment | CHLREDRAFT_194448 | APE1 | estExt_fgenesh2_pg.C_670042 | 2.17E-06 | -6.36E+00 |
| Locus_491_5Transcript_1/1_Confidence_1.000_Length_2220 | Cre16.g664550.t1.1 | serine transhydroxymethyltransferase 1 | CHLREDRAFT_194461 | SHMT1 | estExt_fgenesh2_pg.C_670056 | 3.79E-03 | -2.08E+00 |
| Locus_565_3Transcript_1/1_Confidence_1.000_Length_1664 | Cre16.g663900.t1.1 | hydroxymethylbilane synthase | CHLREDRAFT_194475 | PBGD1 | estExt_fgenesh2_pg.C_670072 | 1.75E-18 | -1.50E+01 |
| Locus_8403_8Transcript_1/2_Confidence_0.800_Length_2020 | Cre16.g662000.t1.1 | P-loop containing nucleoside triphosphate hydrolases superfamily protein | CHLREDRAFT_288104 | ? | au.g6300_t1 | 1.32E-02 | 3.12E+00 |
| Locus_1049_4Transcript_1/1_Confidence_1.000_Length_1494 | Cre16.g656400.t1.1 | sulfoquinovosyldiacylglycerol 1 | CHLREDRAFT_27658 | SQD1 | estExt_fgenesh1_kg.C_370003 | 5.05E-26 | -8.21E+00 |
| Locus_2409_7Transcript_1/1_Confidence_1.000_Length_1014 | Cre16.g655150.t1.1 | Tetratricopeptide repeat (TPR)-like superfamily protein | CHLREDRAFT_193119 | CHLREDRAFT_193119 | estExt_fgenesh2_pg.C_390004 | 4.13E-03 | -2.80E+00 |
| Locus_3089_4Transcript_1/1_Confidence_1.000_Length_983 | Cre16.g655050.t1.1 | NA | CHLREDRAFT_318546 | ? | kg.chromosome_16_#_218_#_KCC003178A_C01 | 8.09E-05 | 2.99E+00 |
| Locus_4110_9Transcript_1/1_Confidence_1.000_Length_766 | Cre16.g652550.t1.1 | Translation protein SH3-like family protein | CHLREDRAFT_195622 | PRPL24 | MAY_acegs_kg.scaffold_39000017 | 1.59E-05 | -2.40E+00 |
| Locus_36616_9Transcript_1/1_Confidence_1.000_Length_300 | Cre16.g650100.t1.1 | electron transporter, transferring electrons within cytochrome b6/f complex of photosystem IIs | CHLREDRAFT_177155 | PETN | fgenesh2_pg.C_scaffold_39000105 | 3.01E-04 | -2.81E+00 |
| Locus_1442_6Transcript_1/1_Confidence_1.000_Length_1413 | Cre15.g646250.t1.1 | NAD(P)-binding Rossmann-fold superfamily protein | CHLREDRAFT_403637 | ? | pasa_Sanger_mRNA13783 | 4.10E-05 | -4.88E+00 |
| Locus_7194_2Transcript_1/1_Confidence_1.000_Length_2586 | Cre15.g645950.t1.1 | cytochrome P450, family 97, subfamily A, polypeptide 3 | CHLREDRAFT_196742 | CYP97A5 | DNE_DNE_gwH.55.10.1 | 8.49E-03 | -3.72E+00 |
| Locus_37063_10Transcript_1/1_Confidence_1.000_Length_796 | Cre14.g626900.t1.1 | phosphomannomutase | CHLREDRAFT_394918 | ? | pasa_Sanger_mRNA12389 | 1.45E-04 | -3.22E+00 |
| Locus_12069_5Transcript_2/2_Confidence_0.667_Length_970 | Cre14.g621650.t1.2 | catalytics;transferases;[acyl-carrier-protein] S-malonyltransferases;binding | CHLREDRAFT_141466 | CHLREDRAFT_141466 | Chlre2_kg.scaffold_7000293 | 1.58E-05 | -4.72E+00 |
| Locus_1463_4Transcript_1/1_Confidence_1.000_Length_815 | Cre14.g621550.t1.1 | thioredoxin M-type 4 | CHLREDRAFT_136413 | TRXm | estExt_gwp_1W.C_10120 | 2.61E-23 | -3.33E+00 |
| Locus_9096_1Transcript_1/3_Confidence_0.429_Length_2404 | Cre14.g620350.t1.1 | GTP cyclohydrolase II | CHLREDRAFT_102099 | CHLREDRAFT_102099 | e_gwH.1.174.1 | 3.57E-02 | -3.98E+00 |
| Locus_3924_6Transcript_2/2_Confidence_0.909_Length_2549 | Cre14.g619150.t1.2 | succinate dehydrogenase 1-1 | CHLREDRAFT_394775 | ? | pasa_Sanger_mRNA12217 | 1.01E-07 | 4.42E+00 |
| Locus_2053_5Transcript_2/4_Confidence_0.625_Length_1570 | Cre14.g619050.t1.1 | NA | CHLREDRAFT_147028 | CHLREDRAFT_147028 | Chlre2_kg.scaffold_1000852 | 2.07E-04 | -2.26E+00 |
| Locus_2827_3Transcript_5/10_Confidence_0.222_Length_1532 | Cre14.g616600.t1.1 | FZO-like | CHLREDRAFT_190184 | CHLREDRAFT_190184 | estExt_fgenesh2_pg.C_10834 | 4.55E-02 | -3.30E+00 |
| Locus_2006_2Transcript_1/1_Confidence_1.000_Length_4325 | Cre14.g615950.t1.1 | general control non-repressible 4 | CHLREDRAFT_145770 | CHLREDRAFT_145770 | Chlre2_kg.scaffold_15000084 | 9.35E-05 | 3.16E+00 |
| Locus_316_3Transcript_1/6_Confidence_0.250_Length_794 | Cre14.g615400.t1.1 | NA | CHLREDRAFT_81856 | THB1 | estExt_GenewiseW_1.C_430050 | 4.23E-02 | -2.98E+02 |
| Locus_1570_3Transcript_1/3_Confidence_0.667_Length_641 | Cre14.g615350.t1.1 | NA | CHLREDRAFT_196750 | THB2 | OVA_estExt_GenewiseH_1.C_430038 | 2.60E-02 | 2.11E+00 |
| Locus_11593_9Transcript_1/1_Confidence_1.000_Length_580 | Cre14.g615000.t1.2 | methionine sulfoxide reductase B 2 | CHLREDRAFT_380981 | ? | estExt_fgenesh1_pm.C_chromosome_140046 | 1.87E-06 | -3.45E+00 |
| Locus_145_3Transcript_1/4_Confidence_0.650_Length_2792 | Cre14.g611950.t1.2 | Pentatricopeptide repeat (PPR) superfamily protein | CHLREDRAFT_395355 | ? | pasa_Sanger_mRNA12661 | 8.06E-03 | -3.93E+00 |
| Locus_14105_7Transcript_1/1_Confidence_1.000_Length_920 | Cre14.g609400.t1.2 | NA | CHLREDRAFT_194484 | CHLREDRAFT_194484 | estExt_fgenesh2_pg.C_650014 | 7.72E-04 | -8.02E+00 |
| Locus_3007_2Transcript_1/1_Confidence_1.000_Length_1653 | Cre13.g608000.t1.1 | NAD(P)-binding Rossmann-fold superfamily protein | CHLREDRAFT_193550 | CPLD52 | estExt_fgenesh2_pg.C_440099 | 1.43E-05 | -4.77E+00 |
| Locus_1378_3Transcript_2/3_Confidence_0.643_Length_2363 | Cre13.g606150.t1.1 | NA | CHLREDRAFT_185295 | CHLREDRAFT_185295 | estExt_fgenesh2_kg.C_440015 | 4.79E-02 | 3.60E+00 |
| Locus_2001_4Transcript_1/1_Confidence_1.000_Length_1342 | Cre13.g603700.t1.1 | actin-11 | CHLREDRAFT_24114 | ARP2 | estExt_fgenesh1_pm.C_140011 | 2.80E-03 | -2.20E+00 |
| Locus_2122_6Transcript_1/1_Confidence_1.000_Length_997 | Cre13.g602650.t1.1 | Rhodanese/Cell cycle control phosphatase superfamily protein | CHLREDRAFT_286834 | ? | au.g5112_t1 | 1.63E-02 | -1.79E+01 |
| Locus_5338_5Transcript_1/1_Confidence_1.000_Length_2543 | Cre13.g598750.t1.2 | phosphoglucomutase | CHLREDRAFT_18029 | ? | fgenesh1_pg.C_scaffold_38000068 | 2.78E-06 | -2.40E+00 |
| Locus_2591_3Transcript_1/1_Confidence_1.000_Length_768 | Cre13.g598050.t1.2 | NA | CHLREDRAFT_192969 | CHLREDRAFT_192969 | estExt_fgenesh2_pg.C_380078 | 1.64E-03 | -2.02E+00 |
| Locus_1927_4Transcript_1/1_Confidence_1.000_Length_1397 | Cre13.g592950.t1.1 | NA | CHLREDRAFT_193011 | CHLREDRAFT_193011 | estExt_fgenesh2_pg.C_380156 | 2.58E-07 | -2.74E+00 |
| Locus_218_4Transcript_1/1_Confidence_1.000_Length_3221 | Cre13.g592200.t1.1 | NADH-dependent glutamate synthase 1 | CHLREDRAFT_205746 | GSN1 | MHS_estExt_gwp_1W.C_180086 | 1.90E-13 | 2.65E+01 |
| Locus_17932_5Transcript_1/1_Confidence_1.000_Length_906 | Cre13.g589350.t1.1 | NA | CHLREDRAFT_188114 | FBB6 | estExt_fgenesh2_pg.C_20484 | 3.91E-02 | -2.58E+00 |
| Locus_9589_8Transcript_1/1_Confidence_1.000_Length_648 | Cre13.g588100.t1.1 | rotamase cyclophilin 2 | CHLREDRAFT_30639 | CYN19-3 | estExt_fgenesh1_pg.C_20512 | 7.42E-08 | -5.96E+00 |
| Locus_2754_6Transcript_1/2_Confidence_0.800_Length_1330 | Cre13.g583550.t1.1 | plastid transcriptionally active 4 | CHLREDRAFT_134824 | VIPP1 | estExt_gwp_1W.C_20003 | 1.76E-05 | -3.35E+00 |
| Locus_2841_6Transcript_1/1_Confidence_1.000_Length_957 | Cre13.g581650.t1.1 | ribosomal protein L12-A | CHLREDRAFT_99271 | PRPL7/L12 | e_gwH.2.436.1 | 1.73E-05 | -3.43E+00 |
| Locus_100_5Transcript_43/88_Confidence_1.000_Length_840 | Cre13.g577850.t1.2 | FKBP-like peptidyl-prolyl cis-trans isomerase family protein | CHLREDRAFT_188000 | FKB20-2 | estExt_fgenesh2_pg.C_20293 | 1.24E-02 | -1.05E+01 |
| Locus_1309_4Transcript_1/1_Confidence_1.000_Length_723 | Cre13.g577100.t1.1 | acyl carrier protein 1 | CHLREDRAFT_187994 | ACP2 | estExt_fgenesh2_pg.C_20278 | 8.69E-07 | -4.02E+00 |
| Locus_3230_2Transcript_2/2_Confidence_0.556_Length_1009 | Cre13.g573400.t1.1 | NA | CHLREDRAFT_182705 | CHLREDRAFT_182705 | estExt_fgenesh2_kg.C_20063 | 2.23E-27 | -8.68E+00 |
| Locus_3330_6Transcript_2/2_Confidence_0.933_Length_2732 | Cre13.g571700.t1.2 | calmodulin-domain protein kinase cdpk isoform 2 | CHLREDRAFT_316236 | ? | kg.chromosome_13_#_381_#_862300:1 | 1.60E-05 | 3.21E+01 |
| Locus_889_2Transcript_2/10_Confidence_0.500_Length_419 | Cre13.g570050.t1.1 | Histone superfamily protein | CHLREDRAFT_168516 | HTB2 | fgenesh2_pg.C_scaffold_2000162 | 1.74E-04 | -2.64E+00 |
| Locus_90_3Transcript_3/12_Confidence_0.324_Length_1222 | Cre13.g569850.t1.1 | ammonium transporter 1;2 | CHLREDRAFT_39642 | AMT1F | gwH.71.9.1 | 5.57E-08 | 2.20E+00 |
| Locus_7912_5Transcript_1/1_Confidence_1.000_Length_2481 | Cre13.g566000.t1.1 | 10-formyltetrahydrofolate synthetase | CHLREDRAFT_380616 | ? | estExt_fgenesh1_pm.C_chromosome_130042 | 1.10E-02 | -2.75E+00 |
| Locus_364_6Transcript_1/1_Confidence_1.000_Length_2119 | Cre13.g565450.t1.1 | L-Aspartase-like family protein | CHLREDRAFT_380611 | ? | estExt_fgenesh1_pm.C_chromosome_130034 | 3.47E-33 | 3.48E+00 |
| Locus_10542_10Transcript_1/2_Confidence_0.667_Length_292 | Cre13.g563050.t1.1 | NA | CHLREDRAFT_286003 | ? | au.g4323_t1 | 4.06E-05 | -5.69E+00 |
| Locus_21493_10Transcript_1/2_Confidence_0.667_Length_524 | Cre13.g562900.t1.1 | NA | CHLREDRAFT_187840 | CHLREDRAFT_187840 | estExt_fgenesh2_pg.C_20021 | 2.01E-04 | -4.32E+01 |
| Locus_115_4Transcript_1/1_Confidence_1.000_Length_1276 | Cre13.g562850.t1.1 | photosystem II reaction center PSB29 protein | CHLREDRAFT_182653 | THF1 | estExt_fgenesh2_kg.C_20004 | 3.52E-15 | -4.43E+00 |
| Locus_4270_9Transcript_3/3_Confidence_0.714_Length_1916 | Cre13.g562750.t1.1 | NA | CHLREDRAFT_412002 | ? | pasa_Sanger_mRNA10932 | 5.71E-06 | -3.70E+00 |
| Locus_466_5Transcript_3/4_Confidence_0.600_Length_1639 | Cre13.g561850.t1.1 | pfkB-like carbohydrate kinase family protein | CHLREDRAFT_380587 | ? | estExt_fgenesh1_pm.C_chromosome_130001 | 5.32E-09 | 6.67E+00 |
| Locus_17808_5Transcript_1/1_Confidence_1.000_Length_1364 | Cre12.g561000.t1.2 | Cyclophilin-like peptidyl-prolyl cis-trans isomerase family protein | CHLREDRAFT_37663 | CYN28 | estExt_fgenesh1_pg.C_870024 | 2.27E-03 | -4.40E+00 |
| Locus_37_4Transcript_1/1_Confidence_1.000_Length_664 | Cre12.g560950.t1.1 | photosystem I subunit G | CHLREDRAFT_165416 | PSAG | fgenesh2_kg.C_scaffold_87000009 | 4.83E-02 | -4.08E+00 |
| Locus_9645_3Transcript_1/3_Confidence_0.600_Length_728 | Cre12.g558000.t1.1 | Translation elongation factor EF1B/ribosomal protein S6 family protein | CHLREDRAFT_194972 | MRPS6 | estExt_fgenesh2_pg.C_800036 | 4.08E-02 | 2.30E+00 |
| Locus_3385_7Transcript_1/1_Confidence_1.000_Length_800 | Cre12.g557600.t1.1 | adenylate kinase 1 | CHLREDRAFT_133184 | ADK4 | estExt_gwp_1H.C_800036 | 3.85E-02 | -2.99E+00 |
| Locus_2045_7Transcript_1/1_Confidence_1.000_Length_811 | Cre12.g557050.t1.1 | YGGT family protein | CHLREDRAFT_154497 | CPLD8 | Chlre2_kg.scaffold_80000054 | 1.56E-05 | -6.50E+00 |
| Locus_2804_10Transcript_1/1_Confidence_1.000_Length_2543 | Cre12.g554250.t1.1 | UDP-glucose pyrophosphorylase 3 | CHLREDRAFT_196477 | ? | JLM_fgenesh2_pg.C_scaffold_20000272 | 2.21E-02 | -4.91E+00 |
| Locus_825_2Transcript_1/2_Confidence_0.889_Length_2524 | Cre12.g553250.t1.1 | phosphofructokinase 5 | CHLREDRAFT_196430 | PFK2 | MHS_estExt_gwp_1W.C_200088 | 1.29E-05 | 4.10E+00 |
| Locus_3217_6Transcript_3/3_Confidence_0.600_Length_1300 | Cre12.g552900.t1.1 | Transducin/WD40 repeat-like superfamily protein | CHLREDRAFT_380502 | ? | estExt_fgenesh1_pm.C_chromosome_120790 | 8.77E-03 | -9.58E+00 |
| Locus_2259_4Transcript_1/1_Confidence_1.000_Length_2802 | Cre12.g552200.t1.1 | alpha-glucan phosphorylase 2 | CHLREDRAFT_137300 | PHOB | estExt_gwp_1W.C_200107 | 9.74E-17 | 3.75E+00 |
| Locus_1104_4Transcript_1/1_Confidence_1.000_Length_411 | Cre12.g551950.t1.1 | Ankyrin repeat family protein | CHLREDRAFT_190875 | CGL38 | estExt_fgenesh2_pg.C_200221 | 1.58E-02 | -2.90E+00 |
| Locus_24226_1Transcript_1/1_Confidence_1.000_Length_463 | Cre12.g551900.t1.1 | Eukaryotic protein of unknown function (DUF842) | CHLREDRAFT_346503 | ? | estExt_fgenesh1_pg.C_chromosome_120912 | 4.46E-02 | 7.68E+00 |
| Locus_308_7Transcript_2/2_Confidence_0.750_Length_1082 | Cre12.g550850.t1.1 | photosystem II subunit P-1 | CHLREDRAFT_33411 | PSBP1 | estExt_fgenesh1_pg.C_200230 | 5.21E-04 | -1.70E+01 |
| Locus_2090_3Transcript_1/1_Confidence_1.000_Length_1829 | Cre12.g546050.t1.1 | 1-deoxy-D-xylulose 5-phosphate reductoisomerase | CHLREDRAFT_196606 | DXR1 | LOH_chlre3.20.176.3.11 | 1.48E-03 | -3.07E+00 |
| Locus_11516_7Transcript_1/1_Confidence_1.000_Length_963 | Cre12.g544150.t1.1 | cyclophilin 20-2 | CHLREDRAFT_184047 | CYN20-2 | estExt_fgenesh2_kg.C_200036 | 1.93E-03 | -3.38E+00 |
| Locus_1207_5Transcript_1/1_Confidence_1.000_Length_905 | Cre12.g544150.t1.1 | cyclophilin 20-2 | CHLREDRAFT_185571 | CYN20-3 | estExt_fgenesh2_kg.C_560003 | 7.23E-07 | -5.24E+00 |
| Locus_12223_8Transcript_1/1_Confidence_1.000_Length_1355 | Cre12.g538100.t1.1 | Glutathione S-transferase family protein | CHLREDRAFT_380384 | ? | estExt_fgenesh1_pm.C_chromosome_120630 | 3.37E-02 | -3.57E+00 |
| Locus_427_4Transcript_1/1_Confidence_1.000_Length_1348 | Cre12.g537200.t1.1 | 2-oxoglutarate dehydrogenase, E1 component | CHLREDRAFT_79471 | OGD1 | estExt_GenewiseW_1.C_180225 | 1.93E-07 | 2.64E+00 |
| Locus_67_2Transcript_1/1_Confidence_1.000_Length_3960 | Cre12.g534800.t1.1 | glycine decarboxylase P-protein 2 | CHLREDRAFT_136984 | GCSP | estExt_gwp_1W.C_180238 | 3.72E-03 | -2.47E+00 |
| Locus_6303_1Transcript_1/1_Confidence_1.000_Length_922 | Cre12.g534250.t1.1 | NA | CHLREDRAFT_157545 | CHLREDRAFT_157545 | acegs_kg.scaffold_18000043 | 3.86E-02 | -3.30E+00 |
| Locus_15351_2Transcript_1/1_Confidence_1.000_Length_761 | Cre12.g533800.t1.1 | Cyclophilin-like peptidyl-prolyl cis-trans isomerase family protein | CHLREDRAFT_183823 | CYN22 | estExt_fgenesh2_kg.C_180046 | 2.58E-02 | -2.72E+00 |
| Locus_1774_6Transcript_1/1_Confidence_1.000_Length_2074 | Cre12.g533550.t1.1 | Pyruvate kinase family protein | CHLREDRAFT_136854 | PYK1 | estExt_gwp_1W.C_180027 | 5.41E-05 | 2.24E+00 |
| Locus_24923_10Transcript_1/1_Confidence_1.000_Length_666 | Cre12.g530300.t1.1 | FKBP-like peptidyl-prolyl cis-trans isomerase family protein | CHLREDRAFT_79446 | FKB16-3 | estExt_GenewiseW_1.C_180177 | 1.72E-12 | -7.10E+00 |
| Locus_2130_1Transcript_1/2_Confidence_0.667_Length_1919 | Cre12.g530000.t1.1 | MAP kinase 5 | CHLREDRAFT_426547 | ? | pasa_Sanger_mRNA9196 | 2.69E-02 | 4.55E+00 |
| Locus_4885_5Transcript_1/1_Confidence_1.000_Length_4199 | Cre12.g527000.t1.1 | calcium-dependent protein kinase 34 | CHLREDRAFT_346299 | ? | estExt_fgenesh1_pg.C_chromosome_120577 | 2.87E-04 | -4.00E+00 |
| Locus_333_5Transcript_2/2_Confidence_0.875_Length_1320 | Cre12.g524750.t1.1 | NA | CHLREDRAFT_150727 | CHLREDRAFT_150727 | Chlre2_kg.scaffold_31000093 | 1.73E-04 | 2.57E+00 |
| Locus_6456_2Transcript_1/1_Confidence_1.000_Length_1864 | Cre12.g524500.t1.1 | Rubisco methyltransferase family protein | CHLREDRAFT_150732 | RMT2 | Chlre2_kg.scaffold_31000098 | 2.16E-02 | -2.39E+00 |
| Locus_17903_9Transcript_1/1_Confidence_1.000_Length_766 | Cre12.g524300.t1.1 | Tetratricopeptide repeat (TPR)-like superfamily protein | CHLREDRAFT_184916 | CGL71 | estExt_fgenesh2_kg.C_310040 | 4.72E-03 | -2.51E+00 |
| Locus_8689_9Transcript_1/1_Confidence_1.000_Length_467 | Cre12.g522600.t1.1 | cytochrome c-2 | CHLREDRAFT_330365 | ? | pg.chromosome_12_#_519 | 2.33E-07 | 4.53E+00 |
| Locus_4170_7Transcript_4/4_Confidence_0.625_Length_1528 | Cre12.g522250.t1.1 | NA | CHLREDRAFT_346258 | ? | estExt_fgenesh1_pg.C_chromosome_120503 | 1.15E-04 | 3.72E+00 |
| Locus_506_6Transcript_6/10_Confidence_0.412_Length_1343 | Cre12.g519200.t1.2 | elongation factor Ts family protein | CHLREDRAFT_195616 | ? | MAY_157237 | 1.33E-03 | -2.19E+00 |
| Locus_6566_5Transcript_2/2_Confidence_0.500_Length_1513 | Cre12.g518800.t1.1 | Galactose oxidase/kelch repeat superfamily protein | CHLREDRAFT_150835 | CHLREDRAFT_150835 | Chlre2_kg.scaffold_31000201 | 3.83E-02 | 2.88E+00 |
| Locus_3021_2Transcript_1/1_Confidence_1.000_Length_1397 | Cre12.g517100.t1.1 | Chalcone-flavanone isomerase family protein | CHLREDRAFT_150866 | CHLREDRAFT_150866 | Chlre2_kg.scaffold_31000232 | 1.65E-02 | -2.65E+00 |
| Locus_797_6Transcript_1/1_Confidence_1.000_Length_1463 | Cre12.g515850.t1.1 | proliferating cellular nuclear antigen 1 | CHLREDRAFT_140580 | PCN1 | estExt_gwp_1W.C_890009 | 1.79E-02 | -2.92E+00 |
| Locus_481_6Transcript_1/1_Confidence_1.000_Length_1679 | Cre12.g515250.t1.2 | NA | CHLREDRAFT_423707 | ? | pasa_Sanger_mRNA7117 | 8.32E-03 | -4.02E+00 |
| Locus_6204_8Transcript_1/1_Confidence_1.000_Length_2908 | Cre12.g514850.t1.1 | Chaperone protein htpG family protein | CHLREDRAFT_154398 | HSP90C | Chlre2_kg.scaffold_83000033 | 8.04E-03 | -2.52E+00 |
| Locus_5117_7Transcript_1/1_Confidence_1.000_Length_3018 | Cre12.g514200.t1.1 | Glucose-methanol-choline (GMC) oxidoreductase family protein | CHLREDRAFT_424536 | ? | pasa_Sanger_mRNA8856 | 1.17E-07 | -1.23E+01 |
| Locus_4383_10Transcript_1/1_Confidence_1.000_Length_1555 | Cre12.g512950.t1.1 | NA | CHLREDRAFT_180097 | CHLREDRAFT_180097 | fgenesh2_pg.C_scaffold_83000001 | 1.30E-05 | -7.01E+00 |
| Locus_25620_8Transcript_1/1_Confidence_1.000_Length_488 | Cre12.g511800.t1.2 | NA | CHLREDRAFT_188695 | CHLREDRAFT_188695 | estExt_fgenesh2_pg.C_110234 | 1.05E-02 | -5.01E+00 |
| Locus_6453_8Transcript_1/3_Confidence_0.714_Length_1495 | Cre12.g511150.t1.1 | NAD-dependent glycerol-3-phosphate dehydrogenase family protein | CHLREDRAFT_94229 | GPD1 | fgenesh1_est.C_scaffold_11000056 | 2.65E-02 | -3.46E+00 |
| Locus_1069_6Transcript_1/1_Confidence_1.000_Length_1813 | Cre12.g510800.t1.1 | P-loop containing nucleoside triphosphate hydrolases superfamily protein | CHLREDRAFT_135584 | CHLI2 | estExt_gwp_1W.C_110312 | 1.28E-05 | -3.51E+00 |
| Locus_293_4Transcript_1/1_Confidence_1.000_Length_1763 | Cre12.g510050.t1.1 | dicarboxylate diiron protein, putative (Crd1) | CHLREDRAFT_183048 | ? | estExt_fgenesh2_kg.C_110066 | 7.55E-03 | -5.79E+00 |
| Locus_10863_5Transcript_2/4_Confidence_0.727_Length_962 | Cre12.g508850.t1.1 | dehydroascorbate reductase 1 | CHLREDRAFT_188638 | CHLREDRAFT_188638 | estExt_fgenesh2_pg.C_110163 | 4.70E-05 | -9.30E+00 |
| Locus_694_7Transcript_4/4_Confidence_0.667_Length_1094 | Cre12.g508750.t1.1 | photosystem I light harvesting complex gene 6 | CHLREDRAFT_144609 | LHCA2 | Chlre2_kg.scaffold_11000154 | 1.57E-02 | -5.54E+00 |
| Locus_9194_9Transcript_1/1_Confidence_1.000_Length_1424 | Cre12.g507300.t1.1 | Ribosomal protein S8e family protein | CHLREDRAFT_135480 | LCI30 | estExt_gwp_1W.C_110120 | 4.16E-09 | 2.40E+00 |
| Locus_235_4Transcript_3/6_Confidence_0.583_Length_403 | Cre12.g505450.t1.1 | Histone superfamily protein | CHLREDRAFT_295225 | ? | au.g12929_t1 | 3.19E-03 | -2.72E+00 |
| Locus_4588_5Transcript_1/1_Confidence_1.000_Length_752 | Cre12.g503550.t1.1 | isoprenoid F | CHLREDRAFT_188593 | CHLREDRAFT_188593 | estExt_fgenesh2_pg.C_110071 | 2.48E-02 | -2.20E+00 |
| Locus_3515_4Transcript_1/1_Confidence_1.000_Length_674 | Cre12.g502250.t1.1 | Plastid-lipid associated protein PAP / fibrillin family protein | CHLREDRAFT_205639 | PLAP4 | SKA_Chlre2_kg.scaffold_11000050 | 4.43E-07 | 4.78E+00 |
| Locus_6649_7Transcript_1/1_Confidence_1.000_Length_402 | Cre12.g501550.t1.1 | NA | CHLREDRAFT_182999 | CHLREDRAFT_182999 | estExt_fgenesh2_kg.C_110014 | 5.16E-03 | -3.71E+00 |
| Locus_1217_4Transcript_1/1_Confidence_1.000_Length_1200 | Cre12.g498550.t1.2 | magnesium-protoporphyrin IX methyltransferase | CHLREDRAFT_195575 | CHLM | RWI_chlre3.78.3.1.1 | 3.30E-11 | -4.26E+00 |
| Locus_23514_10Transcript_1/1_Confidence_1.000_Length_1374 | Cre12.g497300.t1.1 | calcium sensing receptor | CHLREDRAFT_194676 | TEF2 | estExt_fgenesh2_pg.C_780055 | 3.48E-03 | -8.90E+00 |
| Locus_1425_1Transcript_1/1_Confidence_1.000_Length_1103 | Cre12.g494850.t1.1 | adenosine monophosphate kinase | CHLREDRAFT_194134 | ADK3 | estExt_fgenesh2_pg.C_560023 | 1.47E-02 | -2.26E+00 |
| Locus_5175_5Transcript_1/2_Confidence_0.500_Length_503 | Cre12.g494450.t1.1 | small subunit ribosomal protein 16 | CHLREDRAFT_195629 | PRPS16 | MAY_CACW7214.fwd | 3.81E-05 | -2.87E+00 |
| Locus_10133_1Transcript_1/1_Confidence_1.000_Length_1898 | Cre12.g491050.t1.1 | ribonucleotide reductase 2A | CHLREDRAFT_188785 | RIR2A | estExt_fgenesh2_pg.C_110358 | 1.55E-11 | 9.98E+00 |
| Locus_8390_7Transcript_1/1_Confidence_1.000_Length_1177 | Cre12.g490500.t1.1 | NA | CHLREDRAFT_183100 | ? | estExt_fgenesh2_kg.C_110121 | 1.31E-14 | -1.11E+01 |
| Locus_477_4Transcript_1/1_Confidence_1.000_Length_1065 | Cre12.g486300.t1.1 | photosystem I subunit l | CHLREDRAFT_205935 | PSAL | SKA_Chlre2_kg.scaffold_11000249 | 1.49E-18 | -3.28E+00 |
| Locus_96_6Transcript_1/1_Confidence_1.000_Length_2885 | Cre12.g485800.t1.1 | FtsH extracellular protease family | CHLREDRAFT_55270 | FTSH1 | estExt_GenewiseH_1.C_110008 | 3.25E-02 | -2.59E+00 |
| Locus_6333_4Transcript_1/1_Confidence_1.000_Length_1596 | Cre12.g485150.t1.1 | glyceraldehyde-3-phosphate dehydrogenase of plastid 1 | CHLREDRAFT_140618 | GAP1a | estExt_gwp_1W.C_880024 | 6.96E-03 | 1.28E+03 |
| Locus_2576_1Transcript_1/1_Confidence_1.000_Length_1955 | Cre12.g484000.t1.1 | acetyl-CoA carboxylase carboxyl transferase subunit beta | CHLREDRAFT_133238 | CHLREDRAFT_133238 | estExt_gwp_1H.C_880027 | 5.44E-04 | -3.44E+00 |
| Locus_1551_4Transcript_1/1_Confidence_1.000_Length_1134 | Cre12.g483950.t1.1 | Lactate/malate dehydrogenase family protein | CHLREDRAFT_60444 | MDH4 | estExt_GenewiseH_1.C_880013 | 5.54E-06 | -2.31E+00 |
| Locus_1501_10Transcript_1/2_Confidence_0.750_Length_1049 | Cre11.g481450.t1.1 | ATPase, F0 complex, subunit B/B\', bacterial/chloroplast | CHLREDRAFT_206190 | ATPG | FER_Chlre2_kg.scaffold_33000094 | 3.66E-12 | -3.74E+00 |
| Locus_6448_7Transcript_1/2_Confidence_0.667_Length_920 | Cre11.g479500.t1.1 | ribosomal protein L4 | CHLREDRAFT_185040 | PRPL4 | estExt_fgenesh2_kg.C_330024 | 7.27E-04 | -2.38E+00 |
| Locus_11_3Transcript_3937/11887_Confidence_1.000_Length_763 | Cre11.g478800.t1.1 | NA | CHLREDRAFT_95661 | CHLREDRAFT_95661 | fgenesh1_est.C_scaffold_33000010 | 6.77E-03 | -2.06E+00 |
| Locus_11270_2Transcript_1/1_Confidence_1.000_Length_863 | Cre11.g472700.t1.1 | phosphatidylglycerolphosphate synthase 2 | CHLREDRAFT_194257 | CHLREDRAFT_194257 | estExt_fgenesh2_pg.C_620048 | 3.93E-02 | -3.71E+00 |
| Locus_1680_4Transcript_1/1_Confidence_1.000_Length_557 | Cre11.g468750.t1.1 | NA | CHLREDRAFT_152648 | CPLD48 | Chlre2_kg.scaffold_48000072 | 1.27E-05 | -5.11E+00 |
| Locus_1196_2Transcript_1/1_Confidence_1.000_Length_1683 | Cre11.g468700.t1.1 | coenzyme F420 hydrogenase family / dehydrogenase, beta subunit family | CHLREDRAFT_394141 | ? | pasa_Sanger_mRNA6025 | 2.75E-03 | 6.68E+00 |
| Locus_3070_2Transcript_1/1_Confidence_1.000_Length_1977 | Cre11.g467700.t1.1 | Uroporphyrinogen decarboxylase | CHLREDRAFT_132194 | UROD1 | estExt_gwp_1H.C_480002 | 4.93E-19 | -1.62E+01 |
| Locus_7895_8Transcript_1/1_Confidence_1.000_Length_1044 | Cre10.g466850.t1.1 | FKBP-like peptidyl-prolyl cis-trans isomerase family protein | CHLREDRAFT_156074 | FKB18 | acegs_kg.scaffold_4000124 | 7.67E-03 | -5.15E+00 |
| Locus_889_8Transcript_2/9_Confidence_0.286_Length_773 | Cre10.g466500.t1.1 | Lactoylglutathione lyase / glyoxalase I family protein | CHLREDRAFT_143257 | CHLREDRAFT_143257 | Chlre2_kg.scaffold_4000380 | 7.85E-04 | -2.33E+00 |
| Locus_4792_6Transcript_1/1_Confidence_1.000_Length_4154 | Cre10.g465550.t1.2 | Clp ATPase | CHLREDRAFT_195417 | CLPD1 | OVA_CrClpD | 5.50E-09 | 1.13E+01 |
| Locus_1963_3Transcript_1/1_Confidence_1.000_Length_2592 | Cre10.g461050.t1.1 | vacuolar ATP synthase subunit A | CHLREDRAFT_54608 | ATPvA1 | estExt_GenewiseH_1.C_40224 | 1.96E-02 | -2.15E+00 |
| Locus_5075_8Transcript_2/2_Confidence_0.667_Length_1221 | Cre10.g460050.t1.2 | Tetratricopeptide repeat (TPR)-like superfamily protein | CHLREDRAFT_127367 | ? | estExt_gwp_1H.C_40294 | 1.25E-08 | -5.85E+00 |
| Locus_846_4Transcript_1/1_Confidence_1.000_Length_980 | Cre10.g458550.t1.1 | Uncharacterised BCR, YbaB family COG0718 | CHLREDRAFT_54576 | CHLREDRAFT_54576 | estExt_GenewiseH_1.C_40167 | 8.61E-05 | -2.90E+00 |
| Locus_2339_8Transcript_1/2_Confidence_0.667_Length_989 | Cre10.g456750.t1.1 | dehydroascorbate reductase 2 | CHLREDRAFT_143082 | CHLREDRAFT_143082 | Chlre2_kg.scaffold_4000205 | 6.80E-12 | -3.16E+00 |
| Locus_2661_5Transcript_1/1_Confidence_1.000_Length_1251 | Cre10.g456250.t1.1 | Thioredoxin superfamily protein | CHLREDRAFT_195752 | CHLREDRAFT_195752 | STE_estExt_fgenesh2_kg.C_40067 | 2.17E-06 | 4.55E+00 |
| Locus_2936_4Transcript_1/1_Confidence_1.000_Length_529 | Cre10.g453450.t1.1 | Small nuclear ribonucleoprotein family protein | CHLREDRAFT_182571 | SMP4 | estExt_fgenesh2_kg.C_40055 | 3.17E-02 | -2.08E+00 |
| Locus_151_4Transcript_1/2_Confidence_0.750_Length_1023 | Cre10.g452100.t1.1 | photosystem II BY | CHLREDRAFT_182560 | PSBY1 | estExt_fgenesh2_kg.C_40044 | 3.37E-03 | -9.74E+00 |
| Locus_8917_5Transcript_1/1_Confidence_1.000_Length_2198 | Cre10.g451950.t1.1 | glutamate:glyoxylate aminotransferase | CHLREDRAFT_206184 | AAT1 | FER_estExt_gwp_1H.C_40110 | 5.08E-04 | -5.35E+00 |
| Locus_982_1Transcript_1/1_Confidence_1.000_Length_663 | Cre10.g449550.t1.1 | Thioredoxin superfamily protein | CHLREDRAFT_196639 | PRX3 | STE_fgenesh1_pm.C_scaffold_4000003 | 5.02E-05 | -2.04E+00 |
| Locus_8778_5Transcript_1/1_Confidence_1.000_Length_1038 | Cre10.g446350.t1.2 | Photosystem II reaction center PsbP family protein | CHLREDRAFT_205923 | CGLD14 | SKA_fgenesh2_pg.C_scaffold_4000024 | 7.53E-05 | -7.21E+00 |
| Locus_1379_4Transcript_1/1_Confidence_1.000_Length_708 | Cre10.g444500.t1.1 | sulfurtransferase protein 16 | CHLREDRAFT_311866 | ? | kg.chromosome_10_#_855_#_859505:3 | 1.15E-05 | -2.82E+00 |
| Locus_5186_2Transcript_1/1_Confidence_1.000_Length_1000 | Cre10.g440700.t1.2 | Pyridoxamine 5\'-phosphate oxidase family protein | CHLREDRAFT_281865 | ? | au.g456_t1 | 5.30E-19 | -5.94E+00 |
| Locus_1163_4Transcript_1/1_Confidence_1.000_Length_676 | Cre10.g440450.t1.1 | photosystem II reaction center PSB28 protein | CHLREDRAFT_182896 | PSB28 | estExt_fgenesh2_kg.C_100038 | 7.57E-04 | -2.63E+00 |
| Locus_4658_3Transcript_1/1_Confidence_1.000_Length_1385 | Cre10.g440050.t1.1 | chloroplast stem-loop binding protein of 41 kDa | CHLREDRAFT_205568 | CSP41a | SKA_chlre3.10.18.1.1 | 4.96E-04 | -6.05E+00 |
| Locus_4529_6Transcript_1/1_Confidence_1.000_Length_424 | Cre10.g438550.t1.1 | Bacterial sec-independent translocation protein mttA/Hcf106 | CHLREDRAFT_182920 | TATA | estExt_fgenesh2_kg.C_100062 | 2.15E-06 | -2.90E+00 |
| Locus_2144_6Transcript_1/1_Confidence_1.000_Length_1227 | Cre10.g435850.t1.1 | NA | CHLREDRAFT_182934 | CPLD24 | estExt_fgenesh2_kg.C_100078 | 2.11E-06 | -2.34E+01 |
| Locus_91_3Transcript_1/1_Confidence_1.000_Length_1618 | Cre10.g435800.t1.1 | chloroplast RNA binding | CHLREDRAFT_135322 | CSP41b | estExt_gwp_1W.C_100141 | 4.70E-06 | -8.51E+00 |
| Locus_923_6Transcript_3/5_Confidence_0.538_Length_1619 | Cre10.g434450.t1.1 | NAD(P)-binding Rossmann-fold superfamily protein | CHLREDRAFT_186185 | NUOA9 | estExt_fgenesh2_pg.C_80017 | 9.99E-07 | 2.04E+00 |
| Locus_1456_8Transcript_1/1_Confidence_1.000_Length_895 | Cre10.g433950.t1.2 | NA | CHLREDRAFT_140850 | CHLREDRAFT_140850 | Chlre2_kg.scaffold_8000021 | 1.68E-10 | -2.24E+00 |
| Locus_4078_7Transcript_1/1_Confidence_1.000_Length_2476 | Cre10.g433600.t1.1 | methylenetetrahydrofolate reductase 2 | CHLREDRAFT_111330 | CHLREDRAFT_111330 | e_gwW.8.191.1 | 4.98E-02 | -2.14E+00 |
| Locus_18582_2Transcript_1/1_Confidence_1.000_Length_1283 | Cre10.g426350.t1.1 | NA | CHLREDRAFT_126036 | CHLREDRAFT_126036 | estExt_gwp_1H.C_80019 | 3.20E-04 | -8.16E+00 |
| Locus_5604_2Transcript_2/2_Confidence_0.417_Length_829 | Cre10.g425650.t1.1 | NA | CHLREDRAFT_281561 | ? | au.g165_t1 | 2.31E-02 | -3.62E+00 |
| Locus_3525_2Transcript_1/1_Confidence_1.000_Length_3582 | Cre10.g424750.t1.1 | pyruvate orthophosphate dikinase | CHLREDRAFT_196616 | PPD2 | MHS_estExt_gwp_1H.C_180145 | 1.52E-08 | 4.07E+00 |
| Locus_29_5Transcript_4/4_Confidence_0.692_Length_1122 | Cre10.g424100.t1.1 | pyrophosphorylase 6 | CHLREDRAFT_133620 | IPY1 | estExt_gwp_1W.C_80253 | 1.06E-02 | -8.07E+00 |
| Locus_12158_8Transcript_1/1_Confidence_1.000_Length_948 | Cre10.g423650.t1.1 | plastid ribosomal protein l11 | CHLREDRAFT_195634 | PRPL11 | MAY_161549 | 3.51E-05 | -2.95E+00 |
| Locus_3379_9Transcript_1/1_Confidence_1.000_Length_1255 | Cre10.g423500.t1.1 | heme oxygenase 3 | CHLREDRAFT_195947 | HMOX1 | RWI_chlre3.8.80.1.1 | 8.79E-12 | -3.06E+00 |
| Locus_5756_1Transcript_3/3_Confidence_0.778_Length_750 | Cre10.g422300.t1.1 | Thioredoxin superfamily protein | CHLREDRAFT_181913 | PRX6 | estExt_fgenesh2_kg.C_80081 | 1.13E-07 | -3.40E+00 |
| Locus_12024_10Transcript_3/3_Confidence_0.750_Length_1318 | Cre09.g416350.t1.1 | nucleosome assembly protein1;1 | CHLREDRAFT_192162 | CHLREDRAFT_192162 | estExt_fgenesh2_pg.C_300166 | 3.79E-03 | 2.24E+00 |
| Locus_684_3Transcript_2/5_Confidence_0.455_Length_1386 | Cre09.g415700.t1.1 | alpha carbonic anhydrase 7 | CHLREDRAFT_206201 | CAH3 | OVA_fgenesh2_kg.C_scaffold_30000048 | 8.84E-06 | -5.28E+00 |
| Locus_1149_1Transcript_1/1_Confidence_1.000_Length_2749 | Cre09.g411900.t1.2 | serine hydroxymethyltransferase 3 | CHLREDRAFT_196400 | SHMT3 | BTM_estExt_gwp_1W.C_300088 | 7.84E-09 | 2.94E+00 |
| Locus_1337_4Transcript_1/1_Confidence_1.000_Length_867 | Cre09.g411200.t1.1 | Rieske (2Fe-2S) domain-containing protein | CHLREDRAFT_192099 | TEF5 | estExt_fgenesh2_pg.C_300071 | 6.79E-07 | -6.30E+00 |
| Locus_979_2Transcript_1/2_Confidence_0.833_Length_2662 | Cre09.g410850.t1.1 | nitrate transporter2.5 | CHLREDRAFT_345325 | ? | estExt_fgenesh1_pg.C_chromosome_90357 | 1.15E-04 | -4.48E+00 |
| Locus_615_3Transcript_13/13_Confidence_0.740_Length_2360 | Cre09.g410800.t1.1 | nitrate transporter2.5 | CHLREDRAFT_192088 | NAR4 | estExt_fgenesh2_pg.C_300059 | 3.29E-08 | -5.48E+00 |
| Locus_591_3Transcript_4/7_Confidence_0.727_Length_5170 | Cre09.g410750.t1.1 | nitrite reductase 1 | CHLREDRAFT_192085 | NII1 | estExt_fgenesh2_pg.C_300056 | 1.14E-04 | -1.35E+01 |
| Locus_6427_7Transcript_1/1_Confidence_1.000_Length_1141 | Cre09.g409100.t1.1 | uroporphyrinogen-III synthase family protein | CHLREDRAFT_195943 | UROS | RWI_chlre3.30.98.1.1 | 2.45E-08 | -5.74E+00 |
| Locus_3291_8Transcript_1/1_Confidence_1.000_Length_1148 | Cre09.g405800.t1.1 | Cystathionine beta-synthase (CBS) family protein | CHLREDRAFT_185012 | CHLREDRAFT_185012 | estExt_fgenesh2_kg.C_350018 | 1.34E-07 | -3.96E+00 |
| Locus_2411_4Transcript_1/1_Confidence_1.000_Length_1556 | Cre09.g405500.t1.2 | NA | CHLREDRAFT_338214 | ? | pm.chromosome_9_#_225 | 3.42E-48 | 5.91E+00 |
| Locus_11964_8Transcript_1/1_Confidence_1.000_Length_1010 | Cre09.g404000.t1.2 | Tetratricopeptide repeat (TPR)-like superfamily protein | CHLREDRAFT_149642 | CHLREDRAFT_149642 | Chlre2_kg.scaffold_25000121 | 5.68E-04 | -3.42E+00 |
| Locus_8535_6Transcript_1/1_Confidence_1.000_Length_1137 | Cre09.g395650.t1.1 | NA | CHLREDRAFT_184175 | CHLREDRAFT_184175 | estExt_fgenesh2_kg.C_220054 | 2.06E-02 | -4.62E+00 |
| Locus_1212_8Transcript_1/1_Confidence_1.000_Length_2500 | Cre09.g394850.t1.1 | membrane protein, putative | CHLREDRAFT_148310 | TEF24 | Chlre2_kg.scaffold_22000148 | 3.68E-05 | 4.23E+00 |
| Locus_4755_8Transcript_1/1_Confidence_1.000_Length_2085 | Cre09.g394750.t1.1 | ribosomal protein S1 | CHLREDRAFT_79955 | PRPS1 | estExt_GenewiseW_1.C_220152 | 2.73E-07 | -3.55E+00 |
| Locus_319_9Transcript_1/1_Confidence_1.000_Length_4262 | Cre09.g393150.t1.1 | NA | CHLREDRAFT_184156 | FOX1 | estExt_fgenesh2_kg.C_220035 | 1.50E-04 | -5.62E+02 |
| Locus_875_4Transcript_1/1_Confidence_1.000_Length_486 | Cre09.g392350.t1.1 | glycine-rich RNA-binding protein 2 | CHLREDRAFT_184151 | CHLREDRAFT_184151 | estExt_fgenesh2_kg.C_220030 | 9.66E-12 | 4.68E+00 |
| Locus_5513_6Transcript_1/1_Confidence_1.000_Length_861 | Cre09.g389700.t1.1 | NA | CHLREDRAFT_148227 | CHLREDRAFT_148227 | Chlre2_kg.scaffold_22000065 | 4.10E-02 | -6.16E+00 |
| Locus_1470_7Transcript_5/5_Confidence_0.600_Length_1131 | Cre09.g387800.t1.1 | ferritin 3 | CHLREDRAFT_206372 | FER1 | SKA_fgenesh2_kg.C_scaffold_22000005 | 7.27E-24 | 4.80E+00 |
| Locus_2897_7Transcript_1/8_Confidence_0.526_Length_1510 | Cre09.g387450.t1.2 | NA | CHLREDRAFT_148176 | CHLREDRAFT_148176 | Chlre2_kg.scaffold_22000014 | 7.72E-03 | 2.96E+00 |
| Locus_5029_3Transcript_2/10_Confidence_0.273_Length_623 | Cre09.g387450.t1.2 | NA | CHLREDRAFT_94992 | CHLREDRAFT_94992 | fgenesh1_est.C_scaffold_22000004 | 6.03E-04 | 5.72E+00 |
| Locus_5196_10Transcript_2/4_Confidence_0.500_Length_1576 | Cre09.g387450.t1.2 | NA | CHLREDRAFT_148177 | CHLREDRAFT_148177 | Chlre2_kg.scaffold_22000015 | 2.49E-05 | 3.55E+00 |
| Locus_308_1Transcript_1/1_Confidence_1.000_Length_2598 | Cre09.g387050.t1.1 | pfkB-like carbohydrate kinase family protein | CHLREDRAFT_205628 | CHLREDRAFT_205628 | SKA_estExt_fgenesh2_pg.C_220006 | 2.83E-02 | 2.83E+00 |
| Locus_190_1Transcript_3/3_Confidence_0.714_Length_1464 | Cre09.g386650.t1.1 | ADP/ATP carrier 3 | CHLREDRAFT_196520 | ANT1 | JLM_estExt_gwp_1W.C_250131 | 1.12E-14 | 2.24E+00 |
| Locus_3262_8Transcript_1/1_Confidence_1.000_Length_751 | Cre08.g385200.t1.1 | Small nuclear ribonucleoprotein family protein | CHLREDRAFT_58042 | LSM5 | estExt_GenewiseH_1.C_270127 | 1.18E-04 | -2.67E+00 |
| Locus_26894_7Transcript_1/2_Confidence_0.667_Length_810 | Cre08.g384650.t1.1 | Chlorophyll A-B binding family protein | CHLREDRAFT_148418 | CHLREDRAFT_148418 | Chlre2_kg.scaffold_21000031 | 3.28E-02 | -3.01E+00 |
| Locus_15041_8Transcript_1/1_Confidence_1.000_Length_869 | Cre08.g382450.t1.1 | NA | CHLREDRAFT_148452 | CHLREDRAFT_148452 | Chlre2_kg.scaffold_21000065 | 1.55E-02 | 6.65E+00 |
| Locus_1744_2Transcript_2/2_Confidence_0.833_Length_1513 | Cre08.g380600.t1.2 | FUS3-complementing gene 2 | CHLREDRAFT_391337 | ? | pasa_Sanger_mRNA31450 | 1.50E-04 | 3.84E+00 |
| Locus_238_2Transcript_2/2_Confidence_0.750_Length_1640 | Cre08.g379350.t1.2 | Glucose-6-phosphate/phosphate translocator-related | CHLREDRAFT_191135 | TPT1 | estExt_fgenesh2_pg.C_210104 | 2.27E-03 | 2.40E+01 |
| Locus_4362_1Transcript_1/1_Confidence_1.000_Length_1826 | Cre08.g378850.t1.1 | Phosphoribosyltransferase family protein | CHLREDRAFT_137493 | RPPK1 | estExt_gwp_1W.C_210026 | 8.79E-10 | 4.06E+00 |
| Locus_1569_2Transcript_2/4_Confidence_0.143_Length_784 | Cre08.g378150.t1.2 | glucose-6-phosphate dehydrogenase 3 | CHLREDRAFT_173841 | GLD2-N | fgenesh2_pg.C_scaffold_21000136 | 8.93E-03 | 2.23E+00 |
| Locus_1738_6Transcript_1/1_Confidence_1.000_Length_936 | Cre08.g377550.t1.1 | Yippee family putative zinc-binding protein | CHLREDRAFT_309888 | ? | kg.chromosome_8_#_401_#_TC40911 | 3.13E-04 | 2.82E+00 |
| Locus_1150_6Transcript_1/1_Confidence_1.000_Length_4066 | Cre08.g375500.t1.1 | glutamine-fructose-6-phosphate transaminase (isomerizing)s;sugar binding;transaminases | CHLREDRAFT_118046 | ATF1 | e_gwW.21.159.1 | 1.16E-02 | 2.11E+00 |
| Locus_595_2Transcript_1/1_Confidence_1.000_Length_2104 | Cre08.g372950.t1.1 | 4-hydroxy-3-methylbut-2-enyl diphosphate reductase | CHLREDRAFT_59822 | IDS1 | estExt_GenewiseH_1.C_640019 | 7.37E-04 | -2.19E+00 |
| Locus_443_5Transcript_1/5_Confidence_0.333_Length_1015 | Cre08.g372450.t1.1 | photosystem II subunit Q-2 | CHLREDRAFT_153656 | PSBQ | Chlre2_kg.scaffold_64000041 | 1.30E-03 | -2.30E+01 |
| Locus_17270_5Transcript_1/1_Confidence_1.000_Length_615 | Cre08.g371650.t1.1 | Bacterial sec-independent translocation protein mttA/Hcf106 | CHLREDRAFT_20727 | TATB | fgenesh1_pg.C_scaffold_64000030 | 2.22E-03 | -8.10E+00 |
| Locus_4942_7Transcript_4/4_Confidence_0.571_Length_1863 | Cre08.g367600.t1.1 | O-acetylserine (thiol) lyase isoform C | CHLREDRAFT_175651 | OASTL1 | fgenesh2_pg.C_scaffold_29000124 | 2.75E-03 | 3.64E+00 |
| Locus_27397_1Transcript_1/1_Confidence_1.000_Length_521 | Cre08.g367550.t1.1 | NA | CHLREDRAFT_184735 | FAP291 | estExt_fgenesh2_kg.C_290032 | 1.51E-03 | 1.52E+01 |
| Locus_614_4Transcript_1/1_Confidence_1.000_Length_2561 | Cre08.g365700.t1.1 | sulfite reductase | CHLREDRAFT_206154 | SIR1 | SKA_estExt_gwp_1H.C_460014 | 3.17E-14 | 3.34E+00 |
| Locus_8534_7Transcript_1/1_Confidence_1.000_Length_786 | Cre08.g365650.t1.1 | Protein phosphatase 2C family protein | CHLREDRAFT_391387 | ? | pasa_Sanger_mRNA31084 | 2.20E-06 | -4.35E+00 |
| Locus_7047_10Transcript_1/1_Confidence_1.000_Length_808 | Cre08.g365400.t1.1 | Ribosomal protein L31 | CHLREDRAFT_195624 | PRPL31 | MAY_168249 | 3.64E-08 | -2.69E+00 |
| Locus_7783_3Transcript_2/2_Confidence_0.750_Length_1345 | Cre08.g362900.t1.2 | Photosystem II reaction center PsbP family protein | CHLREDRAFT_205916 | PSBP4 | OVA_e_gwH.29.52.1 | 1.40E-03 | -4.59E+00 |
| Locus_3038_1Transcript_1/7_Confidence_0.267_Length_1420 | Cre08.g360200.t1.1 | solute:sodium symporters;urea transmembrane transporters | CHLREDRAFT_154212 | DUR3B | Chlre2_kg.scaffold_77000030 | 1.07E-45 | 5.20E+01 |
| Locus_710_10Transcript_4/7_Confidence_0.405_Length_2500 | Cre08.g360100.t1.1 | translocon at the outer membrane of chloroplasts 64-V | CHLREDRAFT_196482 | DUR2 | SKA_Chlre2_kg.scaffold_77000027 | 9.59E-10 | 3.49E+00 |
| Locus_5144_9Transcript_1/2_Confidence_0.875_Length_4596 | Cre08.g360050.t1.2 | acetyl Co-enzyme a carboxylase biotin carboxylase subunit | CHLREDRAFT_133000 | DUR1 | estExt_gwp_1H.C_770032 | 1.10E-22 | 1.62E+01 |
| Locus_1026_3Transcript_1/1_Confidence_1.000_Length_2551 | Cre08.g359350.t1.1 | acetyl Co-enzyme a carboxylase biotin carboxylase subunit | CHLREDRAFT_122970 | CHLREDRAFT_122970 | e_gwW.77.4.1 | 2.26E-03 | -2.45E+00 |
| Locus_2406_2Transcript_5/9_Confidence_0.522_Length_1918 | Cre08.g359300.t1.1 | NA | CHLREDRAFT_196484 | PHO1 | JLM_estExt_fgenesh2_pg.C_770014 | 7.36E-04 | -1.50E+02 |
| Locus_4695_8Transcript_2/3_Confidence_0.688_Length_1482 | Cre08.g358250.t1.1 | Pentatricopeptide repeat (PPR) superfamily protein | CHLREDRAFT_205934 | MCA1 | SKA_chlre3.75.14.1.1 | 2.38E-13 | -1.90E+01 |
| Locus_2829_7Transcript_1/1_Confidence_1.000_Length_2995 | Cre07.g356350.t1.1 | Deoxyxylulose-5-phosphate synthase | CHLREDRAFT_196568 | DXS1 | LOH_estExt_fgenesh2_kg.C_710008 | 3.01E-10 | -4.26E+00 |
| Locus_4680_9Transcript_1/1_Confidence_1.000_Length_1644 | Cre07.g355600.t1.1 | NADPH-dependent thioredoxin reductase A | CHLREDRAFT_132865 | NTR3 | estExt_gwp_1H.C_710043 | 4.99E-02 | -2.09E+00 |
| Locus_725_4Transcript_1/1_Confidence_1.000_Length_451 | Cre07.g355500.t1.1 | Yippee family putative zinc-binding protein | CHLREDRAFT_296922 | ? | au.g14541_t1 | 4.88E-08 | 2.98E+00 |
| Locus_1992_6Transcript_1/1_Confidence_1.000_Length_1983 | Cre07.g350750.t1.1 | Alternative oxidase family protein | CHLREDRAFT_189624 | PTOX1 | estExt_fgenesh2_pg.C_160206 | 6.93E-05 | -3.28E+00 |
| Locus_38493_9Transcript_1/1_Confidence_1.000_Length_1329 | Cre07.g348450.t1.2 | calcium-dependent protein kinase 24 | CHLREDRAFT_391789 | ? | pasa_Sanger_mRNA30679 | 5.61E-04 | 8.07E+00 |
| Locus_36_4Transcript_1/1_Confidence_1.000_Length_1049 | Cre07.g344950.t1.1 | photosystem I light harvesting complex gene 5 | CHLREDRAFT_136294 | LHCA9 | estExt_gwp_1W.C_160133 | 5.03E-04 | -5.31E+00 |
| Locus_1053_1Transcript_1/1_Confidence_1.000_Length_2148 | Cre07.g344600.t1.1 | D-3-phosphoglycerate dehydrogenase | CHLREDRAFT_78757 | PGD1 | estExt_GenewiseW_1.C_160004 | 3.51E-11 | 3.59E+00 |
| Locus_1260_2Transcript_1/1_Confidence_1.000_Length_2612 | Cre07.g342150.t1.1 | Glutamyl-tRNA reductase family protein | CHLREDRAFT_183460 | HEMA | estExt_fgenesh2_kg.C_160028 | 1.23E-12 | -4.43E+00 |
| Locus_4740_1Transcript_1/2_Confidence_0.600_Length_948 | Cre07.g339300.t1.1 | Zim17-type zinc finger protein | CHLREDRAFT_296580 | ? | au.g14223_t1 | 4.47E-03 | 2.39E+00 |
| Locus_3914_2Transcript_3/3_Confidence_0.714_Length_1828 | Cre07.g338450.t1.2 | NA | CHLREDRAFT_159574 | ? | acegs_kg.scaffold_50000002 | 1.74E-03 | 3.03E+00 |
| Locus_967_4Transcript_1/1_Confidence_1.000_Length_2971 | Cre07.g336950.t1.1 | alpha-glucan phosphorylase 2 | CHLREDRAFT_152708 | PHOA | Chlre2_kg.scaffold_50000042 | 1.56E-58 | 9.24E+00 |
| Locus_894_7Transcript_11/11_Confidence_0.429_Length_1685 | Cre07.g335600.t1.1 | NA | CHLREDRAFT_132235 | NAR1.4 | estExt_gwp_1H.C_500017 | 1.70E-16 | 1.42E+01 |
| Locus_9867_7Transcript_1/1_Confidence_1.000_Length_670 | Cre07.g334550.t1.1 | photosystem I subunit O | CHLREDRAFT_193847 | PSAO | estExt_fgenesh2_pg.C_500079 | 1.86E-03 | -6.14E+00 |
| Locus_11020_3Transcript_1/1_Confidence_1.000_Length_1812 | Cre07.g331800.t1.1 | pfkB-like carbohydrate kinase family protein | CHLREDRAFT_169453 | CGL79 | fgenesh2_pg.C_scaffold_10000255 | 1.50E-02 | 3.04E+00 |
| Locus_35636_9Transcript_1/1_Confidence_1.000_Length_684 | Cre07.g331750.t1.1 | NA | CHLREDRAFT_161860 | CHLREDRAFT_161860 | fgenesh2_kg.C_scaffold_10000097 | 1.43E-02 | 2.14E+00 |
| Locus_2389_1Transcript_1/1_Confidence_1.000_Length_1772 | Cre07.g331550.t1.1 | phosphoserine aminotransferase | CHLREDRAFT_188476 | PST1 | estExt_fgenesh2_pg.C_100256 | 5.15E-07 | 2.52E+00 |
| Locus_258_5Transcript_1/1_Confidence_1.000_Length_946 | Cre07.g330250.t1.1 | photosystem I subunit H-1 | CHLREDRAFT_182959 | PSAH | estExt_fgenesh2_kg.C_100106 | 3.05E-02 | -4.80E+00 |
| Locus_4392_5Transcript_2/2_Confidence_0.800_Length_2535 | Cre07.g330100.t1.1 | staurosporin and temperature sensitive 3-like b | CHLREDRAFT_127991 | GTR25 | estExt_gwp_1H.C_100286 | 2.23E-02 | -2.49E+00 |
| Locus_551_6Transcript_1/5_Confidence_0.429_Length_1844 | Cre07.g329500.t1.1 | SITE-1 protease | CHLREDRAFT_205991 | SUB1 | OVA_Chlre2_kg.scaffold_10000257 | 4.56E-05 | -4.60E+00 |
| Locus_907_5Transcript_1/1_Confidence_1.000_Length_1185 | Cre07.g327100.t1.1 | NA | CHLREDRAFT_296325 | ? | au.g13980_t1 | 1.56E-02 | -3.62E+00 |
| Locus_530_9Transcript_1/2_Confidence_0.750_Length_3621 | Cre07.g325500.t1.1 | magnesium-chelatase subunit chlH, chloroplast, putative / Mg-protoporphyrin IX chelatase, putative (CHLH) | CHLREDRAFT_195524 | CHLH1 | RWI_estExt_fgenesh2_pg.C_60376 | 1.43E-04 | -3.48E+00 |
| Locus_301_4Transcript_1/1_Confidence_1.000_Length_1148 | Cre07.g324200.t1.1 | NA | CHLREDRAFT_77062 | BTA1 | estExt_GenewiseW_1.C_60028 | 4.92E-11 | -4.12E+00 |
| Locus_6228_10Transcript_1/1_Confidence_1.000_Length_2101 | Cre07.g322950.t1.1 | NAD kinase 2 | CHLREDRAFT_196779 | NADK1 | OVA_fgenesh1_pg.C_scaffold_6000376 | 1.38E-02 | 2.76E+00 |
| Locus_32433_9Transcript_1/1_Confidence_1.000_Length_1083 | Cre07.g320350.t1.1 | DNAJ heat shock N-terminal domain-containing protein | CHLREDRAFT_187510 | CDJ5 | estExt_fgenesh2_pg.C_60282 | 6.96E-03 | -5.43E+00 |
| Locus_5076_5Transcript_2/2_Confidence_0.667_Length_675 | Cre07.g318800.t1.1 | HSP20-like chaperones superfamily protein | CHLREDRAFT_134508 | HSP22A | estExt_gwp_1W.C_60086 | 2.89E-04 | 3.21E+00 |
| Locus_8556_8Transcript_1/1_Confidence_1.000_Length_954 | Cre07.g316000.t1.1 | nuclear transport factor 2B | CHLREDRAFT_296085 | ? | au.g13754_t1 | 4.79E-04 | -2.23E+00 |
| Locus_6135_8Transcript_1/1_Confidence_1.000_Length_1157 | Cre07.g315150.t1.1 | Rubredoxin-like superfamily protein | CHLREDRAFT_182463 | RBD1 | estExt_fgenesh2_kg.C_60058 | 5.35E-04 | -2.94E+00 |
| Locus_1603_3Transcript_1/1_Confidence_1.000_Length_1906 | Cre07.g313700.t1.2 | glutamate tRNA synthetase | CHLREDRAFT_195574 | GTS2 | RWI_e_gwW.6.42.1 | 7.23E-07 | -4.83E+00 |
| Locus_3809_9Transcript_1/1_Confidence_1.000_Length_2486 | Cre06.g308500.t1.1 | carbamoyl phosphate synthetase A | CHLREDRAFT_128227 | CMPS1 | estExt_gwp_1H.C_120006 | 1.11E-09 | 2.42E+00 |
| Locus_33148_9Transcript_1/1_Confidence_1.000_Length_625 | Cre06.g307200.t1.1 | cAMP-regulated phosphoprotein 19-related protein | CHLREDRAFT_400898 | ? | pasa_Sanger_mRNA28745 | 2.18E-02 | -2.45E+00 |
| Locus_7552_10Transcript_1/1_Confidence_1.000_Length_1283 | Cre06.g304500.t1.1 | chloroplast signal recognition particle component (CAO) | CHLREDRAFT_144935 | ZYS3-2 | Chlre2_kg.scaffold_12000129 | 1.09E-04 | -2.42E+00 |
| Locus_6215_6Transcript_1/1_Confidence_1.000_Length_1164 | Cre06.g303050.t1.1 | cytochrome B5 isoform D | CHLREDRAFT_188890 | CHLREDRAFT_188890 | estExt_fgenesh2_pg.C_120165 | 2.79E-02 | -6.44E+00 |
| Locus_655_3Transcript_1/1_Confidence_1.000_Length_1015 | Cre06.g300700.t1.1 | NAD(P)-binding Rossmann-fold superfamily protein | CHLREDRAFT_344487 | ? | estExt_fgenesh1_pg.C_chromosome_60719 | 1.74E-15 | -6.91E+00 |
| Locus_37366_9Transcript_1/1_Confidence_1.000_Length_1345 | Cre06.g299800.t1.1 | AMP-dependent synthetase and ligase family protein | CHLREDRAFT_188925 | CHLREDRAFT_188925 | estExt_fgenesh2_pg.C_120219 | 3.25E-02 | 5.55E+00 |
| Locus_7946_2Transcript_1/1_Confidence_1.000_Length_3063 | Cre06.g295700.t1.2 | Minichromosome maintenance (MCM2/3/5) family protein | CHLREDRAFT_115083 | MCM3 | e_gwW.12.15.1 | 5.92E-03 | -3.36E+00 |
| Locus_2255_7Transcript_1/1_Confidence_1.000_Length_1773 | Cre06.g295450.t1.1 | hydroxypyruvate reductase | CHLREDRAFT_128310 | HPR1 | estExt_gwp_1H.C_120143 | 2.28E-03 | -2.38E+00 |
| Locus_4683_2Transcript_1/1_Confidence_1.000_Length_1832 | Cre06.g295400.t1.1 | Mitochondrial substrate carrier family protein | CHLREDRAFT_188977 | MITC11 | estExt_fgenesh2_pg.C_120303 | 1.06E-02 | -4.79E+00 |
| Locus_291_3Transcript_1/1_Confidence_1.000_Length_1816 | Cre06.g294950.t1.2 | NAD(P)-binding Rossmann-fold superfamily protein | CHLREDRAFT_400691 | ? | pasa_Sanger_mRNA26991 | 2.07E-07 | -3.81E+00 |
| Locus_6167_2Transcript_1/1_Confidence_1.000_Length_1580 | Cre06.g294750.t1.1 | UbiA prenyltransferase family protein | CHLREDRAFT_5437 | CHLG | fgenesh1_kg.C_scaffold_69000004 | 8.12E-06 | -3.71E+00 |
| Locus_4402_2Transcript_1/1_Confidence_1.000_Length_2086 | Cre06.g294650.t1.1 | alanine:glyoxylate aminotransferase | CHLREDRAFT_194541 | AGT1 | estExt_fgenesh2_pg.C_690020 | 9.99E-07 | -6.15E+00 |
| Locus_7048_10Transcript_1/1_Confidence_1.000_Length_877 | Cre06.g293600.t1.2 | ATP binding;leucine-tRNA ligases;aminoacyl-tRNA ligases;nucleotide binding;ATP binding;aminoacyl-tRNA ligases | CHLREDRAFT_295585 | ? | au.g13269_t1 | 5.33E-03 | -2.95E+00 |
| Locus_1008_4Transcript_1/1_Confidence_1.000_Length_2104 | Cre06.g293050.t1.2 | ammonium transporter 1;1 | CHLREDRAFT_158745 | AMT1A | acegs_kg.scaffold_34000015 | 1.10E-101 | 7.37E+00 |
| Locus_2112_4Transcript_1/1_Confidence_1.000_Length_781 | Cre06.g292400.t1.2 | Nuclear transport factor 2 (NTF2) family protein | CHLREDRAFT_21100 | SOUL5 | fgenesh1_pg.C_scaffold_69000062 | 1.43E-06 | -2.76E+00 |
| Locus_2762_10Transcript_2/2_Confidence_0.667_Length_1289 | Cre06.g287650.t1.1 | XB3 ortholog 1 in Arabidopsis thaliana | CHLREDRAFT_307659 | ? | kg.chromosome_6_#_1930_#_852777:2 | 4.66E-02 | 3.00E+00 |
| Locus_1785_8Transcript_2/3_Confidence_0.867_Length_491 | Cre06.g285250.t1.1 | photosystem II light harvesting complex gene 2.1 | CHLREDRAFT_184490 | LHCBM6 | estExt_fgenesh2_kg.C_260057 | 3.97E-02 | -2.02E+01 |
| Locus_2385_5Transcript_1/3_Confidence_0.667_Length_1454 | Cre06.g284700.t1.1 | alanine aminotransferase 2 | CHLREDRAFT_191703 | AAT2 | estExt_fgenesh2_pg.C_260132 | 2.07E-02 | 3.80E+00 |
| Locus_863_7Transcript_2/3_Confidence_0.857_Length_606 | Cre06.g284250.t1.1 | photosystem II light harvesting complex gene 2.1 | CHLREDRAFT_205752 | LHCBM8 | SKA_estExt_gwp_1W.C_260170 | 1.12E-03 | -1.51E+01 |
| Locus_28_4Transcript_3/5_Confidence_0.600_Length_1057 | Cre06.g284200.t1.1 | photosystem II light harvesting complex gene 2.1 | CHLREDRAFT_184479 | LHCBM9 | estExt_fgenesh2_kg.C_260046 | 8.79E-07 | -1.77E+01 |
| Locus_1375_7Transcript_5/9_Confidence_0.658_Length_1095 | Cre06.g283950.t1.1 | photosystem II light harvesting complex gene 2.2 | CHLREDRAFT_191690 | LHCBM4 | estExt_fgenesh2_pg.C_260118 | 1.63E-04 | -2.02E+01 |
| Locus_2017_9Transcript_1/1_Confidence_1.000_Length_1200 | Cre06.g283050.t1.1 | photosystem I light harvesting complex gene 1 | CHLREDRAFT_184471 | LHCA1 | estExt_fgenesh2_kg.C_260038 | 7.85E-04 | -1.30E+01 |
| Locus_1373_1Transcript_1/2_Confidence_0.750_Length_1449 | Cre06.g281050.t1.1 | sorting nexin 1 | CHLREDRAFT_191646 | VPS5A | estExt_fgenesh2_pg.C_260062 | 1.65E-02 | 3.47E+00 |
| Locus_775_6Transcript_4/5_Confidence_0.364_Length_2739 | Cre06.g280950.t1.1 | Pyruvate kinase family protein | CHLREDRAFT_196263 | PYK2 | MHS_fgenesh1_pg.C_scaffold_26000067 | 2.17E-03 | 2.94E+00 |
| Locus_14740_10Transcript_1/1_Confidence_1.000_Length_1111 | Cre06.g280650.t1.1 | NA | CHLREDRAFT_158401 | CGL59 | acegs_kg.scaffold_26000029 | 8.72E-06 | -3.37E+00 |
| Locus_2416_3Transcript_1/1_Confidence_1.000_Length_1165 | Cre06.g279850.t1.1 | NAD(P)-binding Rossmann-fold superfamily protein | CHLREDRAFT_196378 | CHLREDRAFT_196378 | SKA_estExt_fgenesh2_kg.C_260014 | 2.26E-03 | -4.39E+00 |
| Locus_2206_4Transcript_1/1_Confidence_1.000_Length_271 | Cre06.g279700.t1.2 | NA | CHLREDRAFT_400443 | ? | pasa_Sanger_mRNA26669 | 4.92E-03 | -2.33E+01 |
| Locus_4307_5Transcript_1/1_Confidence_1.000_Length_1492 | Cre06.g279400.t1.1 | serine carboxypeptidase-like 48 | CHLREDRAFT_402187 | ? | pasa_Sanger_mRNA26657 | 1.05E-06 | 2.40E+00 |
| Locus_4746_7Transcript_1/1_Confidence_1.000_Length_1159 | Cre06.g278750.t1.1 | plant uncoupling mitochondrial protein 1 | CHLREDRAFT_138059 | UCP1 | estExt_gwp_1W.C_260156 | 1.34E-02 | -2.30E+00 |
| Locus_5906_9Transcript_5/5_Confidence_0.588_Length_1472 | Cre06.g275100.t1.1 | nucleolin like 2 | CHLREDRAFT_182256 | ? | estExt_fgenesh2_kg.C_30007 | 1.44E-02 | 2.32E+00 |
| Locus_2832_2Transcript_1/1_Confidence_1.000_Length_1618 | Cre06.g275050.t1.1 | gamma-glutamyl hydrolase 1 | CHLREDRAFT_187002 | GGH2 | estExt_fgenesh2_pg.C_30028 | 8.80E-04 | -4.46E+00 |
| Locus_992_4Transcript_1/1_Confidence_1.000_Length_1249 | Cre06.g273700.t1.1 | photosystem II stability/assembly factor, chloroplast (HCF136) | CHLREDRAFT_112806 | CHLREDRAFT_112806 | e_gwW.3.111.1 | 5.73E-21 | -1.82E+01 |
| Locus_2643_10Transcript_1/2_Confidence_0.667_Length_815 | Cre06.g273000.t1.1 | HIS triad family protein 3 | CHLREDRAFT_167053 | CHLREDRAFT_167053 | fgenesh2_pg.C_scaffold_3000063 | 3.66E-04 | -2.62E+00 |
| Locus_941_6Transcript_1/2_Confidence_0.800_Length_1094 | Cre06.g272850.t1.1 | Ribosomal protein L10 family protein | CHLREDRAFT_126960 | PRPL10 | estExt_gwp_1H.C_30509 | 2.47E-06 | -2.99E+00 |
| Locus_29418_9Transcript_1/1_Confidence_1.000_Length_1144 | Cre06.g272650.t1.1 | photosystem I light harvesting complex gene 5 | CHLREDRAFT_187025 | LHCA8 | estExt_fgenesh2_pg.C_30070 | 5.60E-04 | -1.32E+01 |
| Locus_6_1Transcript_1/1_Confidence_1.000_Length_2199 | Cre06.g272050.t1.1 | Phosphoglycerate mutase, 2,3-bisphosphoglycerate-independent | CHLREDRAFT_161085 | ? | fgenesh2_kg.C_scaffold_3000023 | 4.97E-05 | 2.82E+00 |
| Locus_3089_8Transcript_1/3_Confidence_0.667_Length_1269 | Cre06.g269050.t1.1 | NAD(P)-binding Rossmann-fold superfamily protein | CHLREDRAFT_142153 | YCF39-2 | Chlre2_kg.scaffold_3000126 | 7.31E-16 | -1.31E+01 |
| Locus_3892_2Transcript_1/2_Confidence_0.667_Length_987 | Cre06.g268600.t1.1 | glycine-rich protein 2B | CHLREDRAFT_126810 | NAB1 | estExt_gwp_1H.C_30224 | 2.29E-11 | -1.41E+01 |
| Locus_4258_6Transcript_3/3_Confidence_0.750_Length_2140 | Cre06.g267600.t1.1 | Lycopene beta/epsilon cyclase protein | CHLREDRAFT_187094 | LCYE | estExt_fgenesh2_pg.C_30179 | 6.36E-05 | -3.76E+00 |
| Locus_29694_9Transcript_1/1_Confidence_1.000_Length_615 | Cre06.g267200.t1.1 | NA | CHLREDRAFT_182302 | NUO21 | estExt_fgenesh2_kg.C_30055 | 1.02E-02 | -2.00E+00 |
| Locus_2011_4Transcript_1/1_Confidence_1.000_Length_729 | Cre06.g265800.t1.1 | Ribosomal L28 family | CHLREDRAFT_196284 | PRPL28 | MAY_chlre3.3.13.1.1 | 9.51E-04 | -2.45E+00 |
| Locus_26509_9Transcript_1/1_Confidence_1.000_Length_727 | Cre06.g264350.t1.1 | Ribosomal protein L13 family protein | CHLREDRAFT_195619 | PRPL13 | MAY_chlre3.3.24.1.11 | 2.20E-06 | -2.51E+00 |
| Locus_3473_8Transcript_1/1_Confidence_1.000_Length_1139 | Cre06.g264200.t1.1 | succinate dehydrogenase 2-2 | CHLREDRAFT_142231 | SDH2 | Chlre2_kg.scaffold_3000204 | 1.20E-03 | 1.21E+01 |
| Locus_1361_3Transcript_5/5_Confidence_0.714_Length_2064 | Cre06.g264050.t1.1 | RING/FYVE/PHD-type zinc finger family protein | CHLREDRAFT_378310 | ? | estExt_fgenesh1_pm.C_chromosome_60167 | 1.86E-07 | 4.18E+00 |
| Locus_8497_5Transcript_1/1_Confidence_1.000_Length_1992 | Cre06.g262100.t1.2 | glutathione-disulfide reductase | CHLREDRAFT_134165 | GSHR1 | estExt_gwp_1W.C_30055 | 3.17E-02 | -2.09E+00 |
| Locus_3298_7Transcript_6/6_Confidence_0.682_Length_1500 | Cre06.g261750.t1.1 | Bestrophin-like protein | CHLREDRAFT_401879 | ? | pasa_Sanger_mRNA26287 | 4.51E-02 | -2.18E+00 |
| Locus_14215_9Transcript_1/1_Confidence_1.000_Length_1026 | Cre06.g260200.t1.1 | Mitochondrial substrate carrier family protein | CHLREDRAFT_155742 | MITC18 | acegs_kg.scaffold_3000117 | 6.40E-03 | 2.66E+00 |
| Locus_12386_9Transcript_1/1_Confidence_1.000_Length_1366 | Cre06.g259900.t1.1 | ATPase, F1 complex, gamma subunit protein | CHLREDRAFT_134235 | ATPC | estExt_gwp_1W.C_30183 | 2.34E-06 | -2.35E+00 |
| Locus_25403_7Transcript_1/1_Confidence_1.000_Length_394 | Cre06.g259400.t1.1 | NA | CHLREDRAFT_294849 | ? | au.g12585_t1 | 7.72E-13 | -1.08E+01 |
| Locus_6611_1Transcript_1/1_Confidence_1.000_Length_4720 | Cre06.g258700.t1.2 | methylcrotonyl-CoA carboxylase alpha chain, mitochondrial / 3-methylcrotonyl-CoA carboxylase 1 (MCCA) | CHLREDRAFT_402089 | ? | pasa_Sanger_mRNA27640 | 3.32E-02 | 2.99E+00 |
| Locus_5756_7Transcript_1/1_Confidence_1.000_Length_1012 | Cre06.g257601.t1.1 | 2-cysteine peroxiredoxin B | CHLREDRAFT_142363 | PRX1 | Chlre2_kg.scaffold_3000336 | 1.72E-02 | -5.20E+00 |
| Locus_8085_1Transcript_1/1_Confidence_1.000_Length_2303 | Cre06.g254400.t1.2 | NA | CHLREDRAFT_195953 | FUM1 | FER_fgenesh2_pg.C_scaffold_3000448 | 2.33E-03 | 4.75E+00 |
| Locus_3186_9Transcript_1/1_Confidence_1.000_Length_868 | Cre06.g253350.t1.1 | Single hybrid motif superfamily protein | CHLREDRAFT_196067 | GCSH | FER_chlre3.3.26.2.91 | 8.74E-05 | -2.14E+00 |
| Locus_5080_3Transcript_2/2_Confidence_0.750_Length_1106 | Cre06.g252000.t1.1 | NA | CHLREDRAFT_182390 | CHLREDRAFT_182390 | estExt_fgenesh2_kg.C_30157 | 6.77E-03 | -3.59E+00 |
| Locus_3511_8Transcript_1/1_Confidence_1.000_Length_1237 | Cre06.g251900.t1.1 | 63 kDa inner membrane family protein | CHLREDRAFT_187295 | ALB3.1 | estExt_fgenesh2_pg.C_30493 | 1.29E-03 | -3.48E+00 |
| Locus_4814_8Transcript_1/1_Confidence_1.000_Length_1125 | Cre06.g251600.t1.1 | Translation initiation factor IF2/IF5 | CHLREDRAFT_206391 | ? | FER_Chlre2_kg.scaffold_3000438 | 3.94E-02 | -2.23E+00 |
| Locus_75_6Transcript_4/8_Confidence_0.706_Length_5045 | Cre06.g250912.t1.1 | Homocysteine S-methyltransferase family protein | CHLREDRAFT_76715 | METH1 | estExt_GenewiseW_1.C_30026 | 6.44E-03 | -2.12E+00 |
| Locus_2724_8Transcript_1/1_Confidence_1.000_Length_1781 | Cre06.g250200.t1.1 | methionine adenosyltransferase 3 | CHLREDRAFT_182408 | METM | estExt_fgenesh2_kg.C_30176 | 8.19E-19 | -4.88E+00 |
| Locus_15712_3Transcript_4/4_Confidence_0.750_Length_2506 | Cre05.g248300.t1.2 | natural resistance associated macrophage protein 4 | CHLREDRAFT_410734 | ? | pasa_Sanger_mRNA25591 | 2.57E-03 | -7.81E+00 |
| Locus_429_2Transcript_2/2_Confidence_0.667_Length_582 | Cre05.g247600.t1.1 | ubiquitin E2 variant 1D-4 | CHLREDRAFT_187409 | UBC2 | estExt_fgenesh2_pg.C_60116 | 4.13E-03 | -2.13E+00 |
| Locus_5078_8Transcript_1/1_Confidence_1.000_Length_1254 | Cre05.g247450.t1.1 | Rhodanese/Cell cycle control phosphatase superfamily protein | CHLREDRAFT_167673 | CGL56 | fgenesh2_pg.C_scaffold_6000118 | 2.48E-02 | -3.74E+00 |
| Locus_2848_4Transcript_1/1_Confidence_1.000_Length_796 | Cre05.g246800.t1.1 | enzyme binding;tetrapyrrole binding | CHLREDRAFT_205768 | GUN4 | SKA_estExt_fgenesh2_pg.C_60106 | 3.49E-10 | -6.98E+00 |
| Locus_10226_7Transcript_1/1_Confidence_1.000_Length_838 | Cre05.g244950.t1.1 | NA | CHLREDRAFT_182433 | CHLREDRAFT_182433 | estExt_fgenesh2_kg.C_60022 | 2.87E-02 | -2.63E+00 |
| Locus_103_7Transcript_2/2_Confidence_0.750_Length_948 | Cre05.g243800.t1.1 | photosystem II family protein | CHLREDRAFT_187371 | CPLD45 | estExt_fgenesh2_pg.C_60053 | 2.39E-16 | -8.31E+00 |
| Locus_3780_6Transcript_1/1_Confidence_1.000_Length_853 | Cre05.g243050.t1.1 | thioredoxin F-type 1 | CHLREDRAFT_139781 | TRXf1 | estExt_gwp_1W.C_510048 | 2.06E-07 | -8.95E+00 |
| Locus_4958_7Transcript_1/1_Confidence_1.000_Length_991 | Cre05.g242400.t1.1 | proton gradient regulation 5 | CHLREDRAFT_127079 | CHLREDRAFT_127079 | estExt_gwp_1H.C_60062 | 2.10E-08 | -5.07E+00 |
| Locus_1706_2Transcript_1/1_Confidence_1.000_Length_3005 | Cre05.g242000.t1.1 | ALBINA 1 | CHLREDRAFT_134594 | CHLD | estExt_gwp_1W.C_60238 | 4.51E-08 | -3.85E+00 |
| Locus_5806_6Transcript_1/1_Confidence_1.000_Length_1215 | Cre05.g240150.t1.1 | peroxin4 | CHLREDRAFT_378125 | ? | estExt_fgenesh1_pm.C_chromosome_50068 | 9.36E-05 | -4.15E+00 |
| Locus_6708_8Transcript_1/1_Confidence_1.000_Length_1565 | Cre05.g237050.t1.2 | Protein of unknown function (DUF1230) | CHLREDRAFT_184984 | CGLD27 | estExt_fgenesh2_kg.C_360028 | 3.37E-02 | -2.72E+00 |
| Locus_249_4Transcript_1/1_Confidence_1.000_Length_783 | Cre05.g233950.t1.1 | NA | CHLREDRAFT_305381 | ? | kg.chromosome_5_#_64_#_TC47957 | 7.08E-10 | -2.62E+00 |
| Locus_12590_10Transcript_1/2_Confidence_0.667_Length_1153 | Cre05.g233900.t1.1 | ascorbate peroxidase 4 | CHLREDRAFT_206548 | ? | SKA_fgenesh2_kg.C_scaffold_74000005 | 5.38E-19 | -9.04E+00 |
| Locus_6145_7Transcript_1/1_Confidence_1.000_Length_1445 | Cre05.g233150.t1.2 | NAD(P)-binding Rossmann-fold superfamily protein | CHLREDRAFT_294234 | ? | au.g12006_t2 | 1.01E-07 | -4.81E+00 |
| Locus_511_4Transcript_1/1_Confidence_1.000_Length_2034 | Cre04.g229300.t1.2 | rubisco activase | CHLREDRAFT_128745 | RCA1 | estExt_gwp_1H.C_150086 | 2.52E-07 | -5.55E+00 |
| Locus_7752_2Transcript_3/3_Confidence_0.750_Length_1349 | Cre04.g227400.t1.1 | ferric reduction oxidase 2 | CHLREDRAFT_205609 | FRE1 | SKA_Chlre2_kg.scaffold_15000162 | 1.84E-09 | -1.12E+01 |
| Locus_424_9Transcript_1/1_Confidence_1.000_Length_1027 | Cre04.g225650.t1.1 | Cystatin/monellin superfamily protein | CHLREDRAFT_183419 | CHLREDRAFT_183419 | estExt_fgenesh2_kg.C_150044 | 3.47E-09 | 3.33E+00 |
| Locus_3206_1Transcript_2/2_Confidence_0.667_Length_1352 | Cre04.g216200.t1.1 | NA | CHLREDRAFT_415443 | ? | pasa_Sanger_mRNA18963 | 1.40E-02 | 3.52E+00 |
| Locus_1314_4Transcript_1/1_Confidence_1.000_Length_1332 | Cre04.g214150.t1.2 | thiazole biosynthetic enzyme, chloroplast (ARA6) (THI1) (THI4) | CHLREDRAFT_185190 | ? | estExt_fgenesh2_kg.C_400018 | 3.32E-03 | -5.25E+00 |
| Locus_25821_2Transcript_1/1_Confidence_1.000_Length_889 | Cre04.g212500.t1.1 | Domain of unknown function (DUF1995) | CHLREDRAFT_205904 | CGL42 | SKA_Chlre2_kg.scaffold_40000074 | 4.60E-02 | -1.44E+01 |
| Locus_20669_9Transcript_1/1_Confidence_1.000_Length_1234 | Cre03.g208050.t1.1 | Thioesterase superfamily protein | CHLREDRAFT_293493 | ? | au.g11300_t1 | 1.07E-05 | -2.60E+00 |
| Locus_4275_2Transcript_1/1_Confidence_1.000_Length_1212 | Cre03.g207300.t1.2 | NA | CHLREDRAFT_147467 | CHLREDRAFT_147467 | Chlre2_kg.scaffold_19000141 | 4.49E-02 | 2.44E+00 |
| Locus_8529_3Transcript_1/1_Confidence_1.000_Length_1224 | Cre03.g207000.t1.1 | abscisic acid (aba)-deficient 4 | CHLREDRAFT_377933 | ? | estExt_fgenesh1_pm.C_chromosome_30615 | 1.81E-03 | -1.93E+01 |
| Locus_60_2Transcript_1/1_Confidence_1.000_Length_2787 | Cre03.g206600.t1.1 | dehydratase family | CHLREDRAFT_129639 | AAD1 | estExt_gwp_1H.C_190078 | 9.78E-05 | 5.90E+00 |
| Locus_2695_3Transcript_2/2_Confidence_0.750_Length_2093 | Cre03.g205900.t1.1 | S-adenosylmethionine decarboxylase | CHLREDRAFT_183928 | DCA1 | estExt_fgenesh2_kg.C_190068 | 7.14E-04 | -3.63E+00 |
| Locus_5931_7Transcript_1/1_Confidence_1.000_Length_1264 | Cre03.g204550.t1.2 | adenosine kinase 1 | CHLREDRAFT_190547 | FAP278 | estExt_fgenesh2_pg.C_190195 | 7.85E-04 | -2.65E+00 |
| Locus_2776_1Transcript_1/1_Confidence_1.000_Length_2259 | Cre03.g204250.t1.1 | S-adenosyl-L-homocysteine hydrolase | CHLREDRAFT_129593 | SAH1 | estExt_gwp_1H.C_190014 | 5.74E-04 | -3.53E+00 |
| Locus_1166_5Transcript_1/1_Confidence_1.000_Length_1870 | Cre03.g203850.t1.1 | Pseudouridine synthase/archaeosine transglycosylase-like family protein | CHLREDRAFT_196910 | ATS1 | HAL_estExt_fgenesh2_kg.C_190083 | 1.08E-03 | -2.15E+00 |
| Locus_4500_2Transcript_1/3_Confidence_0.778_Length_3788 | Cre03.g199050.t1.1 | Protein kinase family protein | CHLREDRAFT_377876 | ? | estExt_fgenesh1_pm.C_chromosome_30541 | 3.81E-02 | -3.80E+01 |
| Locus_2825_8Transcript_1/1_Confidence_1.000_Length_1266 | Cre03.g198950.t1.1 | Mog1/PsbP/DUF1795-like photosystem II reaction center PsbP family protein | CHLREDRAFT_205900 | CGL30 | SKA_acegs_kg.scaffold_19000130 | 2.66E-03 | -3.84E+00 |
| Locus_1099_3Transcript_3/3_Confidence_0.667_Length_1053 | Cre03.g198850.t1.1 | Mog1/PsbP/DUF1795-like photosystem II reaction center PsbP family protein | CHLREDRAFT_205649 | CHLREDRAFT_205649 | OVA_estExt_fgenesh2_kg.C_190112 | 5.65E-05 | -6.47E+00 |
| Locus_2624_4Transcript_1/1_Confidence_1.000_Length_662 | Cre03.g197750.t1.1 | glutathione peroxidase 1 | CHLREDRAFT_137012 | GPX3 | estExt_gwp_1W.C_190017 | 4.56E-08 | -2.23E+00 |
| Locus_3283_8Transcript_1/1_Confidence_1.000_Length_1062 | Cre03.g195650.t1.1 | Ribosomal protein S10p/S20e family protein | CHLREDRAFT_195637 | PRPS10 | MAY_e_gwH.19.249.1 | 1.29E-02 | -2.14E+00 |
| Locus_236_3Transcript_3/9_Confidence_0.281_Length_1249 | Cre03.g194350.t1.1 | proton pump interactor 1 | CHLREDRAFT_377831 | ? | estExt_fgenesh1_pm.C_chromosome_30478 | 5.84E-04 | -3.28E+00 |
| Locus_2422_7Transcript_1/1_Confidence_1.000_Length_1518 | Cre03.g194200.t1.1 | pyruvate dehydrogenase E1 beta | CHLREDRAFT_190446 | PDH2 | estExt_fgenesh2_pg.C_190047 | 3.21E-05 | -3.72E+00 |
| Locus_5428_1Transcript_1/1_Confidence_1.000_Length_1254 | Cre03.g193850.t1.1 | Succinyl-CoA ligase, alpha subunit | CHLREDRAFT_196570 | SCLA1 | MHS_estExt_GenewiseH_1.C_190100 | 2.27E-07 | 4.03E+00 |
| Locus_1969_2Transcript_1/1_Confidence_1.000_Length_1819 | Cre03.g193750.t1.2 | Glycine cleavage T-protein family | CHLREDRAFT_196242 | GCST | BTM_chlre3.19.251.1.1 | 3.83E-05 | -4.96E+00 |
| Locus_2325_3Transcript_3/4_Confidence_0.385_Length_1297 | Cre03.g193300.t1.1 | Haloacid dehalogenase-like hydrolase (HAD) superfamily protein | CHLREDRAFT_418706 | ? | pasa_Sanger_mRNA23933 | 2.67E-04 | 2.62E+00 |
| Locus_10898_5Transcript_1/1_Confidence_1.000_Length_1582 | Cre03.g189800.t1.1 | cyclophilin 38 | CHLREDRAFT_196558 | CYN38 | OVA_estExt_GenewiseH_1.C_140074 | 1.33E-09 | -1.12E+01 |
| Locus_6536_8Transcript_1/1_Confidence_1.000_Length_1088 | Cre03.g187450.t1.1 | Ribose 5-phosphate isomerase, type A protein | CHLREDRAFT_55838 | RPI1 | estExt_GenewiseH_1.C_140147 | 4.39E-02 | -3.08E+00 |
| Locus_61_4Transcript_1/1_Confidence_1.000_Length_841 | Cre03.g183850.t1.1 | 2Fe-2S ferredoxin-like superfamily protein | CHLREDRAFT_196703 | FDX6 | OVA_AIM_query | 2.52E-08 | -3.92E+00 |
| Locus_524_4Transcript_1/1_Confidence_1.000_Length_740 | Cre03.g182551.t1.1 | plastocyanin 1 | CHLREDRAFT_185915 | PCY1 | estExt_fgenesh2_kg.C_820018 | 8.79E-04 | -1.22E+01 |
| Locus_2868_9Transcript_1/1_Confidence_1.000_Length_1276 | Cre03.g182150.t1.1 | thylakoid lumen 18.3 kDa protein | CHLREDRAFT_60278 | TEF8 | estExt_GenewiseH_1.C_820026 | 4.62E-04 | -2.79E+00 |
| Locus_2824_2Transcript_1/1_Confidence_1.000_Length_2405 | Cre03.g181500.t1.1 | disproportionating enzyme | CHLREDRAFT_140452 | STA11 | estExt_gwp_1W.C_820038 | 2.57E-03 | 2.29E+00 |
| Locus_1953_3Transcript_1/1_Confidence_1.000_Length_2651 | Cre03.g181300.t1.1 | RNA 3\'-terminal phosphate cyclase/enolpyruvate transferase, alpha/beta | CHLREDRAFT_133088 | SHKG1 | estExt_gwp_1H.C_820057 | 1.69E-06 | -8.56E+00 |
| Locus_9294_7Transcript_1/1_Confidence_1.000_Length_1119 | Cre03.g181250.t1.1 | NAD(P)-binding Rossmann-fold superfamily protein | CHLREDRAFT_205577 | CGLD13 | SKA_chlre3.82.52.2.51 | 1.50E-04 | -5.06E+00 |
| Locus_599_2Transcript_1/1_Confidence_1.000_Length_2854 | Cre03.g180750.t1.1 | methionine synthase 3 | CHLREDRAFT_154307 | METE | Chlre2_kg.scaffold_82000007 | 3.72E-02 | -1.99E+01 |
| Locus_266_4Transcript_1/1_Confidence_1.000_Length_1270 | Cre03.g174850.t1.1 | Polyketide cyclase/dehydrase and lipid transport superfamily protein | CHLREDRAFT_188173 | CHLREDRAFT_188173 | estExt_fgenesh2_pg.C_90102 | 4.34E-09 | -6.16E+00 |
| Locus_14014_2Transcript_1/1_Confidence_1.000_Length_1452 | Cre03.g172550.t1.1 | arginine methyltransferase 11 | CHLREDRAFT_205758 | PRMT1 | SKA_fgenesh2_pg.C_scaffold_9000144 | 4.83E-02 | 2.55E+00 |
| Locus_5215_8Transcript_1/1_Confidence_1.000_Length_1966 | Cre03.g172500.t1.2 | Alternative oxidase family protein | CHLREDRAFT_205757 | PTOX2 | SKA_estExt_fgenesh2_pg.C_90141 | 2.47E-04 | 1.57E+01 |
| Locus_4386_10Transcript_3/4_Confidence_0.667_Length_1378 | Cre03.g172300.t1.1 | phosphate transporter 3;1 | CHLREDRAFT_135199 | ? | estExt_gwp_1W.C_90220 | 1.59E-07 | 2.37E+00 |
| Locus_1558_4Transcript_1/1_Confidence_1.000_Length_930 | Cre03.g172000.t1.1 | NAD(P)-binding Rossmann-fold superfamily protein | CHLREDRAFT_335991 | ? | pm.chromosome_3_#_252 | 9.59E-10 | -3.51E+00 |
| Locus_3277_10Transcript_1/1_Confidence_1.000_Length_662 | Cre03.g165100.t1.1 | photsystem I subunit I | CHLREDRAFT_144056 | PSAI | Chlre2_kg.scaffold_9000287 | 5.60E-03 | -3.00E+00 |
| Locus_6355_6Transcript_1/1_Confidence_1.000_Length_1525 | Cre03.g164000.t1.1 | NA | CHLREDRAFT_188287 | TEF7 | estExt_fgenesh2_pg.C_90288 | 2.36E-02 | -3.21E+00 |
| Locus_1990_7Transcript_1/2_Confidence_0.667_Length_1406 | Cre03.g160750.t1.2 | Cation efflux family protein | CHLREDRAFT_150292 | MTP2 | Chlre2_kg.scaffold_34000008 | 2.16E-02 | -4.15E+00 |
| Locus_2047_8Transcript_5/10_Confidence_0.333_Length_2016 | Cre03.g159800.t1.2 | RELA/SPOT homolog 3 | CHLREDRAFT_419232 | ? | pasa_Sanger_mRNA23263.1 | 9.69E-05 | 2.50E+00 |
| Locus_13812_7Transcript_1/1_Confidence_1.000_Length_1118 | Cre03.g158900.t1.1 | 2-oxoacid dehydrogenases acyltransferase family protein | CHLREDRAFT_196500 | DLA2 | MHS_53765321 | 6.78E-06 | -4.83E+00 |
| Locus_395_5Transcript_1/1_Confidence_1.000_Length_1948 | Cre03.g158000.t1.1 | glutamate-1-semialdehyde 2,1-aminomutase 2 | CHLREDRAFT_138524 | GSA | estExt_gwp_1W.C_340068 | 4.58E-05 | -3.77E+00 |
| Locus_15521_1Transcript_1/1_Confidence_1.000_Length_1340 | Cre03.g156050.t1.1 | ribosome recycling factor, chloroplast precursor | CHLREDRAFT_303299 | ? | kg.chromosome_3_#_229_#_TC48423 | 9.32E-03 | -5.83E+00 |
| Locus_61884_10Transcript_1/1_Confidence_1.000_Length_396 | Cre03.g151200.t1.1 | NA | CHLREDRAFT_163712 | CGLD16 | fgenesh2_kg.C_scaffold_27000007 | 9.44E-03 | -6.68E+00 |
| Locus_8472_3Transcript_6/6_Confidence_0.450_Length_1956 | Cre02.g145800.t1.1 | Lactate/malate dehydrogenase family protein | CHLREDRAFT_158129 | MDH3 | acegs_kg.scaffold_24000043 | 3.89E-02 | 5.27E+00 |
| Locus_201_6Transcript_27/27_Confidence_0.133_Length_3130 | Cre02.g144700.t1.1 | phosphate transporter 2;1 | CHLREDRAFT_196438 | PTB5 | JLM_estExt_GenewiseW_1.C_240191 | 1.09E-03 | -3.68E+00 |
| Locus_7226_2Transcript_1/1_Confidence_1.000_Length_1410 | Cre02.g143900.t1.2 | GDSL-like Lipase/Acylhydrolase superfamily protein | CHLREDRAFT_394987 | ? | pasa_Sanger_mRNA12745 | 9.71E-05 | -5.48E+00 |
| Locus_806_5Transcript_1/1_Confidence_1.000_Length_1661 | Cre02.g143250.t1.1 | isocitrate dehydrogenase VI | CHLREDRAFT_196044 | IDH2 | OVA_estExt_gwp_1W.C_240106 | 3.75E-03 | 2.16E+00 |
| Locus_7671_8Transcript_2/4_Confidence_0.357_Length_1578 | Cre02.g143000.t1.1 | phospholipid/glycerol acyltransferase family protein | CHLREDRAFT_205741 | PLSB1 | SKA_estExt_gwp_1H.C_240205 | 2.81E-02 | -2.39E+00 |
| Locus_788_7Transcript_1/5_Confidence_0.143_Length_1588 | Cre02.g142300.t1.2 | molybdenum cofactor sulfurase (LOS5) (ABA3) | CHLREDRAFT_343193 | ? | estExt_fgenesh1_pg.C_chromosome_20949 | 1.79E-02 | 2.41E+00 |
| Locus_27829_2Transcript_1/1_Confidence_1.000_Length_1424 | Cre02.g140300.t1.2 | Copper amine oxidase family protein | CHLREDRAFT_176519 | AMX1 | fgenesh2_pg.C_scaffold_35000049 | 3.83E-08 | 1.34E+01 |
| Locus_6898_10Transcript_1/3_Confidence_0.333_Length_1234 | Cre02.g132850.t1.1 | glutathione reductase | CHLREDRAFT_191427 | GSHR2 | estExt_fgenesh2_pg.C_230111 | 1.66E-04 | -4.15E+00 |
| Locus_727_4Transcript_1/1_Confidence_1.000_Length_2807 | Cre02.g130650.t1.1 | Inorganic H pyrophosphatase family protein | CHLREDRAFT_137778 | CHLREDRAFT_137778 | estExt_gwp_1W.C_230107 | 1.60E-05 | -4.43E+00 |
| Locus_6637_6Transcript_1/1_Confidence_1.000_Length_1017 | Cre02.g129000.t1.1 | Chlorophyll A-B binding family protein | CHLREDRAFT_137766 | ELI2-1 | estExt_gwp_1W.C_230089 | 5.68E-05 | -8.02E+00 |
| Locus_6667_10Transcript_7/7_Confidence_0.500_Length_750 | Cre02.g127852.t1.1 | NA | CHLREDRAFT_417251 | ? | pasa_Sanger_mRNA21079 | 6.91E-03 | 4.49E+00 |
| Locus_1923_2Transcript_1/1_Confidence_1.000_Length_521 | Cre02.g118950.t1.1 | Nucleic acid-binding, OB-fold-like protein | CHLREDRAFT_195639 | PRPS17 | MAY_chlre3.79.1.2.11 | 8.13E-04 | -2.40E+00 |
| Locus_29886_9Transcript_2/2_Confidence_0.667_Length_477 | Cre02.g118850.t1.1 | acyl-CoA-binding protein 6 | CHLREDRAFT_133008 | CHLREDRAFT_133008 | estExt_gwp_1H.C_790003 | 1.62E-03 | -2.37E+00 |
| Locus_44325_9Transcript_1/1_Confidence_1.000_Length_798 | Cre02.g118550.t1.1 | NA | CHLREDRAFT_416138 | ? | pasa_Sanger_mRNA20877 | 1.24E-02 | -7.94E+00 |
| Locus_96_4Transcript_1/1_Confidence_1.000_Length_1881 | Cre02.g113200.t1.2 | glutamine synthetase 1.3 | CHLREDRAFT_133971 | GLN1 | estExt_gwp_1W.C_50109 | 3.45E-28 | 2.62E+00 |
| Locus_22688_5Transcript_1/1_Confidence_1.000_Length_949 | Cre02.g111450.t1.1 | Rhodanese/Cell cycle control phosphatase superfamily protein | CHLREDRAFT_182149 | TEF4 | estExt_fgenesh2_kg.C_50051 | 6.31E-15 | -4.70E+00 |
| Locus_4378_7Transcript_1/2_Confidence_0.667_Length_561 | Cre02.g110300.t1.1 | Chaperone DnaJ-domain superfamily protein | CHLREDRAFT_29309 | DNJ25 | estExt_fgenesh1_pg.C_50164 | 9.59E-03 | -4.68E+00 |
| Locus_1717_4Transcript_1/1_Confidence_1.000_Length_286 | Cre02.g109950.t1.1 | one helix protein | CHLREDRAFT_133963 | HLIP | estExt_gwp_1W.C_50098 | 1.59E-02 | -3.48E+00 |
| Locus_5136_7Transcript_1/1_Confidence_1.000_Length_960 | Cre02.g108850.t1.1 | Ribosomal protein L17 family protein | CHLREDRAFT_195620 | PRPL17 | MAY_chlre3.5.118.2.11 | 4.21E-04 | -2.51E+00 |
| Locus_3136_4Transcript_1/1_Confidence_1.000_Length_1097 | Cre02.g105200.t1.1 | saposin B domain-containing protein | CHLREDRAFT_301657 | ? | kg.chromosome_2_#_1096_#_chlre3.5.11.1.1 | 2.64E-11 | 2.57E+00 |
| Locus_19686_7Transcript_1/1_Confidence_1.000_Length_762 | Cre02.g102000.t1.1 | ADP-ribosylation factor C1 | CHLREDRAFT_195528 | ARL5 | SAN_e_gwW.5.137.1 | 3.10E-02 | -4.92E+00 |
| Locus_1463_5Transcript_1/1_Confidence_1.000_Length_1562 | Cre02.g097900.t1.1 | aspartate aminotransferase 5 | CHLREDRAFT_186959 | AST3 | estExt_fgenesh2_pg.C_50411 | 4.38E-08 | -2.16E+00 |
| Locus_673_3Transcript_2/6_Confidence_0.538_Length_798 | Cre02.g096150.t1.1 | manganese superoxide dismutase 1 | CHLREDRAFT_53941 | MSD1 | estExt_GenewiseH_1.C_50316 | 1.25E-08 | -3.47E+00 |
| Locus_781_4Transcript_1/1_Confidence_1.000_Length_719 | Cre02.g093650.t1.1 | Rieske (2Fe-2S) domain-containing protein | CHLREDRAFT_182093 | CHLREDRAFT_182093 | estExt_fgenesh2_kg.C_70171 | 7.60E-04 | 3.83E+00 |
| Locus_8737_8Transcript_1/1_Confidence_1.000_Length_1237 | Cre02.g093450.t1.1 | Aldolase superfamily protein | CHLREDRAFT_29185 | FBA2 | estExt_fgenesh1_pg.C_70454 | 5.83E-04 | -4.00E+00 |
| Locus_349_8Transcript_2/2_Confidence_0.889_Length_2028 | Cre02.g092350.t1.1 | CYTOCHROME P450 51G1 | CHLREDRAFT_196411 | CYP51G1 | BTM_estExt_gwp_1H.C_70049 | 1.85E-07 | -3.64E+00 |
| Locus_495_6Transcript_1/1_Confidence_1.000_Length_1750 | Cre02.g091050.t1.1 | Aldolase superfamily protein | CHLREDRAFT_186639 | ALAD | estExt_fgenesh2_pg.C_70362 | 7.32E-17 | -7.16E+00 |
| Locus_7571_9Transcript_1/1_Confidence_1.000_Length_994 | Cre02.g088900.t1.1 | Ribosomal protein L1p/L10e family | CHLREDRAFT_126226 | PRPL1 | estExt_gwp_1H.C_70008 | 1.08E-02 | -2.07E+00 |
| Locus_2721_5Transcript_1/1_Confidence_1.000_Length_853 | Cre02.g088850.t1.1 | 6,7-dimethyl-8-ribityllumazine synthase / DMRL synthase / lumazine synthase / riboflavin synthase | CHLREDRAFT_186616 | CHLREDRAFT_186616 | estExt_fgenesh2_pg.C_70324 | 9.22E-10 | -6.46E+00 |
| Locus_169_2Transcript_3/5_Confidence_0.632_Length_1062 | Cre02.g087700.t1.1 | stromal ascorbate peroxidase | CHLREDRAFT_186597 | APX1 | estExt_fgenesh2_pg.C_70298 | 9.20E-03 | 4.22E+00 |
| Locus_3345_10Transcript_5/6_Confidence_0.421_Length_1011 | Cre02.g087250.t1.1 | NA | CHLREDRAFT_342717 | ? | estExt_fgenesh1_pg.C_chromosome_20200 | 4.29E-09 | -5.24E+00 |
| Locus_4791_7Transcript_1/1_Confidence_1.000_Length_1716 | Cre02.g085900.t1.1 | myo-inositol monophosphatase like 1 | CHLREDRAFT_376952 | ? | estExt_fgenesh1_pm.C_chromosome_20164 | 2.14E-05 | -4.54E+00 |
| Locus_1692_5Transcript_1/1_Confidence_1.000_Length_1821 | Cre02.g085450.t1.1 | Coproporphyrinogen III oxidase | CHLREDRAFT_53583 | CPX1 | estExt_GenewiseH_1.C_70119 | 1.77E-12 | -8.74E+00 |
| Locus_3191_6Transcript_2/2_Confidence_0.667_Length_502 | Cre02.g084000.t1.1 | NA | CHLREDRAFT_182023 | CHLREDRAFT_182023 | estExt_fgenesh2_kg.C_70093 | 4.39E-02 | -2.70E+00 |
| Locus_1080_5Transcript_1/2_Confidence_0.667_Length_1039 | Cre02.g083950.t1.1 | Ribosomal protein PSRP-3/Ycf65 | CHLREDRAFT_195633 | PSRP-3 | MAY_chlre3.7.7.2.11 | 1.88E-05 | -2.70E+00 |
| Locus_70574_10Transcript_1/2_Confidence_0.667_Length_351 | Cre02.g079200.t1.1 | nuclear factor Y, subunit B3 | CHLREDRAFT_111791 | CHLREDRAFT_111791 | e_gwW.7.372.1 | 1.23E-02 | 4.12E+00 |
| Locus_22105_7Transcript_1/1_Confidence_1.000_Length_696 | Cre02.g077500.t1.1 | SNARE-like superfamily protein | CHLREDRAFT_53681 | TRS23 | estExt_GenewiseH_1.C_70268 | 2.47E-02 | 5.86E+00 |
| Locus_8255_3Transcript_1/1_Confidence_1.000_Length_1645 | Cre02.g073700.t1.1 | Uroporphyrinogen decarboxylase | CHLREDRAFT_195818 | UROD3 | RWI_chlre3.7.129.1.1 | 1.30E-03 | -6.08E+00 |
| Locus_7041_6Transcript_1/3_Confidence_0.400_Length_442 | Cre01.g068150.t1.1 | secE/sec61-gamma protein transport protein | CHLREDRAFT_185246 | SEC61G | estExt_fgenesh2_kg.C_420030 | 1.54E-04 | -4.77E+00 |
| Locus_11366_9Transcript_1/3_Confidence_0.143_Length_1095 | Cre01.g068100.t1.1 | receptor homology region transmembrane domain ring H2 motif protein 1 | CHLREDRAFT_407573 | ? | pasa_Sanger_mRNA1516 | 1.07E-02 | 2.20E+00 |
| Locus_5641_1Transcript_1/1_Confidence_1.000_Length_1608 | Cre01.g067900.t1.1 | Nucleotide-diphospho-sugar transferases superfamily protein | CHLREDRAFT_196604 | CMS | LOH_163105 | 2.76E-04 | -5.73E+00 |
| Locus_8646_8Transcript_2/2_Confidence_0.667_Length_724 | Cre01.g066750.t1.2 | CCT motif -containing response regulator protein | CHLREDRAFT_404672 | ? | pasa_Sanger_mRNA3156 | 1.75E-02 | 1.39E+01 |
| Locus_1906_9Transcript_4/4_Confidence_0.750_Length_599 | Cre01.g063100.t1.1 | Protein phosphatase 2C family protein | CHLREDRAFT_164613 | PP2C2 | fgenesh2_kg.C_scaffold_45000038 | 3.25E-05 | 7.23E+00 |
| Locus_12546_6Transcript_1/1_Confidence_1.000_Length_1071 | Cre01.g060900.t1.1 | NA | CHLREDRAFT_149843 | ? | Chlre2_kg.scaffold_27000112 | 3.27E-03 | -4.34E+00 |
| Locus_411_6Transcript_1/1_Confidence_1.000_Length_2011 | Cre01.g054150.t1.1 | NADPH-dependent thioredoxin reductase C | CHLREDRAFT_78928 | NTRC1 | estExt_GenewiseW_1.C_10098 | 1.85E-05 | -7.13E+00 |
| Locus_37235_10Transcript_1/1_Confidence_1.000_Length_534 | Cre01.g052100.t1.1 | Ribosomal L18p/L5e family protein | CHLREDRAFT_196265 | PRPL18 | MAY_Chlre2_kg.scaffold_1000753 | 4.90E-02 | -2.12E+00 |
| Locus_2688_8Transcript_1/1_Confidence_1.000_Length_2163 | Cre01.g050950.t1.1 | Pyridine nucleotide-disulphide oxidoreductase family protein | CHLREDRAFT_136810 | CHLREDRAFT_136810 | estExt_gwp_1W.C_10724 | 2.27E-03 | -1.74E+01 |
| Locus_55645_10Transcript_1/1_Confidence_1.000_Length_409 | Cre01.g050550.t1.1 | 2 iron, 2 sulfur cluster binding | CHLREDRAFT_407414 | ? | pasa_Sanger_mRNA2810 | 1.46E-06 | 9.63E+00 |
| Locus_15615_7Transcript_1/1_Confidence_1.000_Length_802 | Cre01.g046700.t1.1 | Nucleic acid-binding, OB-fold-like protein | CHLREDRAFT_376627 | ? | estExt_fgenesh1_pm.C_chromosome_10551 | 8.99E-03 | -5.66E+00 |
| Locus_1527_4Transcript_1/1_Confidence_1.000_Length_1926 | Cre01.g045550.t1.1 | Glucose-6-phosphate/phosphate translocator-related | CHLREDRAFT_205633 | APE2 | SKA_Chlre2_kg.scaffold_1000642 | 1.51E-07 | 2.14E+00 |
| Locus_3055_2Transcript_1/2_Confidence_0.667_Length_1165 | Cre01.g044100.t1.1 | beta-amylase 6 | CHLREDRAFT_129211 | AMYB3 | estExt_gwp_1H.C_10432 | 4.38E-02 | 3.85E+00 |
| Locus_3045_2Transcript_1/1_Confidence_1.000_Length_2618 | Cre01.g043350.t1.1 | Pheophorbide a oxygenase family protein with Rieske [2Fe-2S] domain | CHLREDRAFT_195951 | CAO | RWI_estExt_fgenesh2_pg.C_10626 | 2.67E-09 | -4.41E+00 |
| Locus_5483_3Transcript_6/6_Confidence_0.706_Length_1659 | Cre01.g043050.t1.1 | SNARE associated Golgi protein family | CHLREDRAFT_146768 | CHLREDRAFT_146768 | Chlre2_kg.scaffold_1000592 | 8.24E-03 | -2.85E+00 |
| Locus_5195_5Transcript_1/1_Confidence_1.000_Length_1392 | Cre01.g042800.t1.1 | NAD(P)-binding Rossmann-fold superfamily protein | CHLREDRAFT_195952 | DVR1 | RWI_154979 | 3.04E-02 | -7.32E+00 |
| Locus_139_9Transcript_1/1_Confidence_1.000_Length_3630 | Cre01.g042750.t1.1 | aconitase 2 | CHLREDRAFT_129025 | ACH1 | estExt_gwp_1H.C_10164 | 4.44E-17 | 6.16E+00 |
| Locus_9507_7Transcript_1/1_Confidence_1.000_Length_422 | Cre01.g042200.t1.1 | NA | CHLREDRAFT_190034 | CHLREDRAFT_190034 | estExt_fgenesh2_pg.C_10600 | 4.30E-09 | -9.63E+00 |
| Locus_20932_10Transcript_1/1_Confidence_1.000_Length_709 | Cre01.g040450.t1.1 | histidine-containing phosphotransfer factor 5 | CHLREDRAFT_183677 | HDPT1 | estExt_fgenesh2_kg.C_10175 | 9.05E-04 | -4.96E+00 |
| Locus_295_4Transcript_1/1_Confidence_1.000_Length_1647 | Cre01.g039300.t1.1 | poly(A) binding protein 2 | CHLREDRAFT_116226 | RB47 | e_gwW.1.40.1 | 1.80E-08 | 2.35E+00 |
| Locus_9713_5Transcript_2/2_Confidence_0.667_Length_1283 | Cre01.g037850.t1.1 | Single hybrid motif superfamily protein | CHLREDRAFT_183660 | BCC2 | estExt_fgenesh2_kg.C_10158 | 1.09E-04 | -4.61E+00 |
| Locus_27335_6Transcript_1/1_Confidence_1.000_Length_605 | Cre01.g034350.t1.2 | myb domain protein 3r-5 | CHLREDRAFT_117291 | CHLREDRAFT_117291 | e_gwW.19.195.1 | 9.07E-03 | 9.54E+00 |
| Locus_11399_5Transcript_1/1_Confidence_1.000_Length_2055 | Cre01.g033350.t1.1 | aldehyde dehydrogenase 22A1 | CHLREDRAFT_283125 | ? | au.g1636_t1 | 3.04E-04 | -5.11E+00 |
| Locus_18352_5Transcript_1/1_Confidence_1.000_Length_884 | Cre01.g033300.t1.1 | NA | CHLREDRAFT_299394 | ? | kg.chromosome_1_#_1119_#_XP_001689614.1 | 4.41E-02 | -3.70E+00 |
| Locus_9597_9Transcript_1/1_Confidence_1.000_Length_1165 | Cre01.g031100.t1.1 | protein containing PDZ domain, a K-box domain, and a TPR region | CHLREDRAFT_205993 | TEF30 | OVA_estExt_fgenesh2_kg.C_10120 | 2.08E-18 | -4.96E+00 |
| Locus_2271_1Transcript_3/4_Confidence_0.500_Length_605 | Cre01.g030050.t1.1 | Ribosomal protein L34 | CHLREDRAFT_189891 | PRPL34 | estExt_fgenesh2_pg.C_10372 | 5.59E-04 | -3.36E+00 |
| Locus_1_5Transcript_444/656_Confidence_1.000_Length_718 | Cre01.g027200.t1.1 | ubiquitin-conjugating enzyme 5 | CHLREDRAFT_183594 | CHLREDRAFT_183594 | estExt_fgenesh2_kg.C_10090 | 4.32E-02 | 2.38E+00 |
| Locus_1332_8Transcript_1/1_Confidence_1.000_Length_1892 | Cre01.g021600.t1.1 | DEAD box RNA helicase 1 | CHLREDRAFT_376403 | ? | estExt_fgenesh1_pm.C_chromosome_10239 | 3.91E-02 | 2.08E+00 |
| Locus_9818_9Transcript_1/1_Confidence_1.000_Length_505 | Cre01.g020350.t1.1 | NA | CHLREDRAFT_323482 | ? | pg.chromosome_1_#_271 | 2.91E-05 | 2.32E+01 |
| Locus_8064_3Transcript_1/1_Confidence_1.000_Length_778 | Cre01.g020300.t1.1 | NA | CHLREDRAFT_146346 | SDH4 | Chlre2_kg.scaffold_1000170 | 1.02E-04 | 4.85E+00 |
| Locus_4873_6Transcript_1/1_Confidence_1.000_Length_2210 | Cre01.g019250.t1.1 | GDP-D-mannose 3\',5\'-epimerase | CHLREDRAFT_196952 | SNE1 | HAL_estExt_GenewiseW_1.C_10190 | 2.17E-04 | -2.41E+00 |
| Locus_32474_10Transcript_1/1_Confidence_1.000_Length_557 | Cre01.g017300.t1.1 | NA | CHLREDRAFT_195631 | PRPS21 | MAY_fgenesh2_pg.C_scaffold_1000145 | 2.84E-03 | -2.49E+00 |
| Locus_2601_7Transcript_1/1_Confidence_1.000_Length_700 | Cre01.g016900.t1.1 | NA | CHLREDRAFT_183558 | CHLREDRAFT_183558 | estExt_fgenesh2_kg.C_10050 | 1.33E-03 | -2.70E+00 |
| Locus_12468_2Transcript_1/1_Confidence_1.000_Length_2605 | Cre01.g016504.t1.1 | dihydrolipoyl dehydrogenases | CHLREDRAFT_205763 | DLD2 | SKA_Chlre2_kg.scaffold_1000106 | 1.21E-02 | -6.70E+00 |
| Locus_1573_10Transcript_1/1_Confidence_1.000_Length_1503 | Cre01.g016300.t1.1 | calmodulin-like 38 | CHLREDRAFT_183554 | CHLREDRAFT_183554 | estExt_fgenesh2_kg.C_10046 | 2.64E-03 | -2.82E+00 |
| Locus_1636_4Transcript_1/1_Confidence_1.000_Length_1842 | Cre01.g015350.t1.1 | protochlorophyllide oxidoreductase A | CHLREDRAFT_136589 | POR | estExt_gwp_1W.C_10376 | 8.33E-04 | -2.58E+01 |
| Locus_902_4Transcript_1/1_Confidence_1.000_Length_1067 | Cre01.g014350.t1.1 | Thioredoxin superfamily protein | CHLREDRAFT_157173 | PRX5 | acegs_kg.scaffold_1000038 | 2.13E-03 | -3.37E+00 |
| Locus_160_2Transcript_83/239_Confidence_1.000_Length_823 | Cre01.g012900.t1.2 | ACT domain repeat 4 | CHLREDRAFT_342971 | ? | estExt_fgenesh1_pg.C_chromosome_20584 | 2.32E-02 | 4.37E+00 |
| Locus_10683_7Transcript_1/1_Confidence_1.000_Length_1116 | Cre01.g012400.t1.1 | Nodulin MtN21 /EamA-like transporter family protein | CHLREDRAFT_282687 | ? | au.g1221_t1 | 3.71E-03 | -2.82E+01 |
| Locus_7240_2Transcript_2/2_Confidence_0.750_Length_1453 | Cre01.g012400.t1.1 | Nodulin MtN21 /EamA-like transporter family protein | CHLREDRAFT_186500 | CHLREDRAFT_186500 | estExt_fgenesh2_pg.C_70143 | 4.70E-03 | -1.01E+01 |
| Locus_1636_8Transcript_1/1_Confidence_1.000_Length_2774 | Cre01.g011100.t1.1 | cycloartenol synthase 1 | CHLREDRAFT_196409 | CAS1 | BTM_e_gwW.1.10.1 | 1.24E-07 | -3.72E+00 |
| Locus_1328_1Transcript_1/1_Confidence_1.000_Length_2000 | Cre01.g006950.t1.2 | Aldolase superfamily protein | CHLREDRAFT_152892 | FBA1 | Chlre2_kg.scaffold_52000060 | 1.28E-02 | -2.14E+00 |
| Locus_10313_6Transcript_1/1_Confidence_1.000_Length_1764 | Cre01.g005150.t1.1 | alanine:glyoxylate aminotransferase | CHLREDRAFT_194609 | SGA1a | estExt_fgenesh2_pg.C_720023 | 7.94E-04 | -4.68E+00 |
| Locus_13021_2Transcript_3/3_Confidence_0.600_Length_1222 | Cre01.g005000.t1.2 | NA | CHLREDRAFT_185795 | CPLD9 | estExt_fgenesh2_kg.C_720011 | 4.95E-03 | 2.89E+00 |
| Locus_5983_9Transcript_1/1_Confidence_1.000_Length_723 | Cre01.g004450.t1.1 | NA | CHLREDRAFT_206091 | CPLD42 | OVA_acegs_kg.scaffold_72000022 | 2.80E-09 | -1.10E+01 |
| Locus_278_7Transcript_1/1_Confidence_1.000_Length_2495 | Cre01.g004300.t1.1 | glutamine-dependent asparagine synthase 1 | CHLREDRAFT_140252 | CHLREDRAFT_140252 | estExt_gwp_1W.C_720013 | 7.21E-04 | -2.82E+00 |
| Locus_2015_4Transcript_1/1_Confidence_1.000_Length_796 | CHLREDRAFT_144472 |  | CHLREDRAFT_144472 | CHLREDRAFT_144472 | Chlre2_kg.scaffold_11000017 | 1.88E-03 | -2.32E+00 |

(b) Dt_v11 analysis

| **Dt_name** | **FDR** | **Fold-Change** | **BLAST ID (ncbi_proteinID)** | **Hypothetical function** | **E value** | **KEGG gene name** | **KO** |
| --- | --- | --- | --- | --- | --- | --- | --- |
| TRINITY1_DN12101_c0_g1_i1 | 4.75E-08 | 5.04E+00 | XP_005844749.1 | malate dehydrogenase, cytoplasmic [Chlorella variabilis] | 1.00E-114 | cvr:CHLNCDRAFT_32420 | K00025 |
| TRINITY1_DN9289_c0_g2_i1 | 1.21E-07 | 3.34E+00 | XP_001696240.1 | NAD malic enzyme [Chlamydomonas reinhardtii] | 0.00E+00 | cre:CHLREDRAFT_196833 | K00028 |
| TRINITY1_DN19933_c1_g1_i1 | 9.96E-09 | 2.40E+00 | XP_001694857.1 | isocitrate dehydrogenase, NAD-dependent [Chlamydomonas reinhardtii] | 1.00E-152 | cre:CHLREDRAFT_196044 | K00030 |
| TRINITY2_DN26840_c2_g1_i5 | 0.00E+00 | 2.77E+00 | XP_002953022.1 | hypothetical protein VOLCADRAFT_82038 [Volvox carteri f. nagariensis] | 0.00E+00 | vcn:VOLCADRAFT_82038 | K00036 |
| comp18016_c0_seq2 | 0.00E+00 | 3.98E+00 | XP_005852006.1 | hypothetical protein CHLNCDRAFT_56437 [Chlorella variabilis] | 0.00E+00 | cvr:CHLNCDRAFT_56437 | K00058 |
| Locus_6333_4Transcript_1/1_Confidence_1.000_Length_1596 | 0.00E+00 | 5.63E+02 | XP_001703199.1 | glyceraldehyde 3-phosphate dehydrogenase, dominant splicing variant[Chlamydomonas reinhardtii] | 1.00E-134 | cre:CHLREDRAFT_140618 | K00134 |
| comp8150_c0_seq1 | 0.00E+00 | 3.00E+00 | XP_002950899.1 | E1 subunit of 2-oxoglutarate dehydrogenase [Volvox carteri f.nagariensis] | 0.00E+00 | vcn:VOLCADRAFT_74810 | K00164 |
| Locus_3924_6Transcript_2/2_Confidence_0.909_Length_2549 | 0.00E+00 | 4.97E+00 | XP_002949632.1 | succinate dehydrogenase subunit A, mitochondrial [Volvox carteri f.nagariensis] | 0.00E+00 | vcn:VOLCADRAFT_74298 | K00234 |
| Locus_3473_8Transcript_1/1_Confidence_1.000_Length_1139 | 2.22E-16 | 9.18E+00 | XP_001696290.1 | iron-sulfur subunit of mitochondrial succinate dehydrogenase[Chlamydomonas reinhardtii] | 1.00E-123 | cre:CHLREDRAFT_142231 | K00235 |
| TRINITY2_DN13890_c0_g2_i1 | 0.00E+00 | 1.37E+01 | XP_001689506.1 | succinate dehydrogenase subunit b560 [Chlamydomonas reinhardtii] | 5.00E-52 | cre:CHLREDRAFT_195641 | K00236 |
| Locus_8064_3Transcript_1/1_Confidence_1.000_Length_778 | 6.52E-05 | 4.88E+00 | XP_005651006.1 | hypothetical protein COCSUDRAFT_52340 [Coccomyxa subellipsoideaC-169] | 5.00E-17 | csl:COCSUDRAFT_52340 | K00237 |
| TRINITY2_DN22127_c0_g1_i1 | 0.00E+00 | 5.51E+00 | XP_001699420.1 | dihydropryrimidine dehydrogenase [Chlamydomonas reinhardtii] | 1.00E-147 | cre:CHLREDRAFT_152239 | K00254 |
| TRINITY2_DN21046_c1_g2_i3 | 0.00E+00 | 2.39E+01 | XP_002958237.1 | hypothetical protein VOLCADRAFT_108038 [Volvox carteri f.nagariensis] | 0.00E+00 | vcn:VOLCADRAFT_108038 | K00264 |
| comp25902_c3_seq1 | 0.00E+00 | 1.53E+01 | XP_002959097.1 | copper amine oxidase [Volvox carteri f. nagariensis] | 0.00E+00 | vcn:VOLCADRAFT_84774 | K00276 |
| TRINITY2_DN27291_c0_g1_i1 | 0.00E+00 | 3.78E+00 | XP_002948803.1 | hypothetical protein VOLCADRAFT_104014 [Volvox carteri f.nagariensis] | 0.00E+00 | vcn:VOLCADRAFT_104014 | K00392 |
| TRINITY1_DN11629_c0_g1_i1 | 0.00E+00 | 5.28E+00 | XP_001763239.1 | ferritin, chloroplast precursor [Physcomitrella patens] | 3.00E-69 | ppp:PHYPADRAFT_210187 | K00522 |
| TRINITY2_DN23478_c0_g3_i1 | 2.67E-02 | 5.95E+00 | XP_001690847.1 | ketopantoate hydroxymethyltransferase [Chlamydomonas reinhardtii] | 1.00E-126 | cre:CHLREDRAFT_196344 | K00606 |
| TRINITY1_DN23256_c3_g1_i1 | 0.00E+00 | 2.67E+00 | XP_005650790.1 | pyruvate dehydrogenase [Coccomyxa subellipsoidea C-169] | 1.00E-140 | csl:COCSUDRAFT_26915 | K00627 |
| comp18044_c1_seq2 | 6.88E-05 | 2.75E+00 | XP_005651068.1 | dihydrolipoamide succinyltransferase [Coccomyxa subellipsoidea C-169] | 1.00E-121 | csl:COCSUDRAFT_39599 | K00658 |
| TRINITY2_DN482_c0_g1_i1 | 0.00E+00 | 1.05E+01 | XP_002957433.1 | hypothetical protein VOLCADRAFT_107688 [Volvox carteri f.nagariensis] | 0.00E+00 | vcn:VOLCADRAFT_107688 | K00688 |
| comp33728_c0_seq1 | 0.00E+00 | 2.61E+00 | XP_002946716.1 | hypothetical protein VOLCADRAFT_56349 [Volvox carteri f. nagariensis] | 0.00E+00 | vcn:VOLCADRAFT_56349 | K00705 |
| TRINITY2_DN23795_c0_g2_i5 | 3.28E-06 | 3.75E+00 | XP_002946490.1 | hypothetical protein VOLCADRAFT_120326 [Volvox carteri f.nagariensis] | 1.00E-170 | vcn:VOLCADRAFT_120326 | K00814 |
| comp34291_c0_seq1 | 0.00E+00 | 2.41E+00 | XP_002953045.1 | hypothetical protein VOLCADRAFT_105759 [Volvox carteri f.nagariensis] | 0.00E+00 | vcn:VOLCADRAFT_105759 | K00820 |
| comp31974_c0_seq1 | 0.00E+00 | 2.75E+00 | XP_002950741.1 | hypothetical protein VOLCADRAFT_81234 [Volvox carteri f. nagariensis] | 1.00E-160 | vcn:VOLCADRAFT_81234 | K00831 |
| Locus_825_2Transcript_1/2_Confidence_0.889_Length_2524 | 0.00E+00 | 4.32E+00 | XP_002949213.1 | phosphofructokinase family protein [Volvox carteri f. nagariensis] | 0.00E+00 | vcn:VOLCADRAFT_74177 | K00850 |
| comp26080_c0_seq4 | 8.88E-16 | 3.08E+00 | XP_001700866.1 | ATP-NAD kinase [Chlamydomonas reinhardtii] | 1.00E-158 | cre:CHLREDRAFT_196779 | K00858 |
| TRINITY1_DN60094_c0_g1_i1 | 0.00E+00 | 2.50E+00 | XP_001693008.1 | pyruvate kinase [Chlamydomonas reinhardtii] | 0.00E+00 | cre:CHLREDRAFT_136854 | K00873 |
| TRINITY2_DN57709_c0_g1_i1 | 0.00E+00 | 4.26E+00 | XP_002953017.1 | hypothetical protein VOLCADRAFT_82029 [Volvox carteri f. nagariensis] | 1.00E-158 | vcn:VOLCADRAFT_82029 | K00948 |
| TRINITY2_DN76670_c0_g1_i1 | 0.00E+00 | 4.57E+00 | XP_002950615.1 | hypothetical protein VOLCADRAFT_74670 [Volvox carteri f. nagariensis] | 0.00E+00 | vcn:VOLCADRAFT_74670 | K01006 |
| TRINITY2_DN26678_c0_g3_i8 | 1.11E-02 | 3.38E+00 | XP_005644293.1 | bifunctional 6-phosphofructo-2-kinase/fructose-2,6-bisphosphate2-phosphatase [Coccomyxa subellipsoidea C-169] | 1.00E-158 | csl:COCSUDRAFT_19224 | K01103 |
| TRINITY2_DN20207_c0_g3_i1 | 5.22E-03 | 2.13E+00 | XP_002947011.1 | S-like RNase [Volvox carteri f. nagariensis] | 5.00E-55 | vcn:VOLCADRAFT_103238 | K01166 |
| TRINITY2_DN26326_c3_g11_i1 | 1.38E-11 | 2.26E+00 | XP_005847652.1 | hypothetical protein CHLNCDRAFT_31136 [Chlorella variabilis] | 0.00E+00 | cvr:CHLNCDRAFT_31136 | K01176 |
| comp40486_c0_seq1 | 1.32E-09 | 2.77E+00 | XP_001699454.1 | isoamylase, starch debranching enzyme [Chlamydomonas reinhardtii] | 0.00E+00 | cre:CHLREDRAFT_132067 | K01214 |
| TRINITY1_DN30246_c0_g1_i1 | 1.02E-14 | 5.04E+00 | XP_002956431.1 | fumarate hydratase [Volvox carteri f. nagariensis] | 0.00E+00 | vcn:VOLCADRAFT_77105 | K01676 |
| Locus_139_9Transcript_1/1_Confidence_1.000_Length_3630 | 0.00E+00 | 6.47E+00 | XP_002953876.1 | mitochondrial aconitate hydratase [Volvox carteri f. nagariensis] | 0.00E+00 | vcn:VOLCADRAFT_76010 | K01681 |
| TRINITY2_DN25660_c4_g1_i1 | 0.00E+00 | 6.66E+00 | XP_002957711.1 | hypothetical protein VOLCADRAFT_77661 [Volvox carteri f. nagariensis] | 0.00E+00 | vcn:VOLCADRAFT_77661 | K01687 |
| TRINITY1_DN55213_c0_g1_i1 | 0.00E+00 | 3.85E+00 | XP_002947550.1 | hypothetical protein VOLCADRAFT_87784 [Volvox carteri f. nagariensis] | 0.00E+00 | vcn:VOLCADRAFT_87784 | K01756 |
| comp36689_c0_seq1 | 0.00E+00 | 4.03E+00 | XP_001691581.1 | succinate-coa ligase beta chain [Chlamydomonas reinhardtii] | 1.00E-169 | cre:CHLREDRAFT_24101 | K01900 |
| Locus_96_4Transcript_1/1_Confidence_1.000_Length_1881 | 0.00E+00 | 2.84E+00 | XP_002956198.1 | hypothetical protein VOLCADRAFT_77041 [Volvox carteri f. nagariensis] | 1.00E-173 | vcn:VOLCADRAFT_77041 | K01915 |
| TRINITY1_DN50438_c0_g1_i1 | 0.00E+00 | 2.77E+00 | XP_002958251.1 | hypothetical protein VOLCADRAFT_77870 [Volvox carteri f. nagariensis] | 0.00E+00 | vcn:VOLCADRAFT_77870 | K01955 |
| Locus_3809_9Transcript_1/1_Confidence_1.000_Length_2486 | 0.00E+00 | 2.71E+00 | XP_002951595.1 | hypothetical protein VOLCADRAFT_81499 [Volvox carteri f. nagariensis] | 1.00E-175 | vcn:VOLCADRAFT_81499 | K01956 |
| TRINITY2_DN23401_c2_g1_i1 | 0.00E+00 | 3.22E+00 | XP_002946091.1 | pyruvate carboxylase [Volvox carteri f. nagariensis] | 0.00E+00 | vcn:VOLCADRAFT_102707 | K01958 |
| TRINITY2_DN37157_c0_g1_i1 | 0.00E+00 | 2.81E+00 | XP_002949461.1 | hypothetical protein VOLCADRAFT_109721 [Volvox carteri f.nagariensis] | 2.00E-52 | vcn:VOLCADRAFT_109721 | K02137 |
| TRINITY2_DN76770_c0_g1_i1 | 0.00E+00 | 2.24E+00 | XP_002949803.1 | component of cytosolic 80S ribosome and 60S large subunit [Volvoxcarteri f. nagariensis] | 1.00E-114 | vcn:VOLCADRAFT_109737 | K02932 |
| TRINITY1_DN14230_c4_g1_i2 | 0.00E+00 | 2.16E+00 | XP_002950895.1 | component of cytosolic 80S ribosome and 60S large subunit [Volvoxcarteri f. nagariensis] | 1.00E-110 | vcn:VOLCADRAFT_74803 | K02938 |
| comp208580_c0_seq1 | 0.00E+00 | 2.27E+00 | XP_001690424.1 | ribosomal protein S13, component of cytosolic 80S ribosome and 40Ssmall subunit [Chlamydomonas reinhardtii] | 2.00E-67 | cre:CHLREDRAFT_169450 | K02953 |
| TRINITY2_DN30076_c0_g1_i1 | 0.00E+00 | 2.05E+00 | XP_002946223.1 | component of cytosolic 80S ribosome and 40S small subunit [Volvoxcarteri f. nagariensis] | 7.00E-63 | vcn:VOLCADRAFT_78888 | K02964 |
| TRINITY1_DN47977_c0_g5_i7 | 1.73E-02 | 2.28E+00 | XP_011399024.1 | Elongation factor 1-alpha [Auxenochlorella protothecoides] | 2.00E-75 | apro:F751_6279 | K03231 |
| TRINITY2_DN16646_c0_g1_i2 | 0.00E+00 | 2.05E+00 | XP_005647408.1 | elongation factor EF-3 [Coccomyxa subellipsoidea C-169] | 0.00E+00 | csl:COCSUDRAFT_47721 | K03235 |
| Locus_1228_9Transcript_1/1_Confidence_1.000_Length_2086 | 0.00E+00 | 2.05E+00 | XP_001691452.1 | eukaryotic initiation factor 4A-like protein [Chlamydomonasreinhardtii] | 0.00E+00 | cre:CHLREDRAFT_188942 | K03257 |
| TRINITY2_DN46837_c0_g1_i1 | 0.00E+00 | 2.33E+00 | XP_002947450.1 | hypothetical protein VOLCADRAFT_79605 [Volvox carteri f. nagariensis] | 0.00E+00 | vcn:VOLCADRAFT_79605 | K03301 |
| TRINITY1_DN46861_c5_g4_i1 | 4.16E-02 | 4.53E+00 | XP_001693464.1 | ammonium transporter [Chlamydomonas reinhardtii] | 1.00E-146 | cre:CHLREDRAFT_156131 | K03320 |
| comp28140_c0_seq4 | 2.77E-05 | 3.68E+00 | XP_005843746.1 | hypothetical protein CHLNCDRAFT_139828 [Chlorella variabilis] | 8.00E-77 | cvr:CHLNCDRAFT_139828 | K03386 |
| comp30770_c0_seq26 | 0.00E+00 | 1.25E+01 | XP_001698633.1 | ClpD chaperone, Hsp100 family [Chlamydomonas reinhardtii] | 0.00E+00 | cre:CHLREDRAFT_195417 | K03696 |
| comp35157_c0_seq1 | 3.19E-02 | 2.35E+00 | XP_002950455.1 | catalase [Volvox carteri f. nagariensis] | 0.00E+00 | vcn:VOLCADRAFT_74635 | K03781 |
| comp33344_c0_seq1 | 0.00E+00 | 2.21E+00 | XP_002954591.1 | NADH:ubiquinone oxidoreductase 39 kDa subunit [Volvox carteri f.nagariensis] | 1.00E-125 | vcn:VOLCADRAFT_106450 | K03953 |
| comp31186_c4_seq1 | 1.17E-04 | 2.41E+00 | XP_002948304.1 | mitogen-activated protein kinase 3 [Volvox carteri f. nagariensis] | 1.00E-162 | vcn:VOLCADRAFT_80080 | K04371 |
| TRINITY1_DN9037_c1_g1_i1 | 0.00E+00 | 4.84E+00 | XP_001697267.1 | half-size ABC transporter, membrane protein, partial [Chlamydomonasreinhardtii] | 0.00E+00 | cre:CHLREDRAFT_105113 | K05663 |
| TRINITY1_DN52881_c2_g1_i1 | 0.00E+00 | 2.45E+00 | XP_006855242.1 | PREDICTED: ADP,ATP carrier protein 1, mitochondrial [Amborellatrichopoda] | 1.00E-139 | atr:18445032 | K05863 |
| TRINITY1_DN32850_c1_g2_i1 | 0.00E+00 | 2.38E+00 | XP_002953231.1 | hypothetical protein VOLCADRAFT_93958 [Volvox carteri f. nagariensis] | 0.00E+00 | vcn:VOLCADRAFT_93958 | K06185 |
| TRINITY2_DN25698_c0_g3_i4 | 0.00E+00 | 2.09E+00 | XP_005644898.1 | target of rapamycin kinase [Coccomyxa subellipsoidea C-169] | 0.00E+00 | csl:COCSUDRAFT_30586 | K07203 |
| comp30443_c4_seq3 | 0.00E+00 | 3.03E+00 | XP_001700833.1 | R1 protein, alpha-glucan water dikinase [Chlamydomonas reinhardtii] | 0.00E+00 | cre:CHLREDRAFT_205870 | K08244 |
| TRINITY2_DN25845_c2_g4_i5 | 0.00E+00 | 3.86E+00 | XP_005646486.1 | kinase-like protein, partial [Coccomyxa subellipsoidea C-169] | 1.00E-130 | csl:COCSUDRAFT_17062 | K08287 |
| Locus_8689_9Transcript_1/1_Confidence_1.000_Length_467 | 6.71E-03 | 5.74E+00 | XP_001417250.1 | predicted protein [Ostreococcus lucimarinus CCE9901] | 4.00E-41 | olu:OSTLU_15043 | K08738 |
| TRINITY1_DN6539_c0_g1_i2 | 5.34E-03 | 2.30E+00 | XP_002953255.1 | hypothetical protein VOLCADRAFT_63528 [Volvox carteri f. nagariensis] | 1.00E-130 | vcn:VOLCADRAFT_63528 | K08850 |
| TRINITY1_DN26539_c6_g1_i1 | 0.00E+00 | 2.56E+00 | XP_001695303.1 | predicted protein [Chlamydomonas reinhardtii] | 1.00E-148 | cre:CHLREDRAFT_138087 | K09645 |
| TRINITY1_DN28377_c1_g1_i1 | 0.00E+00 | 2.09E+00 | XP_001699742.1 | dynein heavy chain 5 [Chlamydomonas reinhardtii] | 0.00E+00 | cre:CHLREDRAFT_126477 | K10408 |
| TRINITY2_DN68294_c0_g1_i1 | 6.77E-08 | 2.66E+00 | XP_002956139.1 | hypothetical protein VOLCADRAFT_107069 [Volvox carteri f.nagariensis] | 2.00E-84 | vcn:VOLCADRAFT_107069 | K11279 |
| TRINITY1_DN44799_c2_g1_i3 | 4.95E-02 | 1.58E+01 | XP_001691938.1 | protein arginine N-methyltransferase, partial [Chlamydomonasreinhardtii] | 3.00E-84 | cre:CHLREDRAFT_115641 | K11436 |
| Locus_3136_4Transcript_1/1_Confidence_1.000_Length_1097 | 0.00E+00 | 2.77E+00 | XP_001697748.1 | hypothetical protein CHLREDRAFT_192781 [Chlamydomonas reinhardtii] | 3.00E-25 | cre:CHLREDRAFT_192781 | K12382 |
| TRINITY1_DN13169_c0_g1_i1 | 0.00E+00 | 2.43E+00 | XP_005844200.1 | hypothetical protein CHLNCDRAFT_59028 [Chlorella variabilis] | 1.00E-124 | cvr:CHLNCDRAFT_59028 | K13126 |
| TRINITY1_DN50530_c0_g1_i1 | 0.00E+00 | 3.30E+01 | XP_001693482.1 | predicted protein [Chlamydomonas reinhardtii] | 0.00E+00 | cre:CHLREDRAFT_182699 | K13412 |
| TRINITY1_DN45185_c2_g3_i2 | 1.05E-02 | 7.25E+00 | XP_006356907.2 | PREDICTED: putative receptor-like protein kinase At3g47110 [Solanumtuberosum] | 1.00E-17 | sot:102583440 | K13420 |
| comp31670_c0_seq1 | 0.00E+00 | 2.36E+00 | XP_001698065.1 | receptor of activated protein kinase C 1 [Chlamydomonas reinhardtii] | 1.00E-164 | cre:CHLREDRAFT_105734 | K14753 |
| TRINITY2_DN25739_c0_g2_i6 | 3.82E-02 | 2.57E+00 | XP_002949914.1 | hypothetical protein VOLCADRAFT_104545 [Volvox carteri f.nagariensis] | 1.00E-115 | vcn:VOLCADRAFT_104545 | K14772 |
| Locus_8314_10Transcript_4/4_Confidence_0.500_Length_1838 | 1.19E-06 | 2.46E+00 | XP_002955898.1 | hypothetical protein VOLCADRAFT_96796 [Volvox carteri f. nagariensis] | 2.00E-89 | vcn:VOLCADRAFT_96796 | K14816 |
| TRINITY1_DN29624_c0_g1_i1 | 2.66E-02 | 2.51E+00 | XP_002947359.1 | hypothetical protein VOLCADRAFT_87659 [Volvox carteri f. nagariensis] | 1.00E-165 | vcn:VOLCADRAFT_87659 | K14832 |
| TRINITY1_DN48670_c0_g1_i1 | 3.90E-02 | 5.80E+00 | XP_002953398.1 | pumilio family protein [Volvox carteri f. nagariensis] | 1.00E-124 | vcn:VOLCADRAFT_121154 | K14844 |
| TRINITY2_DN13519_c0_g1_i1 | 2.22E-04 | 2.79E+00 | XP_001696593.1 | mitochondrial substrate carrier protein [Chlamydomonas reinhardtii] | 1.00E-127 | cre:CHLREDRAFT_155742 | K15100 |
| comp7833_c0_seq1 | 0.00E+00 | 2.36E+00 | XP_001689726.1 | triose phosphate translocator [Chlamydomonas reinhardtii] | 1.00E-138 | cre:CHLREDRAFT_205633 | K15283 |
| TRINITY2_DN25197_c1_g1_i2 | 1.61E-02 | 2.28E+00 | XP_002954407.1 | hypothetical protein VOLCADRAFT_64703 [Volvox carteri f. nagariensis] | 1.00E-170 | vcn:VOLCADRAFT_64703 | K15631 |
| TRINITY1_DN50575_c0_g1_i1 | 0.00E+00 | 3.15E+00 | XP_001696211.1 | phosphoglycerate mutase [Chlamydomonas reinhardtii] | 0.00E+00 | cre:CHLREDRAFT_196305 | K15633 |
| TRINITY2_DN25668_c0_g2_i5 | 3.26E-03 | 2.82E+00 | XP_005850711.1 | hypothetical protein CHLNCDRAFT_50422 [Chlorella variabilis] | 1.00E-89 | cvr:CHLNCDRAFT_50422 | K15710 |
| TRINITY1_DN33365_c0_g1_i1 | 1.66E-09 | 3.45E+00 | XP_005643160.1 | DEAD-domain-containing protein [Coccomyxa subellipsoidea C-169] | 1.00E-120 | csl:COCSUDRAFT_31548 | K17679 |
| TRINITY2_DN24727_c1_g5_i3 | 5.53E-08 | 2.66E+00 | XP_002950383.1 | hypothetical protein VOLCADRAFT_90709 [Volvox carteri f. nagariensis] | 1.00E-129 | vcn:VOLCADRAFT_90709 | K17893 |
| TRINITY1_DN28709_c1_g3_i1 | 2.42E-03 | 2.67E+00 | XP_005649988.1 | WD40 repeat-like protein, partial [Coccomyxa subellipsoidea C-169] | 2.00E-64 | csl:COCSUDRAFT_3880 | K17908 |
| Locus_1373_1Transcript_1/2_Confidence_0.750_Length_1449 | 6.10E-03 | 3.38E+00 | XP_001695319.1 | subunit of retromer complex [Chlamydomonas reinhardtii] | 1.00E-131 | cre:CHLREDRAFT_191646 | K17917 |
| TRINITY2_DN20493_c0_g2_i1 | 4.26E-12 | 4.38E+00 | XP_002952713.1 | hypothetical protein VOLCADRAFT_93375 [Volvox carteri f. nagariensis] | 1.00E-180 | vcn:VOLCADRAFT_93375 | K18010 |
| TRINITY2_DN17878_c0_g3_i1 | 5.42E-03 | 2.11E+00 | XP_001692322.1 | hypothetical protein CHLREDRAFT_171044, partial [Chlamydomonasreinhardtii] | 1.00E-144 | cre:CHLREDRAFT_171044 | K18550 |
| TRINITY1_DN47314_c0_g3_i12 | 3.39E-03 | 3.91E+00 | XP_002946729.1 | hypothetical protein VOLCADRAFT_120362, partial [Volvox carteri f.nagariensis] | 0.00E+00 | vcn:VOLCADRAFT_120362 | K20093 |
| Locus_4313_3Transcript_2/2_Confidence_0.941_Length_4731 | 7.34E-11 | 2.00E+00 | XP_001701917.1 | predicted protein [Chlamydomonas reinhardtii] | 0.00E+00 | cre:CHLREDRAFT_141404 |  |
| Locus_2022_9Transcript_1/1_Confidence_1.000_Length_1299 | 1.87E-08 | 2.01E+00 | XP_001697656.1 | NADH:ubiquinone oxidoreductase ND4L subunit [Chlamydomonasreinhardtii] | 3.00E-36 | cre:CHLREDRAFT_58686 |  |
| Locus_768_9Transcript_3/4_Confidence_0.810_Length_8632 | 0.00E+00 | 2.01E+00 | YP_005089767.1 | ycf1 gene product (chloroplast) [Dunaliella salina] | 0.00E+00 |  |  |
| Locus_4_10Transcript_29/30_Confidence_0.280_Length_8790 | 6.58E-06 | 2.02E+00 | WP_002193500.1 | group II intron reverse transcriptase/maturase [Bacillus cereus] | 2.00E-98 |  |  |
| Locus_1061_2Transcript_2/10_Confidence_0.329_Length_2146 | 1.27E-03 | 2.02E+00 | XP_005846096.1 | hypothetical protein CHLNCDRAFT_135985 [Chlorella variabilis] | 3.00E-13 | cvr:CHLNCDRAFT_135985 |  |
| TRINITY1_DN48198_c0_g1_i1 | 2.74E-04 | 2.02E+00 | XP_002955565.1 | hypothetical protein VOLCADRAFT_106853 [Volvox carteri f.nagariensis] | 2.00E-17 | vcn:VOLCADRAFT_106853 |  |
| TRINITY1_DN14086_c0_g7_i1 | 1.05E-02 | 2.03E+00 | XP_001690290.1 | predicted protein [Chlamydomonas reinhardtii] | 0.00E+00 | cre:CHLREDRAFT_129367 |  |
| TRINITY1_DN45177_c12_g1_i1 | 0.00E+00 | 2.04E+00 | XP_013901230.1 | 60S acidic ribosomal protein P0 [Monoraphidium neglectum] | 1.00E-105 |  |  |
| Locus_1000000_gi\|371532890\|gb\|JQ039079.1\| | 4.41E-14 | 2.06E+00 | YP_005089779.1 | rps12 gene product (chloroplast) [Dunaliella salina] | 3.00E-60 |  |  |
| Locus_1000000_gi\|371532920\|gb\|JQ039095.1\| | 5.12E-09 | 2.06E+00 | YP_005089822.1 | ycf4 gene product (chloroplast) [Dunaliella salina] | 1.00E-108 |  |  |
| Locus_7_9Transcript_45/54_Confidence_0.043_Length_3908 | 1.60E-12 | 2.08E+00 | WP_041347641.1 | group II intron reverse transcriptase/maturase [Clostridiumbotulinum] | 2.00E-79 |  |  |
| TRINITY2_DN3671_c0_g1_i1 | 1.77E-02 | 2.10E+00 | XP_001698210.1 | hypothetical protein CHLREDRAFT_193133, partial [Chlamydomonasreinhardtii] | 1.00E-11 | cre:CHLREDRAFT_193133 |  |
| Locus_60_10Transcript_7/8_Confidence_0.560_Length_3169 | 0.00E+00 | 2.11E+00 | YP_005089789.1 | orf316 gene product (chloroplast) [Dunaliella salina] | 1.00E-127 |  |  |
| Locus_483_4Transcript_1/1_Confidence_1.000_Length_798 | 0.00E+00 | 2.12E+00 | XP_013906666.1 | 60S ribosomal protein L11 [Monoraphidium neglectum] | 4.00E-76 |  |  |
| Locus_6270_10Transcript_1/1_Confidence_1.000_Length_583 | 5.41E-10 | 2.13E+00 | YP_009185281.1 | putative GIY-YIG homing endonuclease (chloroplast) [Phacotuslenticularis] | 1.00E-10 |  |  |
| TRINITY2_DN24355_c0_g1_i2 | 1.50E-02 | 2.18E+00 | WP_040355446.1 | alpha/beta hydrolase [[Clostridium] ultunense] | 1.00E-14 |  |  |
| comp21929_c1_seq1 | 5.76E-04 | 2.25E+00 | XP_011401450.1 | putative membrane protein [Auxenochlorella protothecoides] | 7.00E-65 | apro:F751_5348 |  |
| TRINITY2_DN24393_c0_g2_i1 | 0.00E+00 | 2.26E+00 | YP_007507309.1 | ATP synthase CF0 C subunit (chloroplast) [Gonium pectorale] | 1.00E-22 |  |  |
| Locus_551_3Transcript_3/3_Confidence_0.750_Length_1522 | 1.91E-05 | 2.27E+00 | XP_002947742.1 | hypothetical protein VOLCADRAFT_103542 [Volvox carteri f.nagariensis] | 2.00E-20 | vcn:VOLCADRAFT_103542 |  |
| Locus_1000000_gi\|371532904\|gb\|JQ039086.1\| | 0.00E+00 | 2.28E+00 | YP_005089818.1 | rps7 gene product (chloroplast) [Dunaliella salina] | 1.00E-86 |  |  |
| TRINITY2_DN27455_c0_g1_i1 | 3.00E-11 | 2.28E+00 | XP_001692507.1 | predicted protein [Chlamydomonas reinhardtii] | 5.00E-24 | cre:CHLREDRAFT_189510 |  |
| TRINITY2_DN27366_c0_g1_i1 | 0.00E+00 | 2.30E+00 | YP_005089783.1 | chlL gene product (chloroplast) [Dunaliella salina] | 1.00E-129 |  |  |
| Locus_2509_3Transcript_3/4_Confidence_0.154_Length_2037 | 1.89E-02 | 2.30E+00 | XP_002953446.1 | hypothetical protein VOLCADRAFT_94261 [Volvox carteri f. nagariensis] | 2.00E-30 | vcn:VOLCADRAFT_94261 |  |
| Locus_10144_10Transcript_4/5_Confidence_0.636_Length_1360 | 0.00E+00 | 2.32E+00 | YP_005089784.1 | orf435 gene product (chloroplast) [Dunaliella salina] | 7.00E-42 |  |  |
| Locus_394_9Transcript_4/7_Confidence_0.516_Length_4095 | 3.23E-09 | 2.34E+00 | YP_005089840.1 | rpoBa gene product (chloroplast) [Dunaliella salina] | 0.00E+00 |  |  |
| comp27530_c0_seq1 | 2.49E-04 | 2.39E+00 | XP_002499876.1 | SNF2 super family [Micromonas sp. RCC299] | 1.00E-177 | mis:MICPUN_56506 |  |
| Locus_4753_10Transcript_1/1_Confidence_1.000_Length_1116 | 1.68E-13 | 2.41E+00 | XP_002952842.1 | hypothetical protein VOLCADRAFT_81971 [Volvox carteri f.nagariensis] | 6.00E-78 | vcn:VOLCADRAFT_81971 |  |
| Locus_2893_7Transcript_1/1_Confidence_1.000_Length_2898 | 2.69E-02 | 2.42E+00 | XP_002957260.1 | hypothetical protein VOLCADRAFT_121617 [Volvox carteri f.nagariensis] | 6.00E-91 | vcn:VOLCADRAFT_121617 |  |
| Locus_72_1Transcript_1/10_Confidence_0.375_Length_2182 | 2.80E-07 | 2.43E+00 | YP_009105759.1 | CP43 chlorophyll apoprotein of photosystem II (chloroplast) [Koliellacorcontica] | 3.00E-54 |  |  |
| comp34574_c0_seq1 | 7.78E-12 | 2.44E+00 | YP_005089798.1 | rpl2 gene product (chloroplast) [Dunaliella salina] | 1.00E-157 |  |  |
| Locus_4386_10Transcript_3/4_Confidence_0.667_Length_1378 | 0.00E+00 | 2.44E+00 | XP_001703471.1 | mitochondrial phosphate carrier 1 [Chlamydomonas reinhardtii] | 1.00E-131 |  |  |
| TRINITY2_DN14398_c0_g1_i1 | 4.18E-02 | 2.46E+00 | XP_005849597.1 | hypothetical protein CHLNCDRAFT_143048 [Chlorella variabilis] | 1.00E-18 | cvr:CHLNCDRAFT_143048 |  |
| Locus_4_2Transcript_29/36_Confidence_0.094_Length_3129 | 1.32E-10 | 2.50E+00 | YP_009130602.1 | photosystem II protein D1 (chloroplast) [Tydemania expeditionis] | 3.00E-34 |  |  |
| TRINITY2_DN21549_c0_g3_i1 | 0.00E+00 | 2.53E+00 | WP_046380201.1 | hypothetical protein [Rhodococcus erythropolis] | 2.00E-12 |  |  |
| TRINITY2_DN25197_c1_g1_i1 | 8.66E-06 | 2.54E+00 | XP_013897984.1 | hypothetical protein MNEG_8999 [Monoraphidium neglectum] | 0.00E+00 |  |  |
| TRINITY1_DN46511_c0_g1_i2 | 7.29E-06 | 2.56E+00 | XP_002950578.1 | hypothetical protein VOLCADRAFT_104805 [Volvox carteri f.nagariensis] | 1.00E-37 | vcn:VOLCADRAFT_104805 |  |
| TRINITY1_DN46282_c0_g3_i8 | 3.70E-05 | 2.57E+00 | XP_013904236.1 | Calvin cycle protein CP12 [Monoraphidium neglectum] | 4.00E-18 |  |  |
| TRINITY1_DN45688_c0_g1_i1 | 1.72E-02 | 2.57E+00 | XP_013902633.1 | hypothetical protein MNEG_4346 [Monoraphidium neglectum] | 2.00E-62 |  |  |
| Locus_1000000_gi\|371532874\|gb\|JQ039071.1\| | 2.09E-14 | 2.58E+00 | YP_005089768.1 | rpl16 gene product (chloroplast) [Dunaliella salina] | 2.00E-72 |  |  |
| TRINITY1_DN42832_c1_g2_i1 | 5.68E-03 | 2.58E+00 | XP_002946954.1 | hypothetical protein VOLCADRAFT_120385 [Volvox carteri f.nagariensis] | 2.00E-77 | vcn:VOLCADRAFT_120385 |  |
| TRINITY1_DN31285_c1_g3_i1 | 1.01E-08 | 2.58E+00 | XP_002958327.1 | hypothetical protein VOLCADRAFT_108077 [Volvox carteri f.nagariensis] | 1.00E-150 | vcn:VOLCADRAFT_108077 |  |
| Locus_14562_2Transcript_1/1_Confidence_1.000_Length_745 | 2.97E-02 | 2.58E+00 | XP_013905416.1 | hypothetical protein MNEG_1558 [Monoraphidium neglectum] | 5.00E-17 |  |  |
| Locus_1000000_gi\|371532822\|gb\|JQ039045.1\| | 1.11E-16 | 2.59E+00 | YP_005089774.1 | petA gene product, partial (chloroplast) [Dunaliella salina] | 1.00E-144 |  |  |
| TRINITY2_DN24309_c0_g2_i5 | 3.30E-02 | 2.61E+00 | XP_001690608.1 | hypothetical protein CHLREDRAFT_182946 [Chlamydomonas reinhardtii] | 4.00E-26 | cre:CHLREDRAFT_182946 |  |
| TRINITY1_DN57995_c0_g1_i1 | 0.00E+00 | 2.61E+00 | XP_002957246.1 | hypothetical protein VOLCADRAFT_84030 [Volvox carteri f. nagariensis] | 9.00E-87 | vcn:VOLCADRAFT_84030 |  |
| comp32024_c0_seq1 | 0.00E+00 | 2.61E+00 | XP_013899564.1 | putative Ribosome biogeneis protein NSA2 like protein [Monoraphidiumneglectum] | 1.00E-127 |  |  |
| Locus_1000000_gi\|371532806\|gb\|JQ039037.1\| | 0.00E+00 | 2.64E+00 | YP_005089781.1 | atpI gene product (chloroplast) [Dunaliella salina] | 1.00E-105 |  |  |
| Locus_4_7Transcript_62/805_Confidence_1.000_Length_963 | 7.88E-11 | 2.64E+00 | YP_005089808.1 | orf235 gene product (chloroplast) [Dunaliella salina] | 1.00E-120 |  |  |
| Locus_1000000_gi\|371532882\|gb\|JQ039075.1\| | 7.77E-16 | 2.66E+00 | YP_005089770.1 | rpl5 gene product (chloroplast) [Dunaliella salina] | 2.00E-95 |  |  |
| TRINITY1_DN47057_c2_g3_i3 | 1.17E-02 | 2.75E+00 | XP_005845297.1 | hypothetical protein CHLNCDRAFT_137023 [Chlorella variabilis] | 5.00E-80 | cvr:CHLNCDRAFT_137023 |  |
| Locus_1000000_gi\|371532902\|gb\|JQ039085.1\| | 8.55E-10 | 2.78E+00 | YP_005089790.1 | rps4 gene product (chloroplast) [Dunaliella salina] | 1.00E-139 |  |  |
| Locus_5568_3Transcript_2/6_Confidence_0.308_Length_2506 | 3.21E-11 | 2.78E+00 | XP_001703717.1 | cysteine endopeptidase [Chlamydomonas reinhardtii] | 1.00E-102 | cre:CHLREDRAFT_22333 |  |
| Locus_5280_6Transcript_1/1_Confidence_1.000_Length_1017 | 5.80E-03 | 2.79E+00 | XP_006305523.1 | hypothetical protein CARUB_v10009994mg, partial [Capsella rubella] | 6.00E-47 |  |  |
| comp331318_c0_seq1 | 0.00E+00 | 2.79E+00 | YP_005089817.1 | rps14 gene product (chloroplast) [Dunaliella salina] | 1.00E-49 |  |  |
| TRINITY2_DN26983_c2_g6_i1 | 1.35E-03 | 2.82E+00 | XP_002958131.1 | hypothetical protein VOLCADRAFT_99335 [Volvox carteri f. nagariensis] | 1.00E-146 | vcn:VOLCADRAFT_99335 |  |
| Locus_1216_3Transcript_2/10_Confidence_0.378_Length_2309 | 0.00E+00 | 2.83E+00 | XP_005847655.1 | hypothetical protein CHLNCDRAFT_134022 [Chlorella variabilis] | 8.00E-30 | cvr:CHLNCDRAFT_134022 |  |
| TRINITY1_DN35291_c0_g5_i2 | 4.39E-03 | 2.85E+00 | WP_036687632.1 | hypothetical protein [Paenibacillus sp. MSt1] | 4.00E-19 |  |  |
| TRINITY2_DN2995_c0_g2_i1 | 1.22E-14 | 2.86E+00 | XP_011396787.1 | Collagen alpha-6(VI) chain [Auxenochlorella protothecoides] | 8.00E-13 | apro:F751_6877 |  |
| Locus_1_8Transcript_30/31_Confidence_0.388_Length_3792 | 3.09E-09 | 2.87E+00 | WP_036155815.1 | fasciclin [Maribacter forsetii] | 4.00E-18 |  |  |
| comp17796_c0_seq3 | 5.18E-09 | 2.88E+00 | XP_001694525.1 | sugar/carbohydrate kinase [Chlamydomonas reinhardtii] | 5.00E-89 | cre:CHLREDRAFT_205628 |  |
| TRINITY1_DN47526_c10_g7_i1 | 0.00E+00 | 2.89E+00 | XP_002958643.1 | hypothetical protein VOLCADRAFT_78003 [Volvox carteri f. nagariensis] | 1.00E-160 | vcn:VOLCADRAFT_78003 |  |
| TRINITY1_DN52999_c0_g1_i1 | 3.69E-14 | 3.01E+00 | XP_002956904.1 | hypothetical protein VOLCADRAFT_119562 [Volvox carteri f.nagariensis] | 1.00E-100 | vcn:VOLCADRAFT_119562 |  |
| Locus_333_5Transcript_2/2_Confidence_0.875_Length_1320 | 7.61E-12 | 3.02E+00 | XP_001697011.1 | hypothetical protein CHLREDRAFT_150727, partial [Chlamydomonasreinhardtii] | 2.00E-45 | cre:CHLREDRAFT_150727 |  |
| TRINITY1_DN45061_c1_g2_i6 | 3.97E-02 | 3.04E+00 | XP_002502564.1 | predicted protein [Micromonas sp. RCC299] | 2.00E-54 | mis:MICPUN_108291 |  |
| TRINITY2_DN25475_c0_g1_i1 | 0.00E+00 | 3.08E+00 | XP_001697221.1 | chloroplast processing enzyme [Chlamydomonas reinhardtii] | 0.00E+00 | cre:CHLREDRAFT_206035 |  |
| Locus_1000000_gi\|371532800\|gb\|JQ039034.1\| | 0.00E+00 | 3.14E+00 | YP_005089819.1 | atpE gene product (chloroplast) [Dunaliella salina] | 8.00E-69 |  |  |
| TRINITY2_DN24483_c0_g1_i5 | 5.23E-11 | 3.15E+00 | XP_002951217.1 | hypothetical protein VOLCADRAFT_61085, partial [Volvox carteri f.nagariensis] | 0.00E+00 | vcn:VOLCADRAFT_61085 |  |
| Locus_1738_6Transcript_1/1_Confidence_1.000_Length_936 | 6.32E-07 | 3.17E+00 | XP_002952928.1 | hypothetical protein VOLCADRAFT_82034 [Volvox carteri f.nagariensis] | 4.00E-46 | vcn:VOLCADRAFT_82034 |  |
| Locus_3660_2Transcript_3/3_Confidence_0.667_Length_1765 | 1.04E-02 | 3.20E+00 | XP_009415879.1 | PREDICTED: probable metal-nicotianamine transporter YSL6 isoform X1[Musa acuminata subsp. malaccensis] | 2.00E-86 | mus:103996649 |  |
| comp541119_c0_seq1 | 8.17E-04 | 3.22E+00 | XP_013895504.1 | 40S ribosomal protein S15a-1 [Monoraphidium neglectum] | 4.00E-20 |  |  |
| Locus_12_5Transcript_20/24_Confidence_0.013_Length_619 | 0.00E+00 | 3.24E+00 | YP_003058333.1 | ATP synthase CF0 C subunit (chloroplast) [Parachlorella kessleri] | 1.00E-11 |  |  |
| TRINITY1_DN50815_c0_g1_i1 | 2.31E-09 | 3.25E+00 | XP_001699139.1 | hypothetical protein CHLREDRAFT_185295 [Chlamydomonas reinhardtii] | 2.00E-54 | cre:CHLREDRAFT_185295 |  |
| TRINITY1_DN3629_c0_g1_i1 | 2.19E-12 | 3.26E+00 | XP_002951804.1 | hypothetical protein VOLCADRAFT_92325 [Volvox carteri f. nagariensis] | 1.00E-144 | vcn:VOLCADRAFT_92325 |  |
| Locus_1000000_gi\|371532872\|gb\|JQ039070.1\| | 1.17E-14 | 3.26E+00 | YP_005089769.1 | rpl14 gene product (chloroplast) [Dunaliella salina] | 2.00E-58 |  |  |
| Locus_13_3Transcript_49/51_Confidence_0.074_Length_1226 | 0.00E+00 | 3.28E+00 | YP_636210.1 | putative site-specific DNA endonuclease (chloroplast)[Pseudendoclonium akinetum] | 1.00E-10 |  |  |
| comp9012_c0_seq1 | 1.33E-15 | 3.29E+00 | XP_001695847.1 | hypothetical protein CHLREDRAFT_149177 [Chlamydomonas reinhardtii] | 1.00E-134 | cre:CHLREDRAFT_149177 |  |
| TRINITY2_DN26853_c0_g3_i2 | 3.87E-03 | 3.36E+00 | XP_002946517.1 | R-SNARE, Tomsyn-like family [Volvox carteri f. nagariensis] | 1.00E-81 | vcn:VOLCADRAFT_86534 |  |
| Locus_3089_4Transcript_1/1_Confidence_1.000_Length_983 | 1.43E-09 | 3.40E+00 | XP_002950714.1 | hypothetical protein VOLCADRAFT_91185 [Volvox carteri f.nagariensis] | 5.00E-43 | vcn:VOLCADRAFT_91185 |  |
| TRINITY1_DN9146_c1_g1_i1 | 2.98E-04 | 3.41E+00 | XP_013901672.1 | glutamine amidotransferase [Monoraphidium neglectum] | 6.00E-85 |  |  |
| Locus_10562_7Transcript_1/2_Confidence_0.667_Length_720 | 1.74E-03 | 3.41E+00 | XP_001698177.1 | hypothetical protein CHLREDRAFT_151685, partial [Chlamydomonasreinhardtii] | 1.00E-16 | cre:CHLREDRAFT_151685 |  |
| Locus_1702_8Transcript_4/6_Confidence_0.400_Length_1745 | 4.07E-12 | 3.42E+00 | XP_002945729.1 | hypothetical protein VOLCADRAFT_85997 [Volvox carteri f. nagariensis] | 7.00E-64 | vcn:VOLCADRAFT_85997 |  |
| TRINITY1_DN22472_c2_g1_i1 | 1.52E-11 | 3.49E+00 | XP_005644013.1 | hypothetical protein COCSUDRAFT_67904 [Coccomyxa subellipsoideaC-169] | 6.00E-96 | csl:COCSUDRAFT_67904 |  |
| Locus_200_2Transcript_5/5_Confidence_0.617_Length_4424 | 0.00E+00 | 3.56E+00 | YP_005089838.1 | chlN gene product (chloroplast) [Dunaliella salina] | 0.00E+00 |  |  |
| TRINITY1_DN14134_c0_g14_i1 | 0.00E+00 | 3.62E+00 | XP_001702057.1 | predicted protein [Chlamydomonas reinhardtii] | 6.00E-32 | cre:CHLREDRAFT_179628 |  |
| Locus_1062_8Transcript_1/1_Confidence_1.000_Length_4619 | 0.00E+00 | 3.62E+00 | XP_002948938.1 | hypothetical protein VOLCADRAFT_89311 [Volvox carteri f. nagariensis] | 0.00E+00 | vcn:VOLCADRAFT_89311 |  |
| TRINITY1_DN1977_c0_g1_i1 | 1.14E-03 | 3.63E+00 | XP_001702212.1 | hypothetical protein CHLREDRAFT_179827, partial [Chlamydomonasreinhardtii] | 1.00E-152 | cre:CHLREDRAFT_179827 |  |
| Locus_5196_10Transcript_2/4_Confidence_0.500_Length_1576 | 0.00E+00 | 3.69E+00 | XP_002955692.1 | hypothetical protein VOLCADRAFT_121443 [Volvox carteri f.nagariensis] | 6.00E-89 | vcn:VOLCADRAFT_121443 |  |
| Locus_5807_8Transcript_1/1_Confidence_1.000_Length_1618 | 3.21E-06 | 3.74E+00 | XP_001693275.1 | chloroplast RNA polymerase sigma factor, c [Chlamydomonasreinhardtii] | 6.00E-62 | cre:CHLREDRAFT_190458 |  |
| TRINITY2_DN3117_c0_g2_i1 | 0.00E+00 | 3.77E+00 | YP_005089791.1 | rpl20 gene product (chloroplast) [Dunaliella salina] | 2.00E-55 |  |  |
| TRINITY1_DN48001_c4_g1_i5 | 0.00E+00 | 3.77E+00 | XP_002951341.1 | hypothetical protein VOLCADRAFT_91916 [Volvox carteri f. nagariensis] | 0.00E+00 | vcn:VOLCADRAFT_91916 |  |
| TRINITY1_DN3968_c0_g1_i1 | 1.06E-13 | 3.81E+00 | YP_005089835.1 | orf222 gene product (chloroplast) [Dunaliella salina] | 2.00E-65 |  |  |
| comp22885_c0_seq1 | 0.00E+00 | 3.90E+00 | XP_001702307.1 | allophanate hydrolase [Chlamydomonas reinhardtii] | 9.00E-92 | cre:CHLREDRAFT_196482 |  |
| TRINITY2_DN25932_c1_g3_i1 | 1.88E-03 | 3.91E+00 | WP_007099706.1 | sarcosine oxidase [Synechococcus sp. RS9916] | 4.00E-64 |  |  |
| TRINITY2_DN21118_c0_g1_i3 | 1.32E-02 | 3.93E+00 | XP_002953800.1 | hypothetical protein VOLCADRAFT_94642 [Volvox carteri f.nagariensis] | 1.00E-31 | vcn:VOLCADRAFT_94642 |  |
| TRINITY2_DN17046_c0_g1_i1 | 4.36E-03 | 3.93E+00 | XP_011401319.1 | Developmentally-regulated GTP-binding protein 1 [Auxenochlorellaprotothecoides] | 3.00E-98 | apro:F751_3815 |  |
| TRINITY2_DN26616_c0_g3_i10 | 0.00E+00 | 4.16E+00 | XP_001693619.1 | PAS domain protein [Chlamydomonas reinhardtii] | 0.00E+00 | cre:CHLREDRAFT_143717 |  |
| TRINITY2_DN76789_c0_g1_i1 | 0.00E+00 | 4.18E+00 | XP_001693109.1 | succinyl-CoA ligase [Chlamydomonas reinhardtii] | 1.00E-126 |  |  |
| Locus_3206_1Transcript_2/2_Confidence_0.667_Length_1352 | 9.26E-04 | 4.22E+00 | XP_002957601.1 | hypothetical protein VOLCADRAFT_98692 [Volvox carteri f.nagariensis] | 1.00E-19 | vcn:VOLCADRAFT_98692 |  |
| Locus_4_7Transcript_97/805_Confidence_1.000_Length_792 | 0.00E+00 | 4.23E+00 | YP_009185017.1 | putative LAGLIDADG homing endonuclease (chloroplast) [Hafniomonaslaevis] | 2.00E-28 |  |  |
| TRINITY1_DN52854_c0_g1_i1 | 0.00E+00 | 4.26E+00 | XP_002946291.1 | hypothetical protein VOLCADRAFT_115858 [Volvox carteri f.nagariensis] | 1.00E-179 | vcn:VOLCADRAFT_115858 |  |
| Locus_2864_5Transcript_1/4_Confidence_0.750_Length_1316 | 8.73E-03 | 4.30E+00 | XP_002946960.1 | hypothetical protein VOLCADRAFT_87088 [Volvox carteri f. nagariensis] | 2.00E-13 | vcn:VOLCADRAFT_87088 |  |
| Locus_1000000_gi\|371532794\|gb\|JQ039031.1\| | 0.00E+00 | 4.37E+00 | YP_005089809.1 | orf231 gene product (chloroplast) [Dunaliella salina] | 1.00E-128 |  |  |
| TRINITY1_DN8537_c0_g2_i1 | 0.00E+00 | 4.56E+00 | XP_002946134.1 | hypothetical protein VOLCADRAFT_127387 [Volvox carteri f.nagariensis] | 5.00E-23 | vcn:VOLCADRAFT_127387 |  |
| Locus_3834_5Transcript_1/1_Confidence_1.000_Length_1917 | 4.10E-02 | 4.57E+00 | XP_001699166.1 | hypothetical protein CHLREDRAFT_196080 [Chlamydomonas reinhardtii] | 2.00E-91 | cre:CHLREDRAFT_196080 |  |
| comp26099_c0_seq3 | 7.78E-08 | 4.68E+00 | XP_001702869.1 | adenylate kinase 1 [Chlamydomonas reinhardtii] | 1.00E-163 | cre:CHLREDRAFT_194947 |  |
| TRINITY2_DN77259_c0_g1_i1 | 0.00E+00 | 4.69E+00 | YP_005089785.1 | clpP gene product (chloroplast) [Dunaliella salina] | 0.00E+00 |  |  |
| TRINITY2_DN5457_c0_g1_i1 | 0.00E+00 | 4.71E+00 | XP_002955191.1 | hypothetical protein VOLCADRAFT_121401 [Volvox carteri f.nagariensis] | 1.00E-143 | vcn:VOLCADRAFT_121401 |  |
| TRINITY2_DN23365_c0_g1_i1 | 3.81E-03 | 4.74E+00 | XP_005644706.1 | hypothetical protein COCSUDRAFT_34056 [Coccomyxa subellipsoideaC-169] | 7.00E-50 | csl:COCSUDRAFT_34056 |  |
| TRINITY2_DN27663_c0_g1_i1 | 6.35E-06 | 4.76E+00 | XP_013903247.1 | glutamine synthetase [Monoraphidium neglectum] | 2.00E-44 |  |  |
| Locus_3515_4Transcript_1/1_Confidence_1.000_Length_674 | 1.98E-06 | 4.85E+00 | XP_002947978.1 | hypothetical protein VOLCADRAFT_116549, partial [Volvox carteri f.nagariensis] | 6.00E-56 | vcn:VOLCADRAFT_116549 |  |
| comp6352_c0_seq1 | 0.00E+00 | 4.87E+00 | XP_001698369.1 | thioredoxin-like protein [Chlamydomonas reinhardtii] | 2.00E-20 | cre:CHLREDRAFT_195752 |  |
| comp24137_c0_seq3 | 1.40E-02 | 4.95E+00 | XP_013901297.1 | hypothetical protein MNEG_5682 [Monoraphidium neglectum] | 4.00E-43 |  |  |
| TRINITY1_DN427_c0_g1_i1 | 1.01E-03 | 4.98E+00 | XP_002954759.1 | hypothetical protein VOLCADRAFT_95657 [Volvox carteri f. nagariensis] | 4.00E-57 | vcn:VOLCADRAFT_95657 |  |
| Locus_8548_6Transcript_1/1_Confidence_1.000_Length_1066 | 6.07E-04 | 5.03E+00 | XP_005646513.1 | WD40 repeat-like protein [Coccomyxa subellipsoidea C-169] | 7.00E-50 | csl:COCSUDRAFT_29904 |  |
| TRINITY1_DN52932_c0_g1_i3 | 2.36E-13 | 5.06E+00 | XP_002947747.1 | glycine-rich RNA-binding protein-like protein [Volvox carteri f.nagariensis] | 9.00E-26 | vcn:VOLCADRAFT_103546 |  |
| TRINITY1_DN48217_c0_g1_i1 | 1.10E-03 | 5.55E+00 | XP_005844867.1 | hypothetical protein CHLNCDRAFT_138376 [Chlorella variabilis] | 0.00E+00 | cvr:CHLNCDRAFT_138376 |  |
| TRINITY2_DN47109_c0_g8_i1 | 3.64E-11 | 5.82E+00 | XP_001700192.1 | predicted protein [Chlamydomonas reinhardtii] | 3.00E-63 | cre:CHLREDRAFT_194028 |  |
| TRINITY1_DN188_c0_g1_i1 | 1.46E-02 | 5.84E+00 | XP_001689706.1 | predicted protein [Chlamydomonas reinhardtii] | 2.00E-85 | cre:CHLREDRAFT_190047 |  |
| comp30959_c1_seq6 | 7.85E-03 | 5.89E+00 | XP_010550854.1 | PREDICTED: DNA mismatch repair protein MSH1, mitochondrial [Tarenayahassleriana] | 8.00E-42 | thj:104821629 |  |
| Locus_9920_7Transcript_1/1_Confidence_1.000_Length_807 | 1.75E-03 | 6.02E+00 | XP_002958418.1 | hypothetical protein VOLCADRAFT_99692 [Volvox carteri f.nagariensis] | 4.00E-38 | vcn:VOLCADRAFT_99692 |  |
| Locus_141_8Transcript_5/5_Confidence_0.627_Length_3210 | 0.00E+00 | 6.04E+00 | YP_005089837.1 | rpoA gene product (chloroplast) [Dunaliella salina] | 0.00E+00 |  |  |
| Locus_1368_10Transcript_22/31_Confidence_0.286_Length_1720 | 0.00E+00 | 6.34E+00 | WP_053991722.1 | hypothetical protein [Mangrovimonas sp. TPBH4] | 1.00E-10 |  |  |
| comp26865_c0_seq4 | 0.00E+00 | 6.58E+00 | XP_013903958.1 | putative plastid-lipid-associated protein 8, chloroplastic[Monoraphidium neglectum] | 2.00E-39 |  |  |
| TRINITY2_DN66967_c0_g1_i1 | 0.00E+00 | 6.59E+00 | XP_002958653.1 | hypothetical protein VOLCADRAFT_108237 [Volvox carteri f.nagariensis] | 1.00E-27 | vcn:VOLCADRAFT_108237 |  |
| TRINITY2_DN26162_c0_g1_i4 | 9.68E-05 | 6.72E+00 | XP_011399795.1 | hypothetical protein F751_0779 [Auxenochlorella protothecoides] | 1.00E-103 | apro:F751_0779 |  |
| TRINITY2_DN6393_c0_g1_i1 | 1.07E-04 | 6.73E+00 | XP_013900344.1 | hypothetical protein MNEG_6636 [Monoraphidium neglectum] | 5.00E-77 |  |  |
| TRINITY1_DN47399_c6_g5_i14 | 2.00E-15 | 6.80E+00 | XP_001701627.1 | guanylate cyclase [Chlamydomonas reinhardtii] | 8.00E-42 | cre:CHLREDRAFT_166115 |  |
| Locus_939_9Transcript_17/22_Confidence_0.473_Length_3168 | 0.00E+00 | 6.94E+00 | WP_010519784.1 | fasciclin [Mesoflavibacter zeaxanthinifaciens] | 3.00E-18 |  |  |
| TRINITY1_DN13894_c1_g2_i1 | 1.78E-15 | 7.10E+00 | XP_001695777.1 | hypothetical protein CHLREDRAFT_174408, partial [Chlamydomonasreinhardtii] | 5.00E-38 | cre:CHLREDRAFT_174408 |  |
| Locus_466_5Transcript_3/4_Confidence_0.600_Length_1639 | 0.00E+00 | 7.50E+00 | XP_001693648.1 | predicted protein [Chlamydomonas reinhardtii] | 1.00E-104 | cre:CHLREDRAFT_187832 |  |
| TRINITY1_DN45747_c4_g1_i3 | 0.00E+00 | 7.69E+00 | XP_002951009.1 | hypothetical protein VOLCADRAFT_104960 [Volvox carteri f.nagariensis] | 0.00E+00 | vcn:VOLCADRAFT_104960 |  |
| TRINITY1_DN47488_c0_g1_i12 | 5.11E-06 | 8.08E+00 | XP_011091752.1 | PREDICTED: uncharacterized protein LOC105172113 [Sesamum indicum] | 7.00E-77 | sind:105172113 |  |
| TRINITY2_DN26754_c0_g1_i5 | 0.00E+00 | 8.32E+00 | XP_001691551.1 | flagellar associated membrane protein [Chlamydomonas reinhardtii] | 0.00E+00 | cre:CHLREDRAFT_145154 |  |
| TRINITY2_DN26502_c0_g2_i6 | 9.93E-04 | 8.40E+00 | XP_013898957.1 | hypothetical protein MNEG_8026 [Monoraphidium neglectum] | 4.00E-72 |  |  |
| TRINITY1_DN41474_c0_g1_i18 | 6.39E-04 | 8.87E+00 | XP_001691384.1 | sugar nucleotide transporter [Chlamydomonas reinhardtii] | 2.00E-77 | cre:CHLREDRAFT_144907 |  |
| Locus_38493_9Transcript_1/1_Confidence_1.000_Length_1329 | 0.00E+00 | 9.14E+00 | XP_013900993.1 | hypothetical protein MNEG_5984 [Monoraphidium neglectum] | 5.00E-83 |  |  |
| TRINITY1_DN50899_c0_g1_i1 | 0.00E+00 | 9.43E+00 | XP_003057041.1 | predicted protein [Micromonas pusilla CCMP1545] | 2.00E-24 | mpp:MICPUCDRAFT_44138 |  |
| TRINITY1_DN28597_c0_g2_i4 | 6.89E-05 | 1.00E+01 | XP_001689558.1 | DEAD/DEAH-box helicase, partial [Chlamydomonas reinhardtii] | 1.00E-158 | cre:CHLREDRAFT_116679 |  |
| TRINITY2_DN25467_c1_g1_i1 | 0.00E+00 | 1.01E+01 | XP_002948362.1 | hypothetical protein VOLCADRAFT_88626 [Volvox carteri f. nagariensis] | 4.00E-19 | vcn:VOLCADRAFT_88626 |  |
| TRINITY2_DN6231_c0_g1_i1 | 2.07E-02 | 1.02E+01 | XP_013896565.1 | E1A/CREB-binding protein [Monoraphidium neglectum] | 7.00E-16 |  |  |
| Locus_4170_7Transcript_4/4_Confidence_0.625_Length_1528 | 4.76E-02 | 1.18E+01 | XP_002948110.1 | hypothetical protein VOLCADRAFT_103740 [Volvox carteri f.nagariensis] | 4.00E-82 | vcn:VOLCADRAFT_103740 |  |
| Locus_5432_8Transcript_1/1_Confidence_1.000_Length_990 | 0.00E+00 | 1.27E+01 | XP_001695886.1 | hypothetical protein CHLREDRAFT_184434 [Chlamydomonas reinhardtii] | 3.00E-53 | cre:CHLREDRAFT_184434 |  |
| TRINITY1_DN52861_c0_g1_i1 | 0.00E+00 | 1.29E+01 | XP_005647922.1 | hypothetical protein COCSUDRAFT_28806 [Coccomyxa subellipsoideaC-169] | 3.00E-99 | csl:COCSUDRAFT_28806 |  |
| Locus_12602_7Transcript_1/1_Confidence_1.000_Length_1982 | 0.00E+00 | 1.33E+01 | XP_005843676.1 | hypothetical protein CHLNCDRAFT_140067 [Chlorella variabilis] | 1.00E-134 | cvr:CHLNCDRAFT_140067 |  |
| Locus_894_7Transcript_11/11_Confidence_0.429_Length_1685 | 0.00E+00 | 1.48E+01 | XP_002950414.1 | hypothetical protein VOLCADRAFT_74587 [Volvox carteri f. nagariensis] | 2.00E-98 | vcn:VOLCADRAFT_74587 |  |
| comp31127_c5_seq1 | 3.33E-16 | 1.64E+01 | XP_013903980.1 | hypothetical protein MNEG_2999 [Monoraphidium neglectum] | 1.00E-105 |  |  |
| TRINITY2_DN3665_c0_g1_i1 | 0.00E+00 | 1.76E+01 | XP_002957311.1 | hypothetical protein VOLCADRAFT_98356 [Volvox carteri f. nagariensis] | 0.00E+00 | vcn:VOLCADRAFT_98356 |  |
| TRINITY1_DN39349_c1_g1_i17 | 1.13E-05 | 2.12E+01 | WP_051512250.1 | hypothetical protein [Skermanella stibiiresistens] | 1.00E-40 |  |  |
| TRINITY2_DN26412_c0_g1_i2 | 1.96E-02 | 2.27E+01 | XP_013898354.1 | GPI transamidase component PIG-T [Monoraphidium neglectum] | 2.00E-35 |  |  |
| TRINITY1_DN39349_c1_g1_i8 | 3.15E-08 | 3.00E+01 | XP_005643341.1 | hypothetical protein COCSUDRAFT_20416 [Coccomyxa subellipsoideaC-169] | 1.00E-47 | csl:COCSUDRAFT_20416 |  |
| TRINITY1_DN44339_c2_g2_i1 | 9.50E-03 | 3.92E+01 | XP_012575125.1 | PREDICTED: uncharacterized protein LOC101508115 [Cicer arietinum] | 1.00E-113 | cam:101508115 |  |
| Locus_220_10Transcript_1/1_Confidence_1.000_Length_5406 | 7.62E-05 | 4.23E+01 | WP_040977597.1 | protein A*, partial [Necropsobacter massiliensis] | 0.00E+00 |  |  |
| Locus_5_2Transcript_33/37_Confidence_0.114_Length_7316 | 9.61E-05 | 4.49E+01 | WP_027628829.1 | group II intron reverse transcriptase/maturase [[Clostridium]cellobioparum] | 1.00E-45 |  |  |
| Locus_3038_1Transcript_1/7_Confidence_0.267_Length_1420 | 0.00E+00 | 5.94E+01 | XP_005645091.1 | urea active transporter-like protein [Coccomyxa subellipsoideaC-169] | 2.00E-95 | csl:COCSUDRAFT_30678 |  |
| TRINITY1_DN42814_c0_g1_i2 | 0.00E+00 | 7.75E+01 | XP_002957274.1 | hypothetical protein VOLCADRAFT_107613 [Volvox carteri f.nagariensis] | 0.00E+00 | vcn:VOLCADRAFT_107613 |  |
| TRINITY2_DN24779_c1_g4_i1 | 0.00E+00 | 7.99E+01 | XP_013904340.1 | putative MFS transporter, AGZA family, xanthine/uracil permease[Monoraphidium neglectum] | 1.00E-133 |  |  |
| TRINITY1_DN8534_c0_g2_i1 | 0.00E+00 | 1.01E+02 | XP_002951533.1 | hypothetical protein VOLCADRAFT_92124 [Volvox carteri f. nagariensis] | 1.00E-113 | vcn:VOLCADRAFT_92124 |  |
| comp36085_c0_seq1 | 0.00E+00 | 3.23E-01 | XP_001703473.1 | hypothetical protein CHLREDRAFT_114298 [Chlamydomonas reinhardtii] | 1.00E-111 | cre:CHLREDRAFT_114298 | K00059 |
| comp43407_c0_seq1 | 6.38E-08 | 3.66E-01 | XP_002953640.1 | hypothetical protein VOLCADRAFT_82431 [Volvox carteri f. nagariensis] | 0.00E+00 | vcn:VOLCADRAFT_82431 | K00099 |
| comp37019_c0_seq1 | 7.76E-13 | 4.30E-01 | XP_002950542.1 | hypothetical protein VOLCADRAFT_104783 [Volvox carteri f.nagariensis] | 1.00E-167 | vcn:VOLCADRAFT_104783 | K00161 |
| TRINITY2_DN27305_c0_g1_i1 | 0.00E+00 | 3.42E-01 | XP_002957188.1 | hypothetical protein VOLCADRAFT_77470 [Volvox carteri f. nagariensis] | 1.00E-159 | vcn:VOLCADRAFT_77470 | K00162 |
| TRINITY1_DN50430_c0_g1_i1 | 0.00E+00 | 2.96E-01 | XP_005852175.1 | hypothetical protein CHLNCDRAFT_59537 [Chlorella variabilis] | 1.00E-133 | cvr:CHLNCDRAFT_59537 | K00208 |
| TRINITY1_DN3624_c1_g3_i1 | 0.00E+00 | 4.43E-02 | XP_001689464.1 | light-dependent protochlorophyllide reductase [Chlamydomonasreinhardtii] | 1.00E-123 | cre:CHLREDRAFT_136589 | K00218 |
| comp40876_c0_seq1 | 0.00E+00 | 1.32E-01 | XP_001701729.1 | coproporphyrinogen III oxidase [Chlamydomonas reinhardtii] | 1.00E-163 | cre:CHLREDRAFT_53583 | K00228 |
| TRINITY1_DN60169_c0_g1_i1 | 0.00E+00 | 4.37E-01 | XP_001692993.1 | glycine cleavage system, P protein [Chlamydomonas reinhardtii] | 0.00E+00 | cre:CHLREDRAFT_136984 | K00281 |
| Locus_591_3Transcript_4/7_Confidence_0.727_Length_5170 | 0.00E+00 | 7.19E-02 | XP_002955216.1 | hypothetical protein VOLCADRAFT_83067 [Volvox carteri f. nagariensis] | 0.00E+00 | vcn:VOLCADRAFT_83067 | K00366 |
| TRINITY1_DN55434_c0_g1_i1 | 0.00E+00 | 1.49E-01 | XP_001689807.1 | NADPH-dependent thioredoxin reductase [Chlamydomonas reinhardtii] | 1.00E-159 | cre:CHLREDRAFT_78928 | K00384 |
| Locus_3379_9Transcript_1/1_Confidence_1.000_Length_1255 | 0.00E+00 | 3.44E-01 | XP_002951957.1 | hypothetical protein VOLCADRAFT_92469 [Volvox carteri f.nagariensis] | 6.00E-67 | vcn:VOLCADRAFT_92469 | K00510 |
| comp43721_c0_seq1 | 0.00E+00 | 2.20E-01 | XP_001693107.1 | glycine cleavage system, T protein [Chlamydomonas reinhardtii] | 1.00E-146 | cre:CHLREDRAFT_196242 | K00605 |
| comp6073_c0_seq1 | 3.00E-15 | 2.83E-01 | XP_002949800.1 | hypothetical protein VOLCADRAFT_104457 [Volvox carteri f.nagariensis] | 1.00E-136 | vcn:VOLCADRAFT_104457 | K00645 |
| Locus_43614_10Transcript_4/4_Confidence_0.769_Length_1176 | 0.00E+00 | 1.92E-01 | XP_002951559.1 | hypothetical protein VOLCADRAFT_92181 [Volvox carteri f.nagariensis] | 6.00E-79 | vcn:VOLCADRAFT_92181 | K00761 |
| TRINITY1_DN57678_c0_g1_i1 | 1.88E-02 | 3.97E-01 | XP_001693168.1 | farnesyl diphosphate synthase [Chlamydomonas reinhardtii] | 1.00E-147 | cre:CHLREDRAFT_137019 | K00787 |
| TRINITY2_DN24700_c0_g1_i1 | 0.00E+00 | 2.39E-01 | XP_001696661.1 | S-Adenosylmethionine synthetase [Chlamydomonas reinhardtii] | 0.00E+00 | cre:CHLREDRAFT_182408 | K00789 |
| Locus_2721_5Transcript_1/1_Confidence_1.000_Length_853 | 0.00E+00 | 1.68E-01 | XP_002946387.1 | hypothetical protein VOLCADRAFT_108879 [Volvox carteri f.nagariensis] | 1.00E-61 | vcn:VOLCADRAFT_108879 | K00794 |
| TRINITY1_DN60022_c0_g1_i1 | 0.00E+00 | 1.35E-01 | XP_001702942.1 | 5-enolpyruvylshikimate-3-phosphate synthase [Chlamydomonasreinhardtii] | 0.00E+00 | cre:CHLREDRAFT_133088 | K00800 |
| TRINITY1_DN48108_c0_g2_i1 | 0.00E+00 | 1.77E-01 | XP_002949266.1 | hypothetical protein VOLCADRAFT_74092 [Volvox carteri f. nagariensis] | 0.00E+00 | vcn:VOLCADRAFT_74092 | K00830 |
| TRINITY2_DN67368_c0_g1_i1 | 0.00E+00 | 4.22E-01 | XP_002949322.1 | hypothetical protein VOLCADRAFT_104265 [Volvox carteri f.nagariensis] | 1.00E-162 | vcn:VOLCADRAFT_104265 | K00855 |
| comp32205_c0_seq1 | 0.00E+00 | 2.02E-01 | XP_001699523.1 | phosphoglycerate kinase [Chlamydomonas reinhardtii] | 1.00E-157 | cre:CHLREDRAFT_132210 | K00927 |
| TRINITY1_DN4954_c0_g1_i1 | 2.19E-04 | 3.51E-01 | XP_002949468.1 | adenylate kinase [Volvox carteri f. nagariensis] | 1.00E-100 | vcn:VOLCADRAFT_89730 | K00939 |
| TRINITY1_DN60364_c0_g1_i1 | 1.61E-13 | 2.02E-01 | XP_002958218.1 | hypothetical protein VOLCADRAFT_84386 [Volvox carteri f. nagariensis] | 1.00E-80 | vcn:VOLCADRAFT_84386 | K00991 |
| TRINITY2_DN23544_c2_g1_i1 | 0.00E+00 | 3.18E-01 | XP_001693339.1 | S-Adenosyl homocysteine hydrolase [Chlamydomonas reinhardtii] | 0.00E+00 | cre:CHLREDRAFT_129593 | K01251 |
| TRINITY2_DN17097_c0_g1_i1 | 0.00E+00 | 2.45E-01 | XP_001694682.1 | inorganic pyrophosphatase [Chlamydomonas reinhardtii] | 0.00E+00 | cre:CHLREDRAFT_137778 | K01507 |
| comp18300_c0_seq1 | 7.50E-05 | 3.39E-01 | XP_002956240.1 | uroporphyrinogen decarboxylase chloroplast precursor [Volvox carterif. nagariensis] | 1.00E-154 | vcn:VOLCADRAFT_83541 | K01599 |
| Locus_2695_3Transcript_2/2_Confidence_0.750_Length_2093 | 5.33E-15 | 2.93E-01 | XP_002957689.1 | hypothetical protein VOLCADRAFT_84198 [Volvox carteri f. nagariensis] | 2.00E-99 | vcn:VOLCADRAFT_84198 | K01611 |
| TRINITY2_DN37535_c1_g1_i1 | 0.00E+00 | 2.70E-01 | XP_001702062.1 | 1-deoxy-D-xylulose 5-phosphate synthase, partial [Chlamydomonasreinhardtii] | 0.00E+00 | cre:CHLREDRAFT_196568 | K01662 |
| TRINITY2_DN47402_c0_g1_i1 | 0.00E+00 | 1.56E-01 | XP_001701779.1 | delta-aminolevulinic acid dehydratase [Chlamydomonas reinhardtii] | 1.00E-167 | cre:CHLREDRAFT_186639 | K01698 |
| TRINITY2_DN16675_c0_g1_i1 | 0.00E+00 | 2.01E-01 | XP_001696681.1 | uroporphyrinogen-iii synthase [Chlamydomonas reinhardtii] | 2.00E-83 | cre:CHLREDRAFT_195943 | K01719 |
| TRINITY2_DN57167_c0_g1_i1 | 0.00E+00 | 8.30E-02 | XP_002950840.1 | hypothetical protein VOLCADRAFT_85406 [Volvox carteri f. nagariensis] | 1.00E-137 | vcn:VOLCADRAFT_85406 | K01749 |
| Locus_5338_5Transcript_1/1_Confidence_1.000_Length_2543 | 0.00E+00 | 4.46E-01 | XP_001697991.1 | phosphoglucomutase [Chlamydomonas reinhardtii] | 0.00E+00 | cre:CHLREDRAFT_81483 | K01835 |
| comp31999_c0_seq1 | 0.00E+00 | 2.90E-01 | XP_001697519.1 | glutamate-1-semialdehyde aminotransferase [Chlamydomonas reinhardtii] | 0.00E+00 | cre:CHLREDRAFT_138524 | K01845 |
| TRINITY1_DN48250_c2_g1_i1 | 0.00E+00 | 2.99E-01 | XP_001689874.1 | cycloartenol synthase [Chlamydomonas reinhardtii] | 0.00E+00 | cre:CHLREDRAFT_196409 | K01853 |
| TRINITY1_DN50569_c0_g1_i1 | 0.00E+00 | 2.35E-01 | XP_001700965.1 | non-discriminatory gln-glu-trna synthetase [Chlamydomonasreinhardtii] | 0.00E+00 | cre:CHLREDRAFT_195574 | K01885 |
| TRINITY2_DN26802_c0_g1_i2 | 6.71E-11 | 2.98E-01 | XP_001700230.1 | acetyl CoA synthetase [Chlamydomonas reinhardtii] | 0.00E+00 | cre:CHLREDRAFT_139750 | K01895 |
| TRINITY1_DN47623_c5_g1_i1 | 7.68E-03 | 4.75E-01 | XP_002947410.1 | hypothetical protein VOLCADRAFT_79713 [Volvox carteri f. nagariensis] | 0.00E+00 | vcn:VOLCADRAFT_79713 | K01938 |
| TRINITY2_DN13088_c0_g1_i1 | 4.22E-10 | 3.84E-01 | XP_001702113.1 | asparagine synthase [Chlamydomonas reinhardtii] | 0.00E+00 | cre:CHLREDRAFT_140252 | K01953 |
| comp29136_c0_seq7 | 1.07E-04 | 4.70E-01 | XP_002948082.1 | hypothetical protein VOLCADRAFT_120546 [Volvox carteri f.nagariensis] | 1.00E-178 | vcn:VOLCADRAFT_120546 | K01962 |
| TRINITY1_DN55239_c0_g4_i1 | 8.80E-11 | 3.25E-01 | XP_002953562.1 | hypothetical protein VOLCADRAFT_82311 [Volvox carteri f. nagariensis] | 1.00E-154 | vcn:VOLCADRAFT_82311 | K01963 |
| Locus_1501_10Transcript_1/2_Confidence_0.750_Length_1049 | 0.00E+00 | 2.93E-01 | XP_001697332.1 | CF0 ATP synthase subunit II precursor [Chlamydomonas reinhardtii] | 3.00E-48 | cre:CHLREDRAFT_206190 | K02109 |
| Locus_241_4Transcript_1/1_Confidence_1.000_Length_1001 | 0.00E+00 | 4.00E-01 | XP_002957070.1 | ATP synthase, subunit D, chloroplast precursor [Volvox carteri f.nagariensis] | 1.00E-70 | vcn:VOLCADRAFT_77395 | K02113 |
| TRINITY2_DN37060_c0_g1_i1 | 0.00E+00 | 4.64E-01 | XP_002945818.1 | ATP synthase, subunit gamma, chloroplast precursor [Volvox carteri f.nagariensis] | 1.00E-135 | vcn:VOLCADRAFT_102786 | K02115 |
| TRINITY2_DN14628_c0_g2_i1 | 0.00E+00 | 1.28E-01 | XP_005649768.1 | acetyl-CoA biotin carboxyl carrier [Coccomyxa subellipsoidea C-169] | 2.00E-34 | csl:COCSUDRAFT_65159 | K02160 |
| TRINITY2_DN18207_c0_g2_i2 | 3.94E-03 | 3.97E-01 | XP_002947387.1 | minichromosome maintenance protein 7 [Volvox carteri f. nagariensis] | 0.00E+00 | vcn:VOLCADRAFT_87725 | K02210 |
| TRINITY1_DN60050_c0_g3_i1 | 4.17E-05 | 3.20E-01 | XP_002954315.1 | minichromosome maintenance protein 4 [Volvox carteri f. nagariensis] | 0.00E+00 | vcn:VOLCADRAFT_106331 | K02212 |
| Locus_2045_7Transcript_1/1_Confidence_1.000_Length_811 | 5.88E-05 | 1.38E-01 | XP_002946335.1 | hypothetical protein VOLCADRAFT_79012 [Volvox carteri f.nagariensis] | 2.00E-36 | vcn:VOLCADRAFT_79012 | K02221 |
| Locus_20669_9Transcript_1/1_Confidence_1.000_Length_1234 | 5.59E-06 | 4.55E-01 | XP_001693164.1 | predicted protein, partial [Chlamydomonas reinhardtii] | 2.00E-73 | cre:CHLREDRAFT_102512 | K02372 |
| TRINITY2_DN25394_c3_g1_i1 | 0.00E+00 | 2.62E-01 | XP_002951823.1 | hypothetical protein VOLCADRAFT_81610 [Volvox carteri f. nagariensis] | 0.00E+00 | vcn:VOLCADRAFT_81610 | K02492 |
| Locus_1700_6Transcript_1/1_Confidence_1.000_Length_3658 | 3.00E-04 | 3.20E-01 | XP_001700080.1 | minichromosome maintenance protein 2 [Chlamydomonas reinhardtii] | 0.00E+00 | cre:CHLREDRAFT_152683 | K02540 |
| TRINITY1_DN46088_c0_g1_i1 | 6.66E-16 | 3.30E-01 | XP_001691326.1 | minichromosome maintenance protein 3, partial [Chlamydomonasreinhardtii] | 0.00E+00 | cre:CHLREDRAFT_115083 | K02541 |
| TRINITY1_DN43375_c2_g5_i1 | 1.43E-03 | 4.88E-01 | XP_002946873.1 | minichromosome maintenance protein 6 [Volvox carteri f. nagariensis] | 0.00E+00 | vcn:VOLCADRAFT_79263 | K02542 |
| TRINITY2_DN26682_c2_g3_i2 | 0.00E+00 | 2.26E-01 | XP_001694496.1 | nitrate transporter [Chlamydomonas reinhardtii] | 1.00E-122 | cre:CHLREDRAFT_130019 | K02575 |
| TRINITY1_DN45279_c0_g2_i5 | 0.00E+00 | 9.06E-02 | XP_002946905.1 | hypothetical protein VOLCADRAFT_72905 [Volvox carteri f.nagariensis] | 3.00E-47 | vcn:VOLCADRAFT_72905 | K02638 |
| Locus_61_4Transcript_1/1_Confidence_1.000_Length_841 | 6.42E-07 | 2.88E-01 | XP_001702961.1 | apoferredoxin [Chlamydomonas reinhardtii] | 3.00E-83 | cre:CHLREDRAFT_196703 | K02639 |
| TRINITY2_DN27041_c0_g1_i1 | 0.00E+00 | 2.58E-01 | XP_001697352.1 | ferredoxin-nadp reductase [Chlamydomonas reinhardtii] | 1.00E-147 | cre:CHLREDRAFT_195553 | K02641 |
| TRINITY1_DN47352_c0_g3_i8 | 1.09E-08 | 2.52E-01 | XP_001702611.1 | photosystem I 8.1 kDa reaction center subunit IV [Chlamydomonasreinhardtii] | 1.00E-26 | cre:CHLREDRAFT_76146 | K02693 |
| TRINITY1_DN44495_c0_g2_i6 | 0.00E+00 | 4.54E-01 | XP_001696798.1 | photosystem I reaction center subunit III [Chlamydomonasreinhardtii] | 2.00E-59 | cre:CHLREDRAFT_130914 | K02694 |
| TRINITY1_DN47626_c0_g6_i6 | 0.00E+00 | 2.31E-01 | XP_001690629.1 | subunit H of photosystem I [Chlamydomonas reinhardtii] | 1.00E-36 | cre:CHLREDRAFT_182959 | K02695 |
| Locus_3277_10Transcript_1/1_Confidence_1.000_Length_662 | 0.00E+00 | 3.55E-01 | XP_002951752.1 | photosystem I reaction center subunit VIII, chloroplast precursor[Volvox carteri f. nagariensis] | 4.00E-11 | vcn:VOLCADRAFT_105181 | K02696 |
| Locus_477_4Transcript_1/1_Confidence_1.000_Length_1065 | 0.00E+00 | 3.31E-01 | XP_001691084.1 | photosystem I reaction center subunit XI [Chlamydomonasreinhardtii] | 2.00E-69 | cre:CHLREDRAFT_205935 | K02699 |
| comp31597_c0_seq1 | 0.00E+00 | 3.63E-01 | XP_002954867.1 | subunit of oxygen evolving complex of photosystem II [Volvox carterif. nagariensis] | 1.00E-103 | vcn:VOLCADRAFT_83003 | K02716 |
| TRINITY2_DN11727_c0_g2_i1 | 0.00E+00 | 2.16E-01 | XP_001691034.1 | OEE2-like protein of thylakoid lumen [Chlamydomonas reinhardtii] | 1.00E-56 | cre:CHLREDRAFT_183033 | K02717 |
| TRINITY1_DN47800_c1_g3_i4 | 6.83E-05 | 3.35E-01 | XP_001702016.1 | photosystem II reaction center W protein [Chlamydomonasreinhardtii] | 4.00E-21 | cre:CHLREDRAFT_155150 | K02721 |
| comp32823_c0_seq1 | 0.00E+00 | 1.21E-01 | XP_002960025.1 | hypothetical protein VOLCADRAFT_108813, partial [Volvox carteri f.nagariensis] | 1.00E-30 | vcn:VOLCADRAFT_108813 | K02723 |
| TRINITY2_DN11641_c0_g1_i1 | 2.61E-02 | 2.04E-01 | XP_002950109.1 | hypothetical protein VOLCADRAFT_80962 [Volvox carteri f.nagariensis] | 9.00E-56 | vcn:VOLCADRAFT_80962 | K02838 |
| comp35439_c0_seq1 | 3.30E-08 | 3.66E-01 | XP_002946225.1 | plastid/chloroplast ribosomal protein L10 [Volvox carteri f.nagariensis] | 3.00E-69 | vcn:VOLCADRAFT_102827 | K02864 |
| Locus_26509_9Transcript_1/1_Confidence_1.000_Length_727 | 2.79E-02 | 4.03E-01 | XP_002945781.1 | plastid/chloroplast ribosomal protein L13 [Volvox carteri f.nagariensis] | 1.00E-70 | vcn:VOLCADRAFT_78780 | K02871 |
| Locus_7047_10Transcript_1/1_Confidence_1.000_Length_808 | 1.42E-05 | 4.22E-01 | XP_001696120.1 | plastid ribosomal protein L31 [Chlamydomonas reinhardtii] | 1.00E-27 | cre:CHLREDRAFT_195624 | K02909 |
| Locus_6448_7Transcript_1/2_Confidence_0.667_Length_920 | 1.01E-05 | 4.77E-01 | XP_001697380.1 | plastid ribosomal protein L4 [Chlamydomonas reinhardtii] | 4.00E-74 | cre:CHLREDRAFT_185040 | K02926 |
| Locus_1240_8Transcript_1/8_Confidence_0.432_Length_2561 | 8.05E-10 | 3.97E-01 | XP_002949136.1 | hypothetical protein VOLCADRAFT_104207 [Volvox carteri f.nagariensis] | 1.00E-116 | vcn:VOLCADRAFT_104207 | K03103 |
| TRINITY2_DN37144_c0_g1_i1 | 0.00E+00 | 3.28E-01 | XP_002955061.1 | magnesium protoporphyrin chelatase [Volvox carteri f. nagariensis] | 0.00E+00 | vcn:VOLCADRAFT_76537 | K03403 |
| TRINITY2_DN7602_c0_g2_i1 | 0.00E+00 | 2.87E-01 | XP_002956151.1 | magnesium chelatase subunit D chloroplast precursor [Volvox carterif. nagariensis] | 1.00E-141 | vcn:VOLCADRAFT_107092 | K03404 |
| TRINITY1_DN55223_c1_g1_i1 | 0.00E+00 | 2.72E-01 | XP_002947884.1 | magnesium protoporphyrin IX S-adenosyl methionine O-methyltransferase chloroplast precursor [Volvox carteri f.nagariensis] | 1.00E-109 | vcn:VOLCADRAFT_109643 | K03428 |
| comp34862_c0_seq1 | 7.99E-11 | 4.85E-01 | XP_002947297.1 | 4-hydroxy-3-methylbut-2-enyl diphosphate reductase chloroplastprecursor [Volvox carteri f. nagariensis] | 1.00E-174 | vcn:VOLCADRAFT_79586 | K03527 |
| Locus_1499_10Transcript_1/3_Confidence_0.667_Length_503 | 0.00E+00 | 4.29E-01 | XP_002945815.1 | hypothetical protein VOLCADRAFT_109549 [Volvox carteri f.nagariensis] | 2.00E-38 | vcn:VOLCADRAFT_109549 | K03541 |
| Locus_5756_1Transcript_3/3_Confidence_0.778_Length_750 | 3.44E-03 | 3.45E-01 | XP_001702592.1 | thioredoxin dependent peroxidase [Chlamydomonas reinhardtii] | 1.00E-62 | cre:CHLREDRAFT_181913 | K03564 |
| Locus_1463_4Transcript_1/1_Confidence_1.000_Length_815 | 0.00E+00 | 3.37E-01 | XP_001690314.1 | thioredoxin m [Chlamydomonas reinhardtii] | 7.00E-37 | cre:CHLREDRAFT_136413 | K03671 |
| Locus_11516_7Transcript_1/1_Confidence_1.000_Length_963 | 6.37E-05 | 2.93E-01 | XP_002953624.1 | hypothetical protein VOLCADRAFT_109223 [Volvox carteri f.nagariensis] | 6.00E-71 | vcn:VOLCADRAFT_109223 | K03768 |
| TRINITY1_DN52963_c1_g1_i1 | 4.33E-15 | 3.16E-01 | XP_001701588.1 | chlorophyll synthetase [Chlamydomonas reinhardtii] | 1.00E-143 | cre:CHLREDRAFT_5437 | K04040 |
| Locus_4245_3Transcript_4/6_Confidence_0.238_Length_1503 | 8.68E-03 | 2.89E-01 | XP_001699929.1 | hypothetical protein CHLREDRAFT_182152 [Chlamydomonas reinhardtii] | 1.00E-67 | cre:CHLREDRAFT_182152 | K04427 |
| TRINITY1_DN48344_c2_g1_i1 | 1.11E-15 | 3.41E-01 | XP_005651896.1 | superoxide dismutase [Mn], mitochondrial [Coccomyxa subellipsoideaC-169] | 1.00E-90 | csl:COCSUDRAFT_52134 | K04564 |
| TRINITY1_DN22763_c3_g1_i1 | 0.00E+00 | 4.00E-01 | XP_002955293.1 | hypothetical protein VOLCADRAFT_76662 [Volvox carteri f. nagariensis] | 1.00E-156 | vcn:VOLCADRAFT_76662 | K05298 |
| TRINITY2_DN24624_c0_g4_i1 | 0.00E+00 | 3.10E-01 | XP_002946369.1 | sterol 14 desaturase [Volvox carteri f. nagariensis] | 0.00E+00 | vcn:VOLCADRAFT_72667 | K05917 |
| TRINITY1_DN3625_c0_g1_i1 | 0.00E+00 | 1.31E-01 | XP_001697898.1 | UDP-sulfoquinovose synthase [Chlamydomonas reinhardtii] | 0.00E+00 | cre:CHLREDRAFT_27658 | K06118 |
| Locus_829_6Transcript_2/9_Confidence_0.609_Length_1544 | 0.00E+00 | 1.81E-01 | XP_005644605.1 | UDP-Glycosyltransferase/glycogen phosphorylase [Coccomyxasubellipsoidea C-169] | 1.00E-124 | csl:COCSUDRAFT_18860 | K06119 |
| TRINITY1_DN48099_c0_g1_i1 | 0.00E+00 | 2.93E-01 | XP_002945935.1 | hypothetical protein VOLCADRAFT_54924 [Volvox carteri f. nagariensis] | 1.00E-165 | vcn:VOLCADRAFT_54924 | K06444 |
| TRINITY2_DN67118_c0_g1_i1 | 0.00E+00 | 2.21E-01 | XP_002953146.1 | hypothetical protein VOLCADRAFT_63274 [Volvox carteri f. nagariensis] | 1.00E-117 | vcn:VOLCADRAFT_63274 | K07071 |
| comp31172_c2_seq4 | 6.57E-04 | 4.38E-01 | XP_001690442.1 | oligsaccharyl transferase STT3 subunit [Chlamydomonas reinhardtii] | 0.00E+00 | cre:CHLREDRAFT_127991 | K07151 |
| Locus_11593_9Transcript_1/1_Confidence_1.000_Length_580 | 1.91E-05 | 3.64E-01 | XP_001699051.1 | hypothetical protein CHLREDRAFT_106339 [Chlamydomonas reinhardtii] | 1.00E-52 | cre:CHLREDRAFT_106339 | K07305 |
| TRINITY2_DN26828_c2_g1_i1 | 1.50E-04 | 4.98E-01 | XP_001701644.1 | cGMP-dependent protein kinase [Chlamydomonas reinhardtii] | 0.00E+00 | cre:CHLREDRAFT_181974 | K07376 |
| Locus_443_5Transcript_1/5_Confidence_0.333_Length_1015 | 0.00E+00 | 4.44E-02 | XP_002947155.1 | oxygen-evolving enhancer protein 3 [Volvox carteri f. nagariensis] | 5.00E-28 | vcn:VOLCADRAFT_79577 | K08901 |
| Locus_103_7Transcript_2/2_Confidence_0.750_Length_948 | 0.00E+00 | 1.33E-01 | XP_002956213.1 | hypothetical protein VOLCADRAFT_107104 [Volvox carteri f.nagariensis] | 1.00E-40 | vcn:VOLCADRAFT_107104 | K08902 |
| Locus_37_4Transcript_1/1_Confidence_1.000_Length_664 | 0.00E+00 | 2.78E-01 | XP_001703126.1 | photosystem I reaction center subunit V, partial [Chlamydomonasreinhardtii] | 6.00E-20 | cre:CHLREDRAFT_165416 | K08905 |
| Locus_2017_9Transcript_1/1_Confidence_1.000_Length_1200 | 0.00E+00 | 8.45E-02 | XP_002958611.1 | light-harvesting protein of photosystem I [Volvox carteri f.nagariensis] | 2.00E-62 | vcn:VOLCADRAFT_77985 | K08907 |
| TRINITY2_DN85890_c0_g1_i1 | 0.00E+00 | 1.65E-01 | XP_001778557.1 | predicted protein [Physcomitrella patens] | 9.00E-12 | ppp:PHYPADRAFT_222728 | K08908 |
| TRINITY1_DN47544_c0_g2_i1 | 0.00E+00 | 2.06E-01 | XP_001701405.1 | light-harvesting chlorophyll-a/b protein of photosystem I, type III[Chlamydomonas reinhardtii] | 8.00E-88 | cre:CHLREDRAFT_153678 | K08909 |
| TRINITY1_DN46084_c0_g1_i6 | 0.00E+00 | 2.04E-01 | XP_002950368.1 | light harvesting complex a protein [Volvox carteri f. nagariensis] | 5.00E-80 | vcn:VOLCADRAFT_104631 | K08911 |
| TRINITY1_DN37534_c39_g1_i7 | 0.00E+00 | 6.01E-02 | XP_001695466.1 | chlorophyll a-b binding protein of LHCII [Chlamydomonasreinhardtii] | 1.00E-108 | cre:CHLREDRAFT_184479 | K08912 |
| TRINITY2_DN24849_c20_g1_i1 | 0.00E+00 | 2.14E-02 | XP_002952704.1 | light-harvesting chlorophyll a/b-binding protein [Volvox carteri f.nagariensis] | 1.00E-106 | vcn:VOLCADRAFT_109843 | K08913 |
| TRINITY2_DN14665_c0_g2_i1 | 0.00E+00 | 5.03E-02 | XP_001697193.1 | chlorophyll a-b binding protein of photosystem II [Chlamydomonasreinhardtii] | 5.00E-94 | cre:CHLREDRAFT_184810 | K08915 |
| Locus_1504_7Transcript_1/3_Confidence_0.727_Length_1210 | 0.00E+00 | 9.53E-02 | XP_001695927.1 | minor chlorophyll a-b binding protein of photosystem II[Chlamydomonas reinhardtii] | 1.00E-114 | cre:CHLREDRAFT_184397 | K08916 |
| TRINITY1_DN55273_c1_g1_i1 | 0.00E+00 | 3.00E-01 | XP_002956811.1 | hypothetical protein VOLCADRAFT_83790 [Volvox carteri f. nagariensis] | 1.00E-179 | vcn:VOLCADRAFT_83790 | K09458 |
| Locus_9589_8Transcript_1/1_Confidence_1.000_Length_648 | 5.77E-10 | 1.71E-01 | XP_001693633.1 | peptidyl-prolyl cis-trans isomerase, cyclophilin-type[Chlamydomonas reinhardtii] | 2.00E-64 | cre:CHLREDRAFT_30639 | K09565 |
| comp68086_c0_seq1 | 2.46E-04 | 2.53E-01 | XP_001692930.1 | predicted protein [Chlamydomonas reinhardtii] | 1.00E-53 | cre:CHLREDRAFT_205936 | K09775 |
| TRINITY1_DN52835_c0_g1_i2 | 0.00E+00 | 4.62E-01 | XP_001689497.1 | sugar nucleotide epimerase [Chlamydomonas reinhardtii] | 1.00E-178 | cre:CHLREDRAFT_196952 | K10046 |
| TRINITY2_DN26451_c3_g4_i10 | 0.00E+00 | 1.06E-01 | XP_001696697.1 | nitrate reductase [Chlamydomonas reinhardtii] | 0.00E+00 | cre:CHLREDRAFT_184661 | K10534 |
| Locus_7510_7Transcript_2/2_Confidence_1.000_Length_3076 | 4.57E-07 | 4.76E-01 | XP_001701820.1 | spliceosome component, nuclear pre-mRNA splicing factor[Chlamydomonas reinhardtii] | 1.00E-169 | cre:CHLREDRAFT_196941 | K10599 |
| TRINITY2_DN3960_c0_g1_i1 | 3.33E-16 | 2.94E-01 | XP_001697709.1 | hypothetical protein CHLREDRAFT_131472 [Chlamydomonas reinhardtii] | 2.00E-69 | cre:CHLREDRAFT_131472 | K10689 |
| TRINITY1_DN60674_c0_g1_i1 | 3.51E-07 | 2.22E-01 | XP_005650924.1 | endoplasmic reticulum oxidoreductin 1 [Coccomyxa subellipsoideaC-169] | 1.00E-114 | csl:COCSUDRAFT_58917 | K10950 |
| TRINITY2_DN46977_c1_g1_i1 | 0.00E+00 | 6.41E-02 | XP_001690248.1 | geranylgeranyl reductase [Chlamydomonas reinhardtii] | 0.00E+00 | cre:CHLREDRAFT_136810 | K10960 |
| Locus_1775_3Transcript_11/13_Confidence_0.589_Length_5694 | 1.36E-05 | 3.45E-01 | XP_002947645.1 | hypothetical protein VOLCADRAFT_120505 [Volvox carteri f.nagariensis] | 1.00E-121 | vcn:VOLCADRAFT_120505 | K11407 |
| TRINITY2_DN19893_c0_g2_i1 | 1.49E-11 | 1.88E-01 | XP_002948887.1 | hypothetical protein VOLCADRAFT_104081 [Volvox carteri f.nagariensis] | 1.00E-119 | vcn:VOLCADRAFT_104081 | K12164 |
| Locus_11_3Transcript_5254/11887_Confidence_1.000_Length_1243 | 0.00E+00 | 9.42E-02 | XP_002951989.1 | hypothetical protein VOLCADRAFT_105313 [Volvox carteri f.nagariensis] | 9.00E-89 | vcn:VOLCADRAFT_105313 | K12271 |
| TRINITY1_DN57731_c0_g1_i1 | 0.00E+00 | 1.30E-01 | XP_002947173.1 | hypothetical protein VOLCADRAFT_56724, partial [Volvox carteri f.nagariensis] | 1.00E-138 | vcn:VOLCADRAFT_56724 | K12347 |
| TRINITY1_DN8802_c1_g1_i1 | 0.00E+00 | 6.45E-02 | XP_002956365.1 | oxygen-evolving enhancer protein 2 [Volvox carteri f. nagariensis] | 4.00E-77 | vcn:VOLCADRAFT_121519 | K12385 |
| Locus_1652_2Transcript_2/4_Confidence_0.636_Length_1920 | 1.77E-02 | 4.86E-01 | XP_001699178.1 | nuclear pre-mRNA splicing factor and U1 snRNP component, partial[Chlamydomonas reinhardtii] | 1.00E-125 | cre:CHLREDRAFT_193637 | K13217 |
| TRINITY2_DN17070_c0_g2_i1 | 0.00E+00 | 2.55E-01 | XP_001690175.1 | chlorophyll a oxygenase [Chlamydomonas reinhardtii] | 1.00E-177 | cre:CHLREDRAFT_195951 | K13600 |
| TRINITY1_DN3211_c2_g1_i1 | 0.00E+00 | 2.86E-01 | XP_002955110.1 | hypothetical protein VOLCADRAFT_83033 [Volvox carteri f. nagariensis] | 0.00E+00 | vcn:VOLCADRAFT_83033 | K13621 |
| comp30019_c0_seq20 | 0.00E+00 | 2.07E-01 | XP_001698518.1 | alanine aminotransferase [Chlamydomonas reinhardtii] | 0.00E+00 | cre:CHLREDRAFT_206184 | K14272 |
| Locus_9867_7Transcript_1/1_Confidence_1.000_Length_670 | 0.00E+00 | 1.76E-01 | XP_002950988.1 | photosystem I reaction center subunit O [Volvox carteri f.nagariensis] | 6.00E-37 | vcn:VOLCADRAFT_120934 | K14332 |
| Locus_20932_10Transcript_1/1_Confidence_1.000_Length_709 | 1.20E-04 | 2.98E-01 | XP_002958777.1 | hypothetical protein VOLCADRAFT_69909 [Volvox carteri f.nagariensis] | 8.00E-30 | vcn:VOLCADRAFT_69909 | K14490 |
| Locus_201_6Transcript_27/27_Confidence_0.133_Length_3130 | 0.00E+00 | 2.92E-01 | XP_001694873.1 | sodium/phosphate symporter, partial [Chlamydomonas reinhardtii] | 1.00E-118 | cre:CHLREDRAFT_196438 | K14640 |
| TRINITY1_DN48035_c0_g1_i1 | 0.00E+00 | 1.89E-03 | XP_001694585.1 | multicopper ferroxidase [Chlamydomonas reinhardtii] | 0.00E+00 | cre:CHLREDRAFT_184156 | K14735 |
| TRINITY1_DN57611_c0_g1_i1 | 4.36E-06 | 2.32E-01 | XP_001691328.1 | mitochondrial carrier protein [Chlamydomonas reinhardtii] | 1.00E-126 | cre:CHLREDRAFT_188977 | K15109 |
| TRINITY1_DN2597_c0_g1_i1 | 1.31E-10 | 2.82E-01 | XP_002958826.1 | hypothetical protein VOLCADRAFT_100143 [Volvox carteri f.nagariensis] | 0.00E+00 | vcn:VOLCADRAFT_100143 | K15747 |
| TRINITY1_DN55230_c0_g1_i1 | 2.44E-04 | 4.77E-01 | XP_002949292.1 | hypothetical protein VOLCADRAFT_89593 [Volvox carteri f. nagariensis] | 1.00E-159 | vcn:VOLCADRAFT_89593 | K15893 |
| TRINITY1_DN55486_c0_g1_i1 | 2.39E-07 | 4.27E-01 | XP_002953616.1 | hypothetical protein VOLCADRAFT_94435 [Volvox carteri f. nagariensis] | 1.00E-112 | vcn:VOLCADRAFT_94435 | K15918 |
| Locus_7091_8Transcript_1/9_Confidence_0.104_Length_3735 | 3.09E-06 | 4.63E-01 | XP_002950146.1 | hypothetical protein VOLCADRAFT_60154 [Volvox carteri f. nagariensis] | 1.00E-142 | vcn:VOLCADRAFT_60154 | K16276 |
| TRINITY1_DN36296_c5_g1_i11 | 2.26E-02 | 2.21E-01 | XP_001693368.1 | hypothetical protein CHLREDRAFT_190601 [Chlamydomonas reinhardtii] | 3.00E-59 | cre:CHLREDRAFT_190601 | K17525 |
| Locus_1080_5Transcript_1/2_Confidence_0.667_Length_1039 | 7.38E-07 | 4.09E-01 | XP_001701911.1 | plastid-specific ribosomal protein 3 [Chlamydomonas reinhardtii] | 8.00E-50 | cre:CHLREDRAFT_195633 | K19032 |
| Locus_5195_5Transcript_1/1_Confidence_1.000_Length_1392 | 1.43E-02 | 1.47E-01 | XP_002953959.1 | hypothetical protein VOLCADRAFT_76013 [Volvox carteri f. nagariensis] | 1.00E-142 | vcn:VOLCADRAFT_76013 | K19073 |
| Locus_1040_4Transcript_1/1_Confidence_1.000_Length_888 | 0.00E+00 | 2.79E-04 | XP_005647526.1 | flavodoxin IsiB [Coccomyxa subellipsoidea C-169] | 7.00E-52 | csl:COCSUDRAFT_53474 |  |
| TRINITY2_DN41486_c0_g1_i1 | 0.00E+00 | 1.82E-03 | XP_007141246.1 | hypothetical protein PHAVU_008G179700g [Phaseolus vulgaris] | 6.00E-19 | pvu:PHAVU_008G179700g |  |
| TRINITY2_DN20149_c28_g4_i1 | 0.00E+00 | 2.29E-03 | XP_012857134.1 | PREDICTED: uncharacterized protein LOC105976409 [Erythrantheguttata] | 1.00E-42 |  |  |
| TRINITY1_DN41800_c6_g1_i1 | 0.00E+00 | 4.88E-03 | WP_015230410.1 | extracellular nuclease [Dactylococcopsis salina] | 1.00E-101 |  |  |
| Locus_316_3Transcript_1/6_Confidence_0.250_Length_794 | 0.00E+00 | 6.48E-03 | XP_002945683.1 | hypothetical protein VOLCADRAFT_102653 [Volvox carteri f.nagariensis] | 1.00E-17 | vcn:VOLCADRAFT_102653 |  |
| Locus_2406_2Transcript_5/9_Confidence_0.522_Length_1918 | 0.00E+00 | 7.44E-03 | XP_001702318.1 | alkaline phosphatase [Chlamydomonas reinhardtii] | 1.00E-98 | cre:CHLREDRAFT_196484 |  |
| TRINITY1_DN41800_c6_g1_i3 | 0.00E+00 | 8.59E-03 | WP_014201896.1 | hypothetical protein [Owenweeksia hongkongensis] | 4.00E-26 |  |  |
| TRINITY1_DN40017_c0_g1_i1 | 1.45E-10 | 1.47E-02 | XP_001694065.1 | predicted protein [Chlamydomonas reinhardtii] | 6.00E-11 | cre:CHLREDRAFT_173173 |  |
| TRINITY1_DN48116_c0_g1_i1 | 0.00E+00 | 1.84E-02 | XP_002948144.1 | hypothetical protein VOLCADRAFT_88469 [Volvox carteri f.nagariensis] | 1.00E-21 | vcn:VOLCADRAFT_88469 |  |
| TRINITY1_DN47868_c14_g2_i7 | 8.15E-12 | 2.50E-02 | XP_005846992.1 | hypothetical protein CHLNCDRAFT_135008 [Chlorella variabilis] | 5.00E-25 | cvr:CHLNCDRAFT_135008 |  |
| Locus_11_3Transcript_3466/11887_Confidence_1.000_Length_1067 | 2.10E-02 | 2.52E-02 | XP_001703670.1 | predicted protein, partial [Chlamydomonas reinhardtii] | 2.00E-17 | cre:CHLREDRAFT_154732 |  |
| TRINITY1_DN14170_c1_g2_i1 | 0.00E+00 | 2.66E-02 | XP_013901948.1 | protein kinase A [Monoraphidium neglectum] | 0.00E+00 |  |  |
| Locus_1_6Transcript_563/2655_Confidence_1.000_Length_1409 | 3.58E-10 | 2.82E-02 | XP_001692802.1 | predicted protein [Chlamydomonas reinhardtii] | 3.00E-15 | cre:CHLREDRAFT_147777 |  |
| comp22669_c0_seq1 | 9.73E-08 | 2.87E-02 | XP_002954272.1 | hypothetical protein VOLCADRAFT_95012 [Volvox carteri f.nagariensis] | 2.00E-51 | vcn:VOLCADRAFT_95012 |  |
| TRINITY2_DN18003_c0_g2_i1 | 0.00E+00 | 3.28E-02 | XP_002946660.1 | hypothetical protein VOLCADRAFT_103076 [Volvox carteri f.nagariensis] | 2.00E-30 | vcn:VOLCADRAFT_103076 |  |
| Locus_15047_7Transcript_1/2_Confidence_0.400_Length_1725 | 0.00E+00 | 3.30E-02 | XP_002958870.1 | low molecular mass early light-induced protein [Volvox carteri f.nagariensis] | 4.00E-79 | vcn:VOLCADRAFT_100204 |  |
| comp29257_c6_seq1 | 1.23E-10 | 3.36E-02 | XP_013895813.1 | hypothetical protein MNEG_11167, partial [Monoraphidium neglectum] | 3.00E-78 |  |  |
| TRINITY1_DN41450_c1_g2_i1 | 3.10E-12 | 3.59E-02 | XP_001695893.1 | gamete-specific protein, partial [Chlamydomonas reinhardtii] | 1.00E-167 | cre:CHLREDRAFT_205626 |  |
| Locus_21493_10Transcript_1/2_Confidence_0.667_Length_524 | 2.66E-15 | 3.88E-02 | XP_013893390.1 | hypothetical protein MNEG_13591 [Monoraphidium neglectum] | 6.00E-11 |  |  |
| TRINITY1_DN46062_c19_g6_i1 | 0.00E+00 | 3.98E-02 | XP_001689712.1 | predicted protein [Chlamydomonas reinhardtii] | 1.00E-101 | cre:CHLREDRAFT_190061 |  |
| TRINITY2_DN18251_c0_g1_i3 | 6.06E-05 | 4.09E-02 | XP_010519276.1 | PREDICTED: protein LHCP TRANSLOCATION DEFECT [Tarenaya hassleriana] | 6.00E-14 | thj:104798778 |  |
| TRINITY1_DN101_c0_g1_i1 | 1.23E-04 | 4.13E-02 | XP_001703446.1 | hypothetical protein CHLREDRAFT_168953 [Chlamydomonas reinhardtii] | 5.00E-22 | cre:CHLREDRAFT_168953 |  |
| Locus_2144_6Transcript_1/1_Confidence_1.000_Length_1227 | 0.00E+00 | 4.17E-02 | XP_001690406.1 | hypothetical protein CHLREDRAFT_182934 [Chlamydomonas reinhardtii] | 6.00E-95 | cre:CHLREDRAFT_182934 |  |
| TRINITY2_DN23390_c0_g4_i1 | 1.17E-08 | 4.17E-02 | XP_002948810.1 | hypothetical protein VOLCADRAFT_89065 [Volvox carteri f.nagariensis] | 6.00E-27 | vcn:VOLCADRAFT_89065 |  |
| TRINITY2_DN19990_c0_g2_i1 | 0.00E+00 | 4.66E-02 | XP_009143435.1 | PREDICTED: uncharacterized protein LOC103867148 [Brassica rapa] | 3.00E-18 | brp:103867148 |  |
| TRINITY1_DN55665_c2_g1_i1 | 7.31E-06 | 5.08E-02 | XP_013893279.1 | chlorophyll a/b-binding apoprotein CP26 precursor [Monoraphidiumneglectum] | 2.00E-11 |  |  |
| Locus_5306_7Transcript_3/5_Confidence_0.704_Length_718 | 0.00E+00 | 5.33E-02 | XP_013906230.1 | Chlorophyll a-b binding protein [Monoraphidium neglectum] | 3.00E-90 |  |  |
| Locus_522_4Transcript_1/1_Confidence_1.000_Length_1159 | 0.00E+00 | 5.38E-02 | XP_005851689.1 | hypothetical protein CHLNCDRAFT_132964 [Chlorella variabilis] | 2.00E-16 | cvr:CHLNCDRAFT_132964 |  |
| TRINITY1_DN488_c1_g1_i1 | 0.00E+00 | 5.76E-02 | XP_001702242.1 | maturation/stability factor for petA mRNA [Chlamydomonas reinhardtii] | 1.00E-128 | cre:CHLREDRAFT_205934 |  |
| TRINITY1_DN45190_c0_g1_i5 | 8.56E-10 | 5.99E-02 | XP_001699421.1 | low molecular mass early light-induced protein [Chlamydomonasreinhardtii] | 4.00E-31 | cre:CHLREDRAFT_185309 |  |
| TRINITY2_DN45698_c0_g1_i1 | 3.14E-14 | 6.03E-02 | XP_013904101.1 | Chlorophyll a-b binding protein [Monoraphidium neglectum] | 6.00E-11 |  |  |
| comp74676_c0_seq1 | 0.00E+00 | 6.07E-02 | XP_005846126.1 | hypothetical protein CHLNCDRAFT_59722 [Chlorella variabilis] | 2.00E-32 | cvr:CHLNCDRAFT_59722 |  |
| TRINITY1_DN503_c6_g1_i2 | 0.00E+00 | 6.18E-02 | XP_013902800.1 | Chlorophyll a-b binding protein of LHCII typeI [Monoraphidiumneglectum] | 2.00E-20 |  |  |
| TRINITY1_DN30708_c0_g1_i1 | 3.81E-07 | 6.67E-02 | XP_002949129.1 | hypothetical protein VOLCADRAFT_89530 [Volvox carteri f. nagariensis] | 1.00E-90 | vcn:VOLCADRAFT_89530 |  |
| Locus_992_4Transcript_1/1_Confidence_1.000_Length_1249 | 0.00E+00 | 6.84E-02 | XP_005844290.1 | hypothetical protein CHLNCDRAFT_37063 [Chlorella variabilis] | 1.00E-134 | cvr:CHLNCDRAFT_37063 |  |
| comp18318_c0_seq2 | 0.00E+00 | 6.94E-02 | XP_002958433.1 | hypothetical protein VOLCADRAFT_99686 [Volvox carteri f. nagariensis] | 2.00E-33 | vcn:VOLCADRAFT_99686 |  |
| Locus_30418_5Transcript_1/1_Confidence_1.000_Length_1090 | 3.63E-07 | 7.41E-02 | XP_001699230.1 | hypothetical protein CHLREDRAFT_121355, partial [Chlamydomonasreinhardtii] | 3.00E-80 | cre:CHLREDRAFT_121355 |  |
| Locus_2122_6Transcript_1/1_Confidence_1.000_Length_997 | 0.00E+00 | 7.51E-02 | XP_005849085.1 | hypothetical protein CHLNCDRAFT_143586 [Chlorella variabilis] | 9.00E-64 | cvr:CHLNCDRAFT_143586 |  |
| Locus_286_4Transcript_1/1_Confidence_1.000_Length_808 | 2.01E-07 | 7.58E-02 | XP_013893154.1 | thylakoid lumenal protein [Monoraphidium neglectum] | 3.00E-28 |  |  |
| TRINITY1_DN27045_c0_g1_i1 | 2.00E-15 | 7.61E-02 | XP_001691232.1 | magnesium chelatase subunit I [Chlamydomonas reinhardtii] | 5.00E-68 | cre:CHLREDRAFT_135762 |  |
| comp39170_c0_seq1 | 0.00E+00 | 7.63E-02 | XP_005651506.1 | cobalamin-independent methionine synthase [Coccomyxa subellipsoideaC-169] | 0.00E+00 | csl:COCSUDRAFT_21995 |  |
| TRINITY1_DN58057_c0_g2_i1 | 2.44E-15 | 7.91E-02 | XP_001693319.1 | predicted protein [Chlamydomonas reinhardtii] | 4.00E-36 | cre:CHLREDRAFT_183917 |  |
| Locus_3089_8Transcript_1/3_Confidence_0.667_Length_1269 | 0.00E+00 | 8.02E-02 | XP_002945953.1 | hypothetical protein VOLCADRAFT_78986 [Volvox carteri f. nagariensis] | 6.00E-85 | vcn:VOLCADRAFT_78986 |  |
| Locus_3892_2Transcript_1/2_Confidence_0.667_Length_987 | 0.00E+00 | 8.45E-02 | XP_002946304.1 | nucleic acid binding protein [Volvox carteri f. nagariensis] | 2.00E-35 | vcn:VOLCADRAFT_78979 |  |
| TRINITY1_DN56194_c2_g1_i1 | 2.03E-09 | 8.57E-02 | XP_015689202.1 | PREDICTED: chlorophyll a-b binding protein 2, chloroplastic-like[Oryza brachyantha] | 2.00E-11 |  |  |
| TRINITY1_DN2196_c1_g2_i1 | 0.00E+00 | 8.77E-02 | XP_002946663.1 | hypothetical protein VOLCADRAFT_103079 [Volvox carteri f.nagariensis] | 7.00E-84 | vcn:VOLCADRAFT_103079 |  |
| Locus_100_5Transcript_43/88_Confidence_1.000_Length_840 | 3.98E-09 | 9.64E-02 | XP_002949186.1 | hypothetical protein VOLCADRAFT_120742 [Volvox carteri f.nagariensis] | 1.00E-66 | vcn:VOLCADRAFT_120742 |  |
| Locus_8390_7Transcript_1/1_Confidence_1.000_Length_1177 | 0.00E+00 | 9.88E-02 | XP_002953569.1 | hypothetical protein VOLCADRAFT_106005 [Volvox carteri f.nagariensis] | 1.00E-52 | vcn:VOLCADRAFT_106005 |  |
| TRINITY1_DN57677_c0_g2_i1 | 0.00E+00 | 9.92E-02 | XP_002950151.1 | hypothetical protein VOLCADRAFT_90465 [Volvox carteri f. nagariensis] | 1.00E-119 | vcn:VOLCADRAFT_90465 |  |
| TRINITY1_DN45373_c12_g2_i2 | 1.06E-10 | 1.01E-01 | XP_005643283.1 | methionine adenosyltransferase regulatory beta subunit-related[Coccomyxa subellipsoidea C-169] | 2.00E-68 | csl:COCSUDRAFT_20470 |  |
| Locus_3217_6Transcript_3/3_Confidence_0.600_Length_1300 | 1.36E-06 | 1.02E-01 | XP_002949336.1 | hypothetical protein VOLCADRAFT_104273 [Volvox carteri f.nagariensis] | 3.00E-89 | vcn:VOLCADRAFT_104273 |  |
| TRINITY1_DN57717_c0_g1_i1 | 0.00E+00 | 1.03E-01 | WP_009150117.1 | dimethylglycine methyltransferase [Thiorhodovibrio sp. 970] | 1.00E-45 |  |  |
| Locus_7752_2Transcript_3/3_Confidence_0.750_Length_1349 | 0.00E+00 | 1.06E-01 | XP_002951983.1 | ferric-chelate reductase [Volvox carteri f. nagariensis] | 3.00E-34 | vcn:VOLCADRAFT_105306 |  |
| TRINITY1_DN16695_c0_g1_i1 | 0.00E+00 | 1.11E-01 | XP_001703004.1 | predicted protein [Chlamydomonas reinhardtii] | 0.00E+00 | cre:CHLREDRAFT_140509 |  |
| Locus_5983_9Transcript_1/1_Confidence_1.000_Length_723 | 1.71E-10 | 1.13E-01 | XP_001702112.1 | membrane protein [Chlamydomonas reinhardtii] | 1.00E-61 | cre:CHLREDRAFT_206091 |  |
| TRINITY2_DN22547_c0_g2_i2 | 2.31E-02 | 1.16E-01 | XP_001694561.1 | hydroxyproline-rich glycoprotein [Chlamydomonas reinhardtii] | 1.00E-17 | cre:CHLREDRAFT_148231 |  |
| TRINITY1_DN50551_c0_g1_i1 | 0.00E+00 | 1.21E-01 | XP_001692034.1 | peptidyl-prolyl cis-trans isomerase, cyclophilin-type [Chlamydomonasreinhardtii] | 1.00E-125 | cre:CHLREDRAFT_196558 |  |
| Locus_23514_10Transcript_1/1_Confidence_1.000_Length_1374 | 0.00E+00 | 1.24E-01 | XP_001702364.1 | rhodanese-like Ca-sensing receptor [Chlamydomonas reinhardtii] | 9.00E-70 | cre:CHLREDRAFT_194676 |  |
| TRINITY2_DN18713_c0_g2_i1 | 2.33E-15 | 1.25E-01 | XP_013897006.1 | hypothetical protein MNEG_9976 [Monoraphidium neglectum] | 2.00E-25 |  |  |
| comp27249_c0_seq3 | 0.00E+00 | 1.27E-01 | XP_002949172.1 | hypothetical protein VOLCADRAFT_104245 [Volvox carteri f.nagariensis] | 1.00E-68 | vcn:VOLCADRAFT_104245 |  |
| TRINITY2_DN37300_c0_g1_i1 | 4.48E-10 | 1.30E-01 | XP_005843199.1 | expressed protein [Chlorella variabilis] | 2.00E-83 | cvr:CHLNCDRAFT_59328 |  |
| TRINITY1_DN60459_c0_g1_i1 | 0.00E+00 | 1.31E-01 | XP_002945687.1 | hypothetical protein VOLCADRAFT_78664 [Volvox carteri f. nagariensis] | 1.00E-144 | vcn:VOLCADRAFT_78664 |  |
| TRINITY2_DN26027_c0_g3_i3 | 2.20E-05 | 1.36E-01 | XP_001696632.1 | minus agglutinin protein [Chlamydomonas reinhardtii] | 4.00E-20 | cre:CHLREDRAFT_142415 |  |
| TRINITY1_DN46190_c0_g3_i1 | 0.00E+00 | 1.37E-01 | XP_013901241.1 | Chlorophyll a-b binding protein 7, chloroplastic [Monoraphidiumneglectum] | 7.00E-49 |  |  |
| TRINITY1_DN47371_c2_g3_i1 | 0.00E+00 | 1.38E-01 | XP_001689713.1 | predicted protein [Chlamydomonas reinhardtii] | 0.00E+00 | cre:CHLREDRAFT_190062 |  |
| TRINITY1_DN19033_c1_g1_i1 | 0.00E+00 | 1.38E-01 | XP_008775505.1 | PREDICTED: protein LHCP TRANSLOCATION DEFECT [Phoenix dactylifera] | 4.00E-17 | pda:103695857 |  |
| TRINITY2_DN26204_c2_g2_i2 | 1.29E-13 | 1.38E-01 | XP_013899889.1 | hypothetical protein MNEG_7092 [Monoraphidium neglectum] | 2.00E-15 |  |  |
| Locus_5048_10Transcript_1/1_Confidence_1.000_Length_887 | 0.00E+00 | 1.40E-01 | XP_001695823.1 | flagellar associated protein [Chlamydomonas reinhardtii] | 1.00E-57 | cre:CHLREDRAFT_149145 |  |
| Locus_4252_6Transcript_1/1_Confidence_1.000_Length_1281 | 0.00E+00 | 1.41E-01 | XP_005645620.1 | S-adenosyl-L-methionine-dependent methyltransferase [Coccomyxasubellipsoidea C-169] | 8.00E-91 | csl:COCSUDRAFT_48266 |  |
| comp42503_c1_seq1 | 1.46E-09 | 1.45E-01 | XP_002948962.1 | hypothetical protein VOLCADRAFT_89362 [Volvox carteri f.nagariensis] | 3.00E-32 | vcn:VOLCADRAFT_89362 |  |
| Locus_1444_3Transcript_7/9_Confidence_0.636_Length_3193 | 3.78E-02 | 1.45E-01 | WP_005458189.1 | SAM-dependent methyltransferase [Saccharomonospora cyanea] | 4.00E-20 |  |  |
| TRINITY2_DN24883_c0_g2_i1 | 7.50E-05 | 1.46E-01 | XP_005845786.1 | hypothetical protein CHLNCDRAFT_25506, partial [Chlorella variabilis] | 1.00E-137 | cvr:CHLNCDRAFT_25506 |  |
| Locus_10863_5Transcript_2/4_Confidence_0.727_Length_962 | 8.00E-07 | 1.46E-01 | XP_001691033.1 | glutathione S-transferase [Chlamydomonas reinhardtii] | 1.00E-75 | cre:CHLREDRAFT_188638 |  |
| Locus_18582_2Transcript_1/1_Confidence_1.000_Length_1283 | 1.28E-12 | 1.47E-01 | XP_005647503.1 | hypothetical protein COCSUDRAFT_15824 [Coccomyxa subellipsoideaC-169] | 1.00E-77 | csl:COCSUDRAFT_15824 |  |
| TRINITY1_DN45999_c0_g2_i2 | 3.44E-15 | 1.47E-01 | XP_001692244.1 | rubisco activase [Chlamydomonas reinhardtii] | 1.00E-50 | cre:CHLREDRAFT_128745 |  |
| TRINITY1_DN60358_c0_g1_i1 | 2.35E-05 | 1.49E-01 | XP_001702554.1 | cytochrome P450, CYP85 clan [Chlamydomonas reinhardtii] | 2.00E-44 | cre:CHLREDRAFT_196683 |  |
| Locus_7240_2Transcript_2/2_Confidence_0.750_Length_1453 | 4.60E-11 | 1.49E-01 | XP_001701876.1 | predicted protein [Chlamydomonas reinhardtii] | 1.00E-44 | cre:CHLREDRAFT_186500 |  |
| Locus_655_3Transcript_1/1_Confidence_1.000_Length_1015 | 0.00E+00 | 1.55E-01 | XP_002949125.1 | hypothetical protein VOLCADRAFT_80525 [Volvox carteri f.nagariensis] | 3.00E-72 | vcn:VOLCADRAFT_80525 |  |
| Locus_2848_4Transcript_1/1_Confidence_1.000_Length_796 | 0.00E+00 | 1.64E-01 | XP_002952356.1 | hypothetical protein VOLCADRAFT_75357 [Volvox carteri f.nagariensis] | 1.00E-68 | vcn:VOLCADRAFT_75357 |  |
| TRINITY2_DN14778_c0_g1_i1 | 2.15E-08 | 1.68E-01 | XP_002948554.1 | uroporphyrinogen decarboxylase chloroplast precursor [Volvox carterif. nagariensis] | 1.00E-144 | vcn:VOLCADRAFT_103889 |  |
| TRINITY2_DN26792_c1_g2_i1 | 0.00E+00 | 1.68E-01 | XP_013903192.1 | hypothetical protein MNEG_3777 [Monoraphidium neglectum] | 1.00E-73 |  |  |
| TRINITY2_DN37619_c0_g1_i1 | 3.75E-09 | 1.69E-01 | XP_002954801.1 | hypothetical protein VOLCADRAFT_76453 [Volvox carteri f.nagariensis] | 6.00E-34 | vcn:VOLCADRAFT_76453 |  |
| Locus_5186_2Transcript_1/1_Confidence_1.000_Length_1000 | 0.00E+00 | 1.71E-01 | XP_002946407.1 | hypothetical protein VOLCADRAFT_55820, partial [Volvox carteri f.nagariensis] | 2.00E-46 | vcn:VOLCADRAFT_55820 |  |
| TRINITY2_DN57452_c0_g1_i1 | 2.30E-09 | 1.74E-01 | XP_001693388.1 | thylakoid lumen protein [Chlamydomonas reinhardtii] | 4.00E-49 | cre:CHLREDRAFT_205649 |  |
| Locus_7226_2Transcript_1/1_Confidence_1.000_Length_1410 | 5.17E-12 | 1.75E-01 | WP_015214616.1 | PEP motif anchor domain protein [Anabaena cylindrica] | 3.00E-16 |  |  |
| Locus_9294_7Transcript_1/1_Confidence_1.000_Length_1119 | 2.43E-06 | 1.75E-01 | XP_001702908.1 | nucleoside diphosphate sugar epimerase [Chlamydomonas reinhardtii] | 5.00E-59 | cre:CHLREDRAFT_205577 |  |
| TRINITY1_DN8961_c0_g1_i1 | 7.60E-10 | 1.77E-01 | XP_002946577.1 | chloroplast ribosome-associated protein [Volvox carteri f.nagariensis] | 1.00E-132 | vcn:VOLCADRAFT_103003 |  |
| TRINITY2_DN7895_c0_g1_i1 | 2.81E-06 | 1.79E-01 | XP_005843757.1 | hypothetical protein CHLNCDRAFT_139849 [Chlorella variabilis] | 3.00E-34 | cvr:CHLNCDRAFT_139849 |  |
| TRINITY2_DN92_c0_g1_i1 | 0.00E+00 | 1.80E-01 | XP_013905851.1 | hypothetical protein MNEG_1119 [Monoraphidium neglectum] | 9.00E-83 |  |  |
| comp45308_c0_seq1 | 0.00E+00 | 1.80E-01 | XP_001701444.1 | predicted protein [Chlamydomonas reinhardtii] | 4.00E-78 | cre:CHLREDRAFT_194448 |  |
| Locus_1337_4Transcript_1/1_Confidence_1.000_Length_867 | 3.03E-11 | 1.80E-01 | XP_001696792.1 | rieske [2Fe-2S] protein [Chlamydomonas reinhardtii] | 8.00E-71 | cre:CHLREDRAFT_192099 |  |
| Locus_6215_6Transcript_1/1_Confidence_1.000_Length_1164 | 2.38E-04 | 1.83E-01 | XP_001691411.1 | NADH nitrate reductase [Chlamydomonas reinhardtii] | 2.00E-24 | cre:CHLREDRAFT_188890 |  |
| Locus_266_4Transcript_1/1_Confidence_1.000_Length_1270 | 0.00E+00 | 1.84E-01 | XP_001703451.1 | lipid-binding START protein [Chlamydomonas reinhardtii] | 1.00E-107 | cre:CHLREDRAFT_188173 |  |
| TRINITY1_DN38126_c1_g15_i1 | 0.00E+00 | 1.84E-01 | XP_002958773.1 | hypothetical protein VOLCADRAFT_84639 [Volvox carteri f. nagariensis] | 0.00E+00 | vcn:VOLCADRAFT_84639 |  |
| Locus_9096_1Transcript_1/3_Confidence_0.429_Length_2404 | 1.85E-07 | 1.84E-01 | XP_013903821.1 | hypothetical protein MNEG_3155 [Monoraphidium neglectum] | 1.00E-107 |  |  |
| Locus_411_1Transcript_5/7_Confidence_0.136_Length_2557 | 0.00E+00 | 1.89E-01 | XP_013906652.1 | sulfoquinovosyltransferase [Monoraphidium neglectum] | 9.00E-38 |  |  |
| Locus_2506_1Transcript_7/10_Confidence_0.675_Length_3326 | 2.38E-07 | 1.90E-01 | XP_013898195.1 | hypothetical protein MNEG_8791 [Monoraphidium neglectum] | 1.00E-24 |  |  |
| TRINITY2_DN24739_c2_g4_i3 | 0.00E+00 | 1.92E-01 | XP_013903917.1 | hypothetical protein MNEG_3055 [Monoraphidium neglectum] | 2.00E-13 |  |  |
| Locus_1969_3Transcript_7/12_Confidence_0.192_Length_2499 | 5.24E-03 | 1.92E-01 | XP_013905210.1 | hypothetical protein MNEG_1769 [Monoraphidium neglectum] | 1.00E-11 |  |  |
| TRINITY2_DN8430_c0_g2_i1 | 0.00E+00 | 1.92E-01 | XP_001691048.1 | copper target 1 protein [Chlamydomonas reinhardtii] | 1.00E-159 |  |  |
| TRINITY1_DN32215_c0_g1_i1 | 1.03E-09 | 1.94E-01 | WP_020487648.1 | hypothetical protein [Dehalobacter sp. FTH1] | 2.00E-46 |  |  |
| TRINITY1_DN22644_c0_g1_i4 | 0.00E+00 | 1.96E-01 | XP_002950724.1 | hypothetical protein VOLCADRAFT_104839 [Volvox carteri f.nagariensis] | 9.00E-72 | vcn:VOLCADRAFT_104839 |  |
| TRINITY2_DN21972_c0_g2_i3 | 7.31E-04 | 1.96E-01 | XP_001765765.1 | predicted protein [Physcomitrella patens] | 3.00E-85 | ppp:PHYPADRAFT_129864 |  |
| Locus_694_7Transcript_4/4_Confidence_0.667_Length_1094 | 0.00E+00 | 1.97E-01 | XP_001691031.1 | light-harvesting protein of photosystem I [Chlamydomonas reinhardtii] | 4.00E-84 | cre:CHLREDRAFT_144609 |  |
| Locus_3345_10Transcript_5/6_Confidence_0.421_Length_1011 | 0.00E+00 | 1.97E-01 | XP_001701941.1 | predicted protein [Chlamydomonas reinhardtii] | 1.00E-24 | cre:CHLREDRAFT_186592 |  |
| TRINITY2_DN12087_c0_g2_i1 | 5.08E-12 | 2.02E-01 | XP_013893847.1 | hypothetical protein MNEG_13135 [Monoraphidium neglectum] | 1.00E-39 |  |  |
| comp32052_c0_seq1 | 0.00E+00 | 2.03E-01 | XP_013898997.1 | thiamine biosynthetic enzyme [Monoraphidium neglectum] | 1.00E-117 |  |  |
| TRINITY1_DN57599_c2_g1_i1 | 1.27E-07 | 2.04E-01 | XP_013903169.1 | Ankyrin repeat domain-containing protein 2 [Monoraphidium neglectum] | 2.00E-50 |  |  |
| TRINITY2_DN25788_c0_g3_i2 | 5.12E-03 | 2.04E-01 | XP_002955335.1 | hypothetical protein VOLCADRAFT_96190 [Volvox carteri f. nagariensis] | 2.00E-30 | vcn:VOLCADRAFT_96190 |  |
| TRINITY1_DN38206_c4_g2_i1 | 0.00E+00 | 2.12E-01 | XP_005852227.1 | hypothetical protein CHLNCDRAFT_133490 [Chlorella variabilis] | 1.00E-170 | cvr:CHLNCDRAFT_133490 |  |
| TRINITY1_DN60025_c2_g1_i1 | 0.00E+00 | 2.12E-01 | XP_001690058.1 | predicted protein [Chlamydomonas reinhardtii] | 1.00E-112 | cre:CHLREDRAFT_205993 |  |
| TRINITY1_DN10227_c2_g1_i1 | 3.33E-16 | 2.13E-01 | XP_013904026.1 | Uncharacterized protein ycf39 [Monoraphidium neglectum] | 1.00E-146 |  |  |
| Locus_5756_7Transcript_1/1_Confidence_1.000_Length_1012 | 0.00E+00 | 2.13E-01 | XP_013904140.1 | peroxiredoxin (alkyl hydroperoxidereductase subunit C)[Monoraphidium neglectum] | 1.00E-84 |  |  |
| comp12310_c0_seq1 | 8.40E-13 | 2.14E-01 | XP_002954026.1 | hypothetical protein VOLCADRAFT_109910 [Volvox carteri f.nagariensis] | 5.00E-38 | vcn:VOLCADRAFT_109910 |  |
| TRINITY2_DN21295_c0_g1_i1 | 1.68E-03 | 2.15E-01 | XP_001693277.1 | predicted protein [Chlamydomonas reinhardtii] | 6.00E-97 | cre:CHLREDRAFT_205939 |  |
| TRINITY1_DN5340_c0_g1_i1 | 5.81E-12 | 2.19E-01 | XP_001702107.1 | serine glyoxylate aminotransferase [Chlamydomonas reinhardtii] | 0.00E+00 |  |  |
| TRINITY2_DN25604_c0_g1_i1 | 1.00E-07 | 2.23E-01 | XP_005851030.1 | hypothetical protein CHLNCDRAFT_50471 [Chlorella variabilis] | 1.00E-38 | cvr:CHLNCDRAFT_50471 |  |
| TRINITY1_DN57659_c0_g2_i1 | 4.56E-10 | 2.27E-01 | XP_002947356.1 | hypothetical protein VOLCADRAFT_103449 [Volvox carteri f.nagariensis] | 1.00E-83 | vcn:VOLCADRAFT_103449 |  |
| TRINITY2_DN17545_c0_g1_i1 | 5.64E-06 | 2.27E-01 | XP_005847650.1 | hypothetical protein CHLNCDRAFT_52364 [Chlorella variabilis] | 2.00E-47 | cvr:CHLNCDRAFT_52364 |  |
| Locus_4958_7Transcript_1/1_Confidence_1.000_Length_991 | 4.55E-15 | 2.33E-01 | XP_002958833.1 | thylakoid membrane protein [Volvox carteri f. nagariensis] | 1.00E-29 | vcn:VOLCADRAFT_78123 |  |
| TRINITY2_DN29202_c0_g1_i1 | 1.59E-05 | 2.34E-01 | WP_051134815.1 | hypothetical protein [Ponticaulis koreensis] | 1.00E-20 |  |  |
| Locus_22688_5Transcript_1/1_Confidence_1.000_Length_949 | 0.00E+00 | 2.34E-01 | XP_001699923.1 | rhodanese-like protein [Chlamydomonas reinhardtii] | 2.00E-51 | cre:CHLREDRAFT_182149 |  |
| Locus_7783_3Transcript_2/2_Confidence_0.750_Length_1345 | 2.95E-08 | 2.34E-01 | XP_002951587.1 | hypothetical protein VOLCADRAFT_61541 [Volvox carteri f.nagariensis] | 5.00E-61 | vcn:VOLCADRAFT_61541 |  |
| TRINITY1_DN46920_c0_g1_i1 | 1.24E-08 | 2.38E-01 | XP_013901746.1 | Two-component response regulator ARR18 [Monoraphidium neglectum] | 4.00E-20 |  |  |
| TRINITY2_DN23025_c0_g2_i1 | 1.59E-09 | 2.39E-01 | XP_005851596.1 | hypothetical protein CHLNCDRAFT_56782 [Chlorella variabilis] | 1.00E-143 | cvr:CHLNCDRAFT_56782 |  |
| TRINITY1_DN52812_c0_g1_i1 | 1.95E-08 | 2.39E-01 | XP_002949328.1 | hypothetical protein VOLCADRAFT_59132, partial [Volvox carteri f.nagariensis] | 1.00E-179 | vcn:VOLCADRAFT_59132 |  |
| Locus_10368_7Transcript_1/1_Confidence_1.000_Length_1058 | 4.65E-05 | 2.40E-01 | XP_010252528.1 | PREDICTED: homeobox protein SBH1-like isoform X2 [Nelumbo nucifera] | 7.00E-26 |  |  |
| Locus_551_6Transcript_1/5_Confidence_0.429_Length_1844 | 0.00E+00 | 2.41E-01 | XP_001690638.1 | secreted protease and protease inhibitor [Chlamydomonas reinhardtii] | 7.00E-89 | cre:CHLREDRAFT_205991 |  |
| Locus_1680_4Transcript_1/1_Confidence_1.000_Length_557 | 4.73E-11 | 2.41E-01 | XP_002952640.1 | hypothetical protein VOLCADRAFT_105627 [Volvox carteri f.nagariensis] | 2.00E-70 | vcn:VOLCADRAFT_105627 |  |
| Locus_552_7Transcript_4/10_Confidence_0.138_Length_1123 | 2.85E-10 | 2.43E-01 | WP_034967263.1 | hypothetical protein [Campylobacter mucosalis] | 1.00E-12 |  |  |
| Locus_115_4Transcript_1/1_Confidence_1.000_Length_1276 | 0.00E+00 | 2.44E-01 | XP_013899415.1 | thylakoid formation protein 1 [Monoraphidium neglectum] | 5.00E-67 |  |  |
| TRINITY1_DN53271_c0_g1_i1 | 2.96E-03 | 2.49E-01 | XP_001694949.1 | aspartyl-tRNA synthetase [Chlamydomonas reinhardtii] | 0.00E+00 |  |  |
| TRINITY2_DN26826_c0_g4_i1 | 8.87E-06 | 2.52E-01 | XP_010473050.1 | PREDICTED: SPX domain-containing protein 2-like [Camelina sativa] | 3.00E-29 |  |  |
| Locus_11049_9Transcript_1/1_Confidence_1.000_Length_444 | 2.85E-05 | 2.52E-01 | XP_013903778.1 | Transcription factor bHLH34 [Monoraphidium neglectum] | 3.00E-27 |  |  |
| TRINITY1_DN55271_c1_g1_i1 | 2.22E-16 | 2.53E-01 | XP_002956124.1 | hypothetical protein VOLCADRAFT_109995 [Volvox carteri f.nagariensis] | 2.00E-59 | vcn:VOLCADRAFT_109995 |  |
| TRINITY2_DN8686_c0_g1_i1 | 7.10E-03 | 2.57E-01 | XP_002958587.1 | hypothetical protein VOLCADRAFT_84511 [Volvox carteri f.nagariensis] | 4.00E-58 | vcn:VOLCADRAFT_84511 |  |
| TRINITY2_DN20993_c0_g2_i2 | 2.42E-08 | 2.58E-01 | XP_005645388.1 | DUF506-domain-containing protein [Coccomyxa subellipsoidea C-169] | 5.00E-15 | csl:COCSUDRAFT_48431 |  |
| TRINITY2_DN38809_c0_g1_i1 | 0.00E+00 | 2.60E-01 | XP_001694345.1 | small protein associating with GAPDH and PRK [Chlamydomonasreinhardtii] | 7.00E-14 | cre:CHLREDRAFT_148487 |  |
| Locus_3291_8Transcript_1/1_Confidence_1.000_Length_1148 | 9.99E-16 | 2.75E-01 | XP_001697671.1 | hypothetical protein CHLREDRAFT_185012 [Chlamydomonas reinhardtii] | 1.00E-32 | cre:CHLREDRAFT_185012 |  |
| TRINITY1_DN20118_c0_g1_i1 | 1.42E-13 | 2.76E-01 | XP_002948802.1 | hypothetical protein VOLCADRAFT_104013 [Volvox carteri f.nagariensis] | 7.00E-60 | vcn:VOLCADRAFT_104013 |  |
| TRINITY2_DN23009_c0_g1_i5 | 1.51E-02 | 2.80E-01 | XP_001693241.1 | hypothetical protein CHLREDRAFT_205900 [Chlamydomonas reinhardtii] | 1.00E-71 | cre:CHLREDRAFT_205900 |  |
| Locus_1309_4Transcript_1/1_Confidence_1.000_Length_723 | 0.00E+00 | 2.83E-01 | XP_005650753.1 | plastid acyl carrier protein [Coccomyxa subellipsoidea C-169] | 1.00E-29 | csl:COCSUDRAFT_52715 |  |
| TRINITY1_DN35456_c5_g1_i3 | 1.43E-03 | 2.84E-01 | WP_007625417.1 | purple acid phosphatase 17 [Paraglaciecola arctica] | 2.00E-13 |  |  |
| Locus_10864_8Transcript_1/1_Confidence_1.000_Length_834 | 3.79E-07 | 2.85E-01 | XP_001695838.1 | FKBP-type peptidyl-prolyl cis-trans isomerase [Chlamydomonasreinhardtii] | 5.00E-43 | cre:CHLREDRAFT_195500 |  |
| comp6168_c0_seq2 | 0.00E+00 | 2.85E-01 | XP_001693656.1 | predicted protein, partial [Chlamydomonas reinhardtii] | 1.00E-119 | cre:CHLREDRAFT_48787 |  |
| TRINITY2_DN47003_c0_g1_i1 | 0.00E+00 | 2.97E-01 | XP_010063529.1 | PREDICTED: COBW domain-containing protein 1 [Eucalyptus grandis] | 4.00E-98 | egr:104450601 |  |
| TRINITY2_DN24403_c0_g3_i2 | 4.29E-03 | 2.99E-01 | XP_004490293.1 | PREDICTED: importin-9 [Cicer arietinum] | 8.00E-87 | cam:101497018 |  |
| Locus_1655_1Transcript_1/1_Confidence_1.000_Length_1358 | 1.96E-03 | 2.99E-01 | XP_001699886.1 | intramembrane metalloprotease [Chlamydomonas reinhardtii] | 8.00E-54 | cre:CHLREDRAFT_141639 |  |
| TRINITY1_DN50655_c0_g1_i1 | 0.00E+00 | 3.00E-01 | XP_001690873.1 | magnesium chelatase subunit I [Chlamydomonas reinhardtii] | 1.00E-143 | cre:CHLREDRAFT_135584 |  |
| comp46970_c0_seq1 | 3.65E-03 | 3.00E-01 | XP_013897266.1 | hypothetical protein MNEG_9716 [Monoraphidium neglectum] | 2.00E-25 |  |  |
| TRINITY1_DN45070_c0_g1_i3 | 0.00E+00 | 3.04E-01 | XP_009339774.1 | PREDICTED: calvin cycle protein CP12-1, chloroplastic-like [Pyrus xbretschneideri] | 6.00E-11 | pxb:103931969 |  |
| comp35761_c0_seq1 | 0.00E+00 | 3.07E-01 | XP_001694485.1 | plastid ribosomal protein S1 [Chlamydomonas reinhardtii] | 1.00E-115 | cre:CHLREDRAFT_79955 |  |
| Locus_1990_7Transcript_1/2_Confidence_0.667_Length_1406 | 2.28E-07 | 3.08E-01 | XP_005651871.1 | cation efflux protein [Coccomyxa subellipsoidea C-169] | 2.00E-76 | csl:COCSUDRAFT_21325 |  |
| TRINITY2_DN6681_c0_g2_i1 | 4.44E-08 | 3.09E-01 | XP_002953712.1 | hypothetical protein VOLCADRAFT_106063 [Volvox carteri f.nagariensis] | 1.00E-111 | vcn:VOLCADRAFT_106063 |  |
| Locus_6664_3Transcript_1/1_Confidence_1.000_Length_1172 | 1.10E-02 | 3.10E-01 | XP_002958370.1 | hypothetical protein VOLCADRAFT_119932, partial [Volvox carteri f.nagariensis] | 1.00E-24 | vcn:VOLCADRAFT_119932 |  |
| Locus_2841_6Transcript_1/1_Confidence_1.000_Length_957 | 6.59E-06 | 3.13E-01 | XP_002948868.1 | plastid/chloroplast ribosomal protein L7/L12 [Volvox carteri f.nagariensis] | 5.00E-33 | vcn:VOLCADRAFT_80383 |  |
| Locus_236_3Transcript_3/9_Confidence_0.281_Length_1249 | 8.12E-04 | 3.28E-01 | XP_001693115.1 | predicted protein [Chlamydomonas reinhardtii] | 7.00E-65 | cre:CHLREDRAFT_190448 |  |
| TRINITY1_DN55444_c0_g1_i1 | 2.76E-02 | 3.29E-01 | XP_005645860.1 | hypothetical protein COCSUDRAFT_56538 [Coccomyxa subellipsoideaC-169] | 4.00E-68 | csl:COCSUDRAFT_56538 |  |
| Locus_4504_7Transcript_1/1_Confidence_1.000_Length_1128 | 2.79E-06 | 3.31E-01 | XP_002951388.1 | hypothetical protein VOLCADRAFT_120951 [Volvox carteri f.nagariensis] | 2.00E-48 | vcn:VOLCADRAFT_120951 |  |
| TRINITY2_DN10787_c0_g1_i1 | 1.11E-03 | 3.31E-01 | WP_014549029.1 | lipase [Francisella tularensis] | 4.00E-11 |  |  |
| comp30677_c7_seq1 | 0.00E+00 | 3.34E-01 | XP_002956367.1 | peroxiredoxin, type II [Volvox carteri f. nagariensis] | 2.00E-64 | vcn:VOLCADRAFT_107207 |  |
| TRINITY1_DN8855_c0_g1_i1 | 1.27E-09 | 3.36E-01 | XP_002948891.1 | hypothetical protein VOLCADRAFT_58555 [Volvox carteri f. nagariensis] | 1.00E-144 | vcn:VOLCADRAFT_58555 |  |
| TRINITY1_DN47891_c23_g6_i1 | 3.59E-03 | 3.36E-01 | XP_005842981.1 | hypothetical protein CHLNCDRAFT_141678 [Chlorella variabilis] | 5.00E-19 | cvr:CHLNCDRAFT_141678 |  |
| TRINITY1_DN8866_c0_g1_i2 | 1.17E-05 | 3.38E-01 | XP_001696942.1 | polyprotein of PSRP-7 and EF-Ts, splice variant 2 [Chlamydomonasreinhardtii] | 4.00E-59 |  |  |
| comp17950_c0_seq2 | 7.29E-13 | 3.38E-01 | XP_001698375.1 | dehydroascorbate reductase [Chlamydomonas reinhardtii] | 4.00E-47 | cre:CHLREDRAFT_143082 |  |
| TRINITY2_DN48257_c0_g1_i1 | 4.36E-10 | 3.39E-01 | XP_013900795.1 | putative membrane-associated protein, chloroplastic Flags: Precursor[Monoraphidium neglectum] | 2.00E-96 |  |  |
| Locus_14740_10Transcript_1/1_Confidence_1.000_Length_1111 | 1.86E-07 | 3.43E-01 | XP_001695315.1 | predicted protein [Chlamydomonas reinhardtii] | 2.00E-72 | cre:CHLREDRAFT_158401 |  |
| TRINITY2_DN12969_c0_g2_i1 | 9.31E-08 | 3.50E-01 | XP_001699105.1 | hypothetical protein CHLREDRAFT_193550 [Chlamydomonas reinhardtii] | 1.00E-93 | cre:CHLREDRAFT_193550 |  |
| Locus_5483_3Transcript_6/6_Confidence_0.706_Length_1659 | 1.08E-02 | 3.52E-01 | XP_013898471.1 | hypothetical protein MNEG_8511 [Monoraphidium neglectum] | 7.00E-43 |  |  |
| TRINITY2_DN29265_c0_g1_i1 | 1.68E-03 | 3.57E-01 | XP_013906154.1 | 50S ribosomal protein L11 [Monoraphidium neglectum] | 1.00E-59 |  |  |
| TRINITY2_DN26277_c3_g1_i3 | 4.58E-09 | 3.58E-01 | XP_002946024.1 | hypothetical protein VOLCADRAFT_120148 [Volvox carteri f.nagariensis] | 1.00E-155 | vcn:VOLCADRAFT_120148 |  |
| Locus_6135_8Transcript_1/1_Confidence_1.000_Length_1157 | 4.94E-02 | 3.75E-01 | XP_013901915.1 | Rubredoxin [Monoraphidium neglectum] | 2.00E-40 |  |  |
| TRINITY1_DN48461_c2_g1_i1 | 9.12E-05 | 3.83E-01 | XP_013906000.1 | Thylakoid lumenal protein 1 [Monoraphidium neglectum] | 1.00E-36 |  |  |
| Locus_2112_4Transcript_1/1_Confidence_1.000_Length_781 | 2.68E-03 | 3.84E-01 | XP_001701609.1 | SOUL heme-binding protein [Chlamydomonas reinhardtii] | 1.00E-63 | cre:CHLREDRAFT_21100 |  |
| TRINITY2_DN17091_c0_g2_i1 | 3.61E-02 | 3.87E-01 | XP_013906797.1 | Replication protein A DNA-binding subunit [Monoraphidium neglectum] | 5.00E-45 |  |  |
| TRINITY2_DN26395_c1_g3_i3 | 2.15E-03 | 3.94E-01 | XP_002945715.1 | hypothetical protein VOLCADRAFT_85973 [Volvox carteri f. nagariensis] | 3.00E-61 | vcn:VOLCADRAFT_85973 |  |
| TRINITY1_DN43783_c3_g1_i1 | 4.20E-14 | 3.96E-01 | XP_002954972.1 | hypothetical protein VOLCADRAFT_121362 [Volvox carteri f.nagariensis] | 5.00E-45 | vcn:VOLCADRAFT_121362 |  |
| Locus_3575_8Transcript_1/2_Confidence_0.857_Length_974 | 3.58E-02 | 3.97E-01 | XP_013901804.1 | Glycine-rich RNA-binding protein 10 [Monoraphidium neglectum] | 8.00E-18 |  |  |
| Locus_3803_10Transcript_5/5_Confidence_0.455_Length_1829 | 3.12E-07 | 4.01E-01 | XP_002952024.1 | hypothetical protein VOLCADRAFT_105335 [Volvox carteri f.nagariensis] | 5.00E-56 | vcn:VOLCADRAFT_105335 |  |
| Locus_1927_4Transcript_1/1_Confidence_1.000_Length_1397 | 0.00E+00 | 4.07E-01 | XP_002946085.1 | hypothetical protein VOLCADRAFT_102702 [Volvox carteri f.nagariensis] | 4.00E-89 | vcn:VOLCADRAFT_102702 |  |
| TRINITY2_DN29392_c0_g1_i1 | 1.67E-02 | 4.09E-01 | XP_002955883.1 | hypothetical protein VOLCADRAFT_106968 [Volvox carteri f.nagariensis] | 2.00E-21 | vcn:VOLCADRAFT_106968 |  |
| TRINITY2_DN329_c0_g1_i1 | 4.09E-06 | 4.10E-01 | XP_007512115.1 | rhodanese-like domain protein [Bathycoccus prasinos] | 2.00E-19 | bpg:Bathy07g02390 |  |
| Locus_931_3Transcript_4/5_Confidence_0.500_Length_1180 | 2.36E-04 | 4.12E-01 | XP_005650929.1 | hypothetical protein COCSUDRAFT_46068 [Coccomyxa subellipsoideaC-169] | 7.00E-59 | csl:COCSUDRAFT_46068 |  |
| comp32673_c0_seq1 | 0.00E+00 | 4.17E-01 | XP_002946718.1 | hypothetical protein VOLCADRAFT_79300 [Volvox carteri f. nagariensis] | 1.00E-62 | vcn:VOLCADRAFT_79300 |  |
| TRINITY2_DN26389_c7_g6_i2 | 1.56E-02 | 4.20E-01 | XP_005651478.1 | hypothetical protein COCSUDRAFT_12206, partial [Coccomyxasubellipsoidea C-169] | 1.00E-63 | csl:COCSUDRAFT_12206 |  |
| Locus_249_4Transcript_1/1_Confidence_1.000_Length_783 | 6.09E-13 | 4.26E-01 | XP_002953213.1 | hypothetical protein VOLCADRAFT_105826 [Volvox carteri f.nagariensis] | 3.00E-32 | vcn:VOLCADRAFT_105826 |  |
| Locus_5754_10Transcript_4/10_Confidence_0.457_Length_3769 | 1.14E-02 | 4.35E-01 | XP_010543033.1 | PREDICTED: uncharacterized protein LOC104816062 [Tarenayahassleriana] | 2.00E-11 | thj:104816062 |  |
| Locus_1573_10Transcript_1/1_Confidence_1.000_Length_1503 | 2.35E-07 | 4.35E-01 | XP_002956688.1 | hypothetical protein VOLCADRAFT_107336 [Volvox carteri f.nagariensis] | 6.00E-67 | vcn:VOLCADRAFT_107336 |  |
| Locus_7552_10Transcript_1/1_Confidence_1.000_Length_1283 | 3.05E-05 | 4.38E-01 | XP_001691396.1 | zygote-specific protein [Chlamydomonas reinhardtii] | 4.00E-90 | cre:CHLREDRAFT_144935 |  |
| Locus_1026_3Transcript_1/1_Confidence_1.000_Length_2551 | 5.13E-12 | 4.43E-01 | XP_013906163.1 | acetyl-CoA carboxylase, biotin carboxylase [Monoraphidium neglectum] | 0.00E+00 |  |  |
| TRINITY2_DN37492_c0_g1_i1 | 2.54E-04 | 4.43E-01 | XP_013899506.1 | Putative apospory-associated protein C [Monoraphidium neglectum] | 1.00E-128 |  |  |
| TRINITY1_DN47016_c8_g1_i1 | 2.52E-02 | 4.56E-01 | XP_001700220.1 | predicted protein [Chlamydomonas reinhardtii] | 4.00E-72 | cre:CHLREDRAFT_194003 |  |
| TRINITY1_DN8866_c0_g1_i1 | 2.22E-16 | 4.58E-01 | XP_001696943.1 | polyprotein of PSRP-7 and EF-Ts, imported to chloroplast[Chlamydomonas reinhardtii] | 2.00E-88 |  |  |
| Locus_5948_5Transcript_3/3_Confidence_0.600_Length_2261 | 1.47E-04 | 4.65E-01 | XP_002946204.1 | hypothetical protein VOLCADRAFT_102805 [Volvox carteri f.nagariensis] | 1.00E-158 | vcn:VOLCADRAFT_102805 |  |
| TRINITY2_DN26343_c0_g3_i3 | 3.93E-02 | 4.65E-01 | XP_002946662.1 | hypothetical protein VOLCADRAFT_103078 [Volvox carteri f.nagariensis] | 1.00E-109 | vcn:VOLCADRAFT_103078 |  |
| TRINITY1_DN57609_c0_g1_i1 | 1.06E-11 | 4.66E-01 | XP_005844483.1 | hypothetical protein CHLNCDRAFT_26777 [Chlorella variabilis] | 1.00E-127 | cvr:CHLNCDRAFT_26777 |  |
| TRINITY1_DN30302_c0_g4_i1 | 1.59E-02 | 4.66E-01 | XP_013906255.1 | Carbohydrate/purine kinase [Monoraphidium neglectum] | 1.00E-95 |  |  |
| TRINITY1_DN44671_c0_g1_i2 | 4.16E-02 | 4.69E-01 | XP_009794608.1 | PREDICTED: histone H4 isoform X1 [Nicotiana sylvestris] | 6.00E-43 |  |  |
| Locus_1456_8Transcript_1/1_Confidence_1.000_Length_895 | 3.33E-16 | 4.82E-01 | XP_001702473.1 | predicted protein [Chlamydomonas reinhardtii] | 4.00E-12 | cre:CHLREDRAFT_140850 |  |
| TRINITY2_DN4368_c0_g1_i1 | 1.75E-03 | 4.88E-01 | XP_013904361.1 | hypothetical protein MNEG_2618 [Monoraphidium neglectum] | 4.00E-55 |  |  |
| Locus_2015_4Transcript_1/1_Confidence_1.000_Length_796 | 4.92E-03 | 4.95E-01 | XP_001690777.1 | hypothetical protein CHLREDRAFT_144472 [Chlamydomonas reinhardtii] | 4.00E-19 | cre:CHLREDRAFT_144472 |  |
| Locus_2053_5Transcript_2/4_Confidence_0.625_Length_1570 | 8.84E-12 | 5.00E-01 | XP_005851162.1 | hypothetical protein CHLNCDRAFT_137777 [Chlorella variabilis] | 1.00E-30 | cvr:CHLNCDRAFT_137777 |  |
