# Supplementary material for: Exploring the transcriptome of non-model oleaginous microalga Dunaliella tertiolecta through high-throughput sequencing and high performance computing
Source: BMC Bioinformatics. 2017 Feb 22;18:122. doi: 10.1186/s12859-017-1551-x (PMC5322580; doi:10.1186/s12859-017-1551-x)
Supplement: Additional file 4: — KEGG analyses from Dt_v10 and Dt_v11. (a) Dt_v10 analysis; (b) Dt_v11 analysis. (DOCX 65 kb) [file 12859_2017_1551_MOESM4_ESM.docx]

**Additional file 4 - KEGG analyses from Dt_v10 and Dt_v11.**

(a) Dt_v10

| **Pathway Name** | **Enrichment p-value** |
| --- | --- |
| Metabolic pathways | 9.57E-14 |
| Biosynthesis of secondary metabolites | 1.72E-13 |
| Carbon metabolism | 1.95E-12 |
| Energy Metabolism | 3.02E-12 |
| Photosynthesis - antenna proteins | 1.66E-08 |
| Porphyrin and chlorophyll metabolism | 1.39E-07 |
| Citrate cycle (TCA cycle) | 1.10E-05 |
| Alanine, aspartate and glutamate metabolism | 3.18E-05 |
| Glycine, serine and threonine metabolism | 4.06E-05 |
| Glyoxylate and dicarboxylate metabolism | 1.71E-04 |
| Photosynthesis | 2.26E-04 |
| Pyruvate metabolism | 2.56E-04 |
| Biosynthesis of amino acids | 2.65E-04 |
| Carbon fixation in photosynthetic organisms | 1.60E-03 |
| Propanoate metabolism | 2.41E-03 |
| Glycolysis / Gluconeogenesis | 3.06E-03 |
| One carbon pool by folate | 7.20E-03 |

(b) Dt_v11

| **Pathway Name** | **Enrichment p-value** |
| --- | --- |
| Metabolic pathways | 0.00E+00 |
| Biosynthesis of secondary metabolites | 0.00E+00 |
| Carbon metabolism | 0.00E+00 |
| Porphyrin and chlorophyll metabolism | 5.56E-15 |
| Citrate cycle (TCA cycle) | 6.92E-14 |
| Photosynthesis | 2.83E-11 |
| Pyruvate metabolism | 4.73E-09 |
| Photosynthesis - antenna proteins | 8.81E-09 |
| Glycolysis / Gluconeogenesis | 2.56E-07 |
| Alanine, aspartate and glutamate metabolism | 4.43E-07 |
| Fatty acid biosynthesis | 4.71E-07 |
| Glyoxylate and dicarboxylate metabolism | 7.17E-07 |
| Glycine, serine and threonine metabolism | 3.84E-06 |
| Biosynthesis of amino acids | 9.14E-06 |
| Carbon fixation in photosynthetic organisms | 1.11E-05 |
| Fatty acid metabolism | 9.24E-05 |
| Nitrogen metabolism | 9.69E-05 |
| Biotin metabolism | 8.77E-04 |
| Terpenoid backbone biosynthesis | 1.42E-03 |
| Propanoate metabolism | 1.69E-03 |
| Pentose phosphate pathway | 1.20E-02 |
| Oxidative phosphorylation | 1.24E-02 |
| 2-Oxocarboxylic acid metabolism | 2.10E-02 |
| DNA replication | 2.83E-02 |
| Insulin resistance | 4.41E-02 |
| Starch and sucrose metabolism | 4.57E-02 |
