# Supplementary material for: Exploring the transcriptome of non-model oleaginous microalga Dunaliella tertiolecta through high-throughput sequencing and high performance computing
Source: BMC Bioinformatics. 2017 Feb 22;18:122. doi: 10.1186/s12859-017-1551-x (PMC5322580; doi:10.1186/s12859-017-1551-x)
Supplement: Additional file 5: — GO analysis list. (DOCX 92 kb) [file 12859_2017_1551_MOESM5_ESM.docx]

**Additional file 5 - GO analysis list.**

| **GO function** | **Enrichment Score** | **Enrichment p-value** | **GO ID** | **Up/Down** |
| --- | --- | --- | --- | --- |
| metabolic process | 17.0715 | 3.85E-08 | 8152 | Up |
| amino acid binding | 10.473 | 2.83E-05 | 16597 | Up |
| nitrogen compound metabolic process | 9.57267 | 6.96E-05 | 6807 | Up |
| transport | 9.16975 | 1.04E-04 | 6810 | Up |
| tricarboxylic acid cycle | 8.72209 | 1.63E-04 | 6099 | Up |
| catalytic activity | 8.67485 | 1.71E-04 | 3824 | Up |
| transporter activity | 7.50178 | 5.52E-04 | 5215 | Up |
| L-serine biosynthetic process | 7.36453 | 6.33E-04 | 6564 | Up |
| biotin binding | 7.36453 | 6.33E-04 | 9374 | Up |
| glycolysis | 7.28794 | 6.84E-04 | 6096 | Up |
| succinate dehydrogenase (ubiquinone) activity | 6.17982 | 2.07E-03 | 8177 | Up |
| photosynthesis | 31.3567 | 2.41E-14 | 15979 | Down |
| photosynthesis, light harvesting | 30.171 | 7.89E-14 | 9765 | Down |
| chlorophyll biosynthetic process | 17.415 | 2.73E-08 | 15995 | Down |
| tetrapyrrole biosynthetic process | 15.4687 | 1.91E-07 | 33014 | Down |
| fatty acid biosynthetic process | 13.4353 | 1.46E-06 | 6633 | Down |
| oxygen evolving complex | 11.1222 | 1.48E-05 | 9654 | Down |
| antioxidant activity | 10.3004 | 3.36E-05 | 16209 | Down |
| peptidyl-prolyl cis-trans isomerase activity | 10.1199 | 4.03E-05 | 3755 | Down |
| oxidoreductase activity | 9.69716 | 6.15E-05 | 16491 | Down |
| ribosome | 9.62806 | 6.59E-05 | 5840 | Down |
| protein folding | 9.60134 | 6.76E-05 | 6457 | Down |
| structural constituent of ribosome | 9.35395 | 8.66E-05 | 3735 | Down |
| magnesium chelatase activity | 8.96604 | 1.28E-04 | 16851 | Down |
| extrinsic to membrane | 8.96604 | 1.28E-04 | 19898 | Down |
| peroxiredoxin activity | 8.96604 | 1.28E-04 | 51920 | Down |
| FAD binding | 8.40943 | 2.23E-04 | 50660 | Down |
| membrane | 8.29559 | 2.50E-04 | 16020 | Down |
| one-carbon compound metabolic process | 8.07379 | 3.12E-04 | 6730 | Down |
| methionine biosynthetic process | 8.07379 | 3.12E-04 | 9086 | Down |
| translation | 7.53033 | 5.37E-04 | 6412 | Down |
| porphyrin biosynthetic process | 7.40465 | 6.08E-04 | 6779 | Down |
| FK506-sensitive peptidyl-prolyl cis-trans isomerase | 7.04112 | 8.75E-04 | 30051 | Down |
| cyclophilin-type peptidyl-prolyl cis-trans isomerase activity | 7.04112 | 8.75E-04 | 42027 | Down |
| cyclophilin | 7.04112 | 8.75E-04 | 4600 | Down |
| P-P-bond-hydrolysis-driven protein transmembrane transporter activity | 6.42292 | 1.62E-03 | 15450 | Down |
| ATP synthesis coupled proton transport | 6.1089 | 2.22E-03 | 15986 | Down |
| acetyl-CoA carboxylase activity | 5.80435 | 3.01E-03 | 9317 | Down |
| glycine catabolic process | 5.80435 | 3.01E-03 | 6546 | Down |
| inorganic anion transport | 5.80435 | 3.01E-03 | 15698 | Down |
| galactosyltransferase activity | 5.14998 | 5.80E-03 | 8378 | Down |
| thiamin biosynthetic process | 5.13261 | 5.90E-03 | 9228 | Down |
| terpenoid biosynthetic process | 5.13261 | 5.90E-03 | 16114 | Down |
| photosystem I | 5.13261 | 5.90E-03 | 9522 | Down |
| glycine metabolic process | 5.13261 | 5.90E-03 | 6544 | Down |
| electron transport | 5.03363 | 6.52E-03 | 6118 | Down |
| cell redox homeostasis | 5.00802 | 6.68E-03 | 45454 | Down |
| intracellular | 4.79657 | 8.26E-03 | 5622 | Down |
| fructose-bisphosphate aldolase activity | 4.64313 | 9.63E-03 | 4332 | Down |
| adenylate kinase activity | 4.64313 | 9.63E-03 | 4017 | Down |
| protein secretion | 4.64313 | 9.63E-03 | 9306 | Down |
| photosystem I reaction center | 4.64313 | 9.63E-03 | 9538 | Down |
