# Supplementary material for: Exploring the transcriptome of non-model oleaginous microalga Dunaliella tertiolecta through high-throughput sequencing and high performance computing
Source: BMC Bioinformatics. 2017 Feb 22;18:122. doi: 10.1186/s12859-017-1551-x (PMC5322580; doi:10.1186/s12859-017-1551-x)
Supplement: Additional file 6: — Integration of significant genes hit in KEGG biological pathway analysis from Dt_v10 and Dt_v11. With red color boxes mean upregulation, and green mean downregulation. The value in the bracket means the fold change of gene expression level in the study of ND/replete while using either v10 or v11 database. (a) Photosynthesis; (b) Photosynthesis - Antenna proteins; (c) Citrate cycle (TCA cycle); (d) Pyruvate metabolism; (e) Glycolysis/Gluconeogenesis. (DOCX 987 kb) [file 12859_2017_1551_MOESM6_ESM.docx]

**Additional file 6 - Integration of significant genes hit in KEGG biological pathway analysis from Dt_v10 and Dt_v11.**

With red color boxes mean upregulation, and green mean downregulation. The value in the bracket means the fold change of gene expression level in the study of ND/replete while using either v10 or v11 database.

(a) Photosynthesis; (b) Photosynthesis - Antenna proteins; (c) Citrate cycle (TCA cycle); (d) Pyruvate metabolism; (e) Glycolysis/ Gluconeogenesis.

a

b

c

d

e
